# Supplementary material for: Interferon-stimulated neutrophils as a predictor of immunotherapy response
Source: Cancer Cell. 2024 Feb 12;42(2):253–265.e12. doi: 10.1016/j.ccell.2023.12.005 (PMC10864002; doi:10.1016/j.ccell.2023.12.005)
Supplement: Document S1. Figures S1–S10 and Tables S3 and S4 [file mmc1.pdf]

Cancer Cell, Volume *42*

Supplemental information

Interferon-stimulated neutrophils

as a predictor of immunotherapy response

Madeleine Benguigui, Tim J. Cooper, Prajakta Kalkar, Sagie Schif-Zuck, Ruth Halaban, Antonella Bacchiocchi, Iris Kamer, Abhilash Deo, Bar Manobla, Rotem Menachem, Jozafina Haj-Shomaly, Avital Vorontsova, Ziv Raviv, Chen Buxbaum, Petros Christopoulos, Jair Bar, Michal Lotem, Mario Sznol, Amiram Ariel, Shai S. Shen-Orr, and Yuval Shaked

SUPPLEMENTARY DATA

Supplementary Tables

Table S3. List of tumor models used, related to Figure 1.

Cell Line	Clones	Cancer Type	Mouse Strain	Model Category
4T1	$4T1_P$, $4T1_M$	Breast Carcinoma	BALB/c	Mutagenesis
RENCA	$RENCA_P$, $RENCA_M$	Renal Adenocarcinoma	BALB/c	Mutagenesis
LLC	LLC_P, LLC_M	Lung Carcinoma	C57BL/6	Mutagenesis
EMT6	$EMT6_P$	Breast Carcinoma	BALB/c	Spontaneous
LLC	LLC_P	Lung Carcinoma	C57BL/6 x CBA	Backcross

The initial three rows (mutagenesis) are tumor-dependent models. The remaining rows are host-dependent models. *P = parental cells, M = mutagenized cells.*

Table S4. List of primers used for RT-qPCR, related to Figure 4.

Gene	Forward	Reverse
mTnfα	CTGAACTTCGGGGTGATCGG	GGCTTGTCACTCGAATTTTGAGA
mCxcl1	CTGGGATTCACCTCAAGAACATC	CAGGGTCAAGGCAAGCCTC
mIL1β	TCTCAGATTCACAACTGTTCGTG	AGAAAATGAGGTCGGTCTCACTA
mIL23a	CAGCAGCTCTCTCGGAATCTC	TGGATACGGGGCACATTATTTTT
mSaa3	TGCCATCATTCTTTGCATCTTGA	CCGTGAACTTCTGAACAGCCT
mCcl3	TGTACCATGACACTCTGCAAC	CAACGATGAATTGGCGTGGAA
mCcl6	AAGAAGATCGTCGCTATAACCCT	GCTTAGGCACCTCTGAACTCTC

Supplementary Figures
Figure S1.

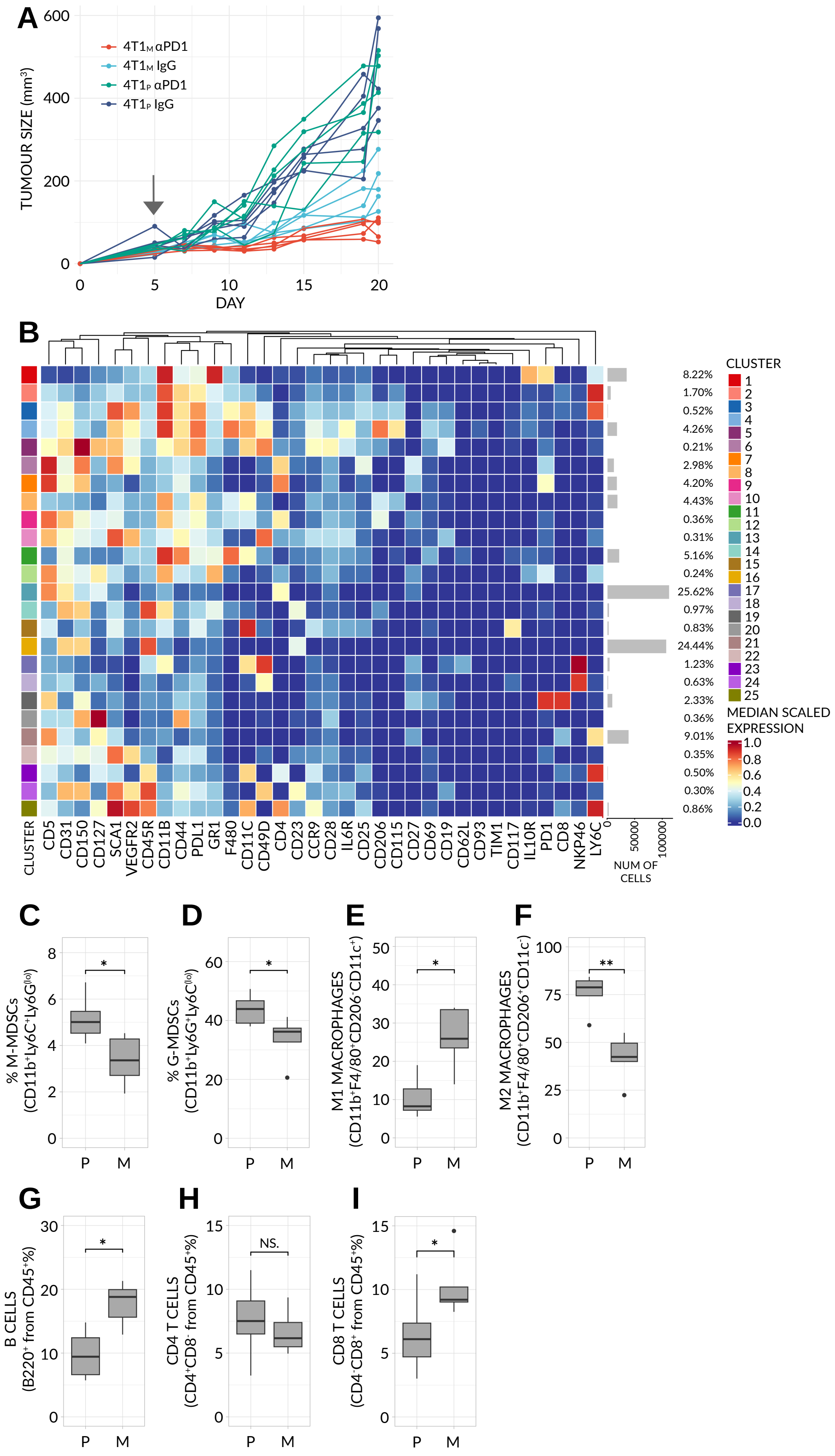

Figure S1. Mutagenized 4T1 tumors support an immunogenic TME, related to Figure 2. **(A)** Raw, individual tumor growth profiles of BALB/c mice implanted with parental $4T1_P$ (P) or mutagenized $4T1_M$ (M) breast cancer and treated with αPD1 or control IgG antibodies (n=5 mice/group). Treatment was initiated at a tumor size of ~$50mm^3$ (arrow). **(B)** $CD45^+$ cells from the tumor microenvironment (TME) of $4T1_P$ (205,678 cells) and $4T1_M$ (236,251 cells) tumors were segregated into 25 distinct, unsupervised clusters (n=5 mice pooled/group). A heatmap of the scaled, median expression level of each CYTOF marker is shown and was used to annotate each cluster. The frequency of each cluster, calculated as a percentage of total $CD45^+$ cells, is shown as a bar-plot (right). ***(C-I)*** Frequencies of: monocytic MDSCs (M-MDSCs) **(C)**; granulocytic MDSCs (G-MDSCs) **(D)**; M1 macrophages **(E)**; M2 macrophages **(F)**; B cells **(G)**; $CD4^+$ T cells **(H)**; $CD8^+$ T cells **(I)** in the TME of untreated 4T1 tumors, as determined by flow cytometry (n=5 mice/group). *In (C-I), significance was assessed by means of a one-way Mann-Whitney test (NS, p>0.01; *, p<0.01; **, p<0.001).*

Figure S2.

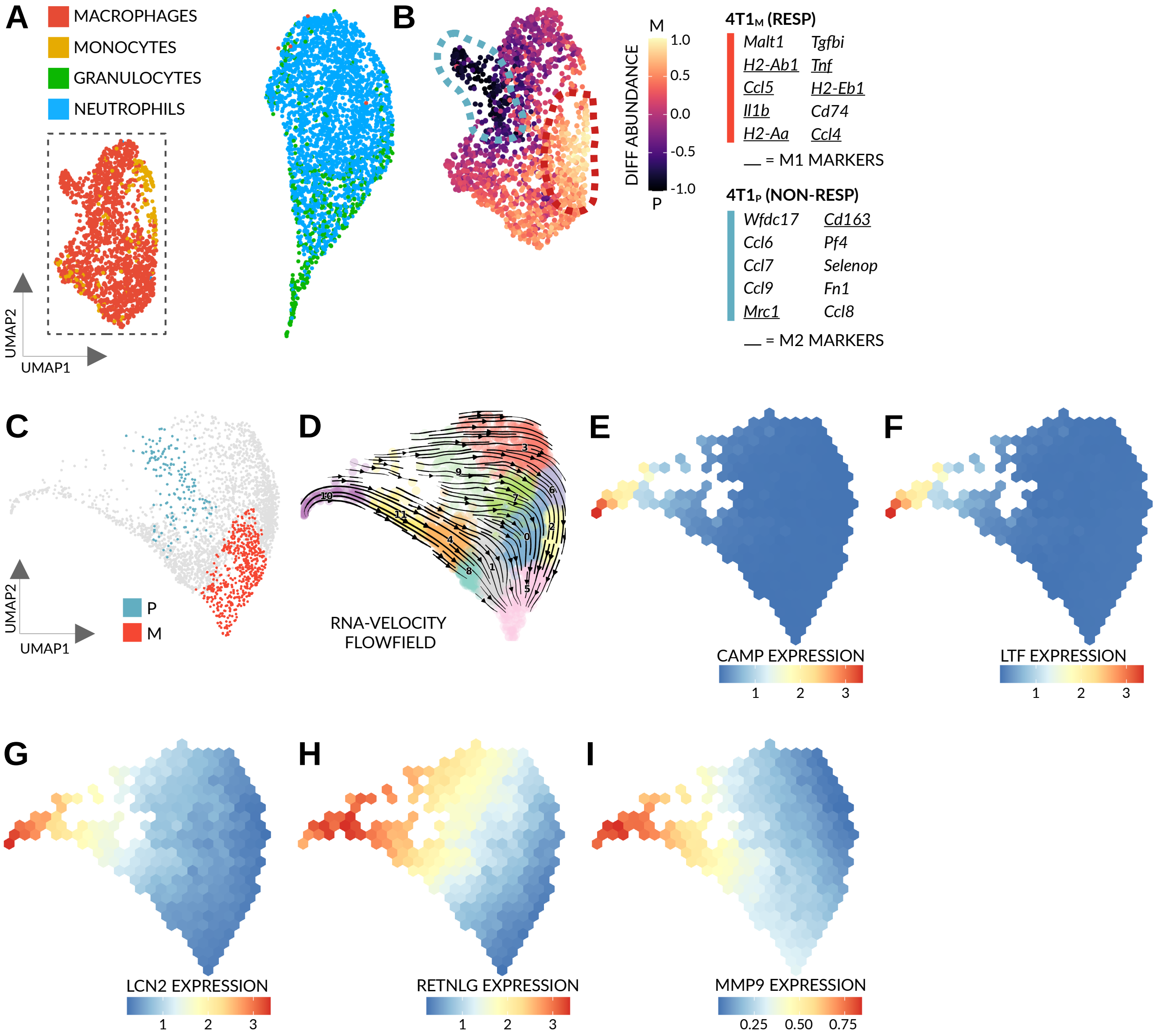
A
MACROPHAGES
MONOCYTES
GRANULOCYTES
NEUTROPHILS
UMAP2
UMAP1
B
M
P
DIFF ABUNDANCE
1.0
0.5
0.0
-0.5
-1.0
4T1M (RESP)
Malt1 Tgfbi
H2-Ab1 Tnf
Ccl5 H2-Eb1
Il1b Cd74
H2-Aa Ccl4
= M1 MARKERS
4T1P (NON-RESP)
Wfdc17 Cd163
Ccl6 Pf4
Ccl7 Selenop
Ccl9 Fn1
Mrc1 Ccl8
= M2 MARKERS
C
UMAP2
UMAP1
P
M
D
RNA-VELOCITY FLOWFIELD
E
CAMP EXPRESSION
1 2 3
F
LTF EXPRESSION
1 2 3
G
LCN2 EXPRESSION
1 2 3
H
RETNLG EXPRESSION
1 2 3
I
MMP9 EXPRESSION
0.25 0.50 0.75

Figure S2. scRNA-seq of GR1⁺ cells from 4T1 tumors, related to Figure 3. 10X scRNA-seq was performed on GR1$^+$ cells obtained from 4T1 breast cancer parental ($4T1_P$) (non-responsive) and mutagenized ($4T1_M$) (responsive) tumors (n=3 mice pooled/group). Data is identical to that used in Fig 3. **(A)** UMAP plot of 4711 filtered, GR1$^+$ cells from which 1825 cells are monocytes ($4T1_P$ = 815 cells, $4T1_M$ = 1010 cells). Cells are colored by cell type. **(B)** Differential abundance plot of monocytic cells (dotted box in (A)), highlighting two significantly enriched, cellular neighborhoods (dotted lines). The top 10, most significant marker genes of each neighborhood are listed (FDR<0.001, log_2 fold-change >1.5). Genes underlined are classical markers of M1 (responders) and M2 (non-responders) macrophages. **(C-I)** scRNA-seq analysis of the neutrophil subpopulation displaying **(C)** a UMAP plot of 2886 GR1$^+$ neutrophils, highlighting two significant, differentially abundant cellular neighborhoods found in $4T1_P$ and $4T1_M$ tumors. Data is identical to that shown in Fig 3. *Significance of each neighborhood was determined via a randomized, permutations test;* **(D)** Raw, RNA-velocity flow-field vectors projected back onto the UMAP; **(E-I)** Binned, normalized expression of known granulocytic progenitor genes. *Data was imputed for visual clarity.*

FIGURE S3

A

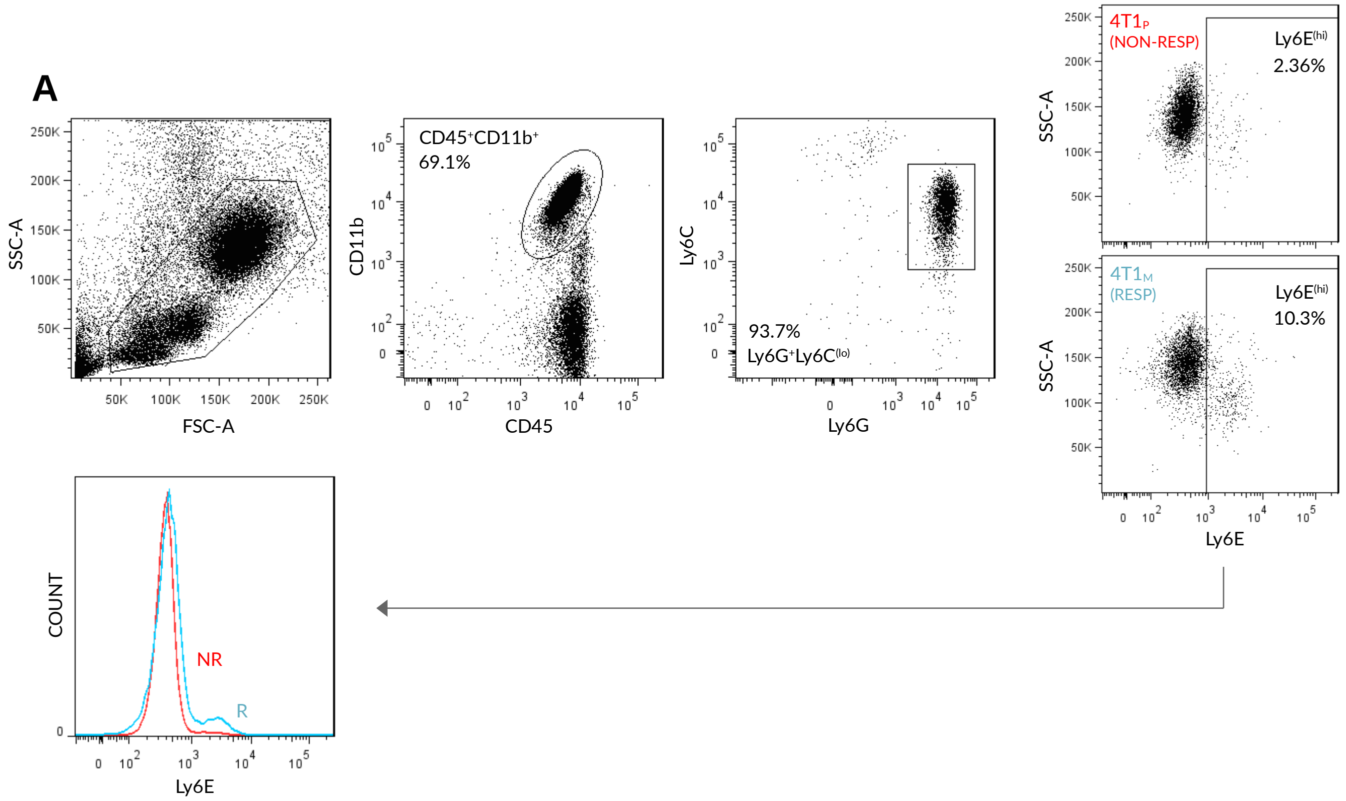
SSC-A
FSC-A
CD45+CD11b+
69.1%
CD11b
CD45
Ly6C
Ly6G
93.7%
Ly6G+Ly6C(lo)
4T1P
(NON-RESP)
Ly6E(hi)
2.36%
4T1M
(RESP)
Ly6E(hi)
10.3%
Ly6E
COUNT
NR
R
Ly6E

B

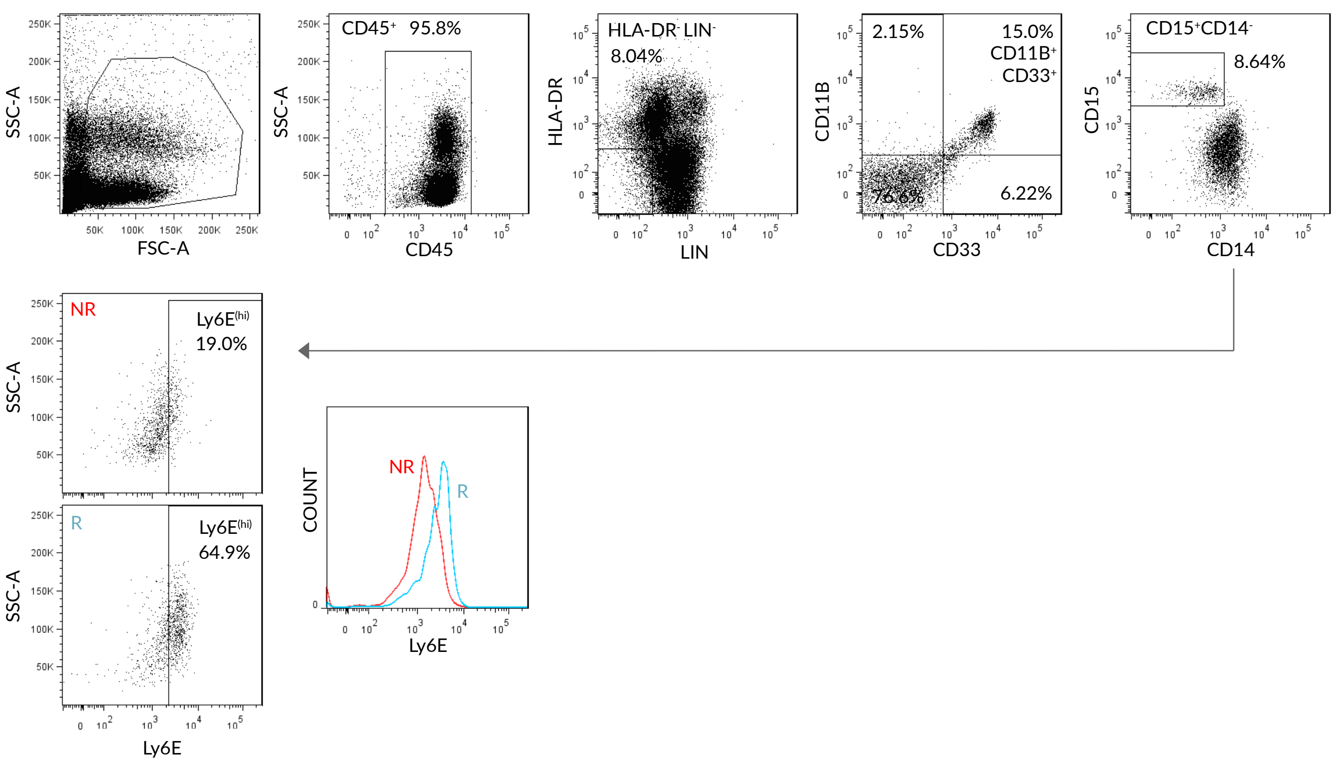
SSC-A
FSC-A
CD45+ 95.8%
CD45
HLA-DR- LIN-
8.04%
HLA-DR
LIN
2.15%
15.0%
CD11B+
CD33+
76.6%
6.22%
CD11B
CD33
CD15+CD14-
8.64%
CD15
CD14
NR
Ly6E(hi)
19.0%
R
Ly6E(hi)
64.9%
Ly6E
COUNT
NR
R
Ly6E

Figure S3. Gating strategy of Ly6E$^{(hi)}$ neutrophils in mouse and human, related to Figures 3 and 6. **(A)** Representative flow cytometry plots taken from the blood of BALB/c mice bearing parental 4T1$_{P}$ (NR) or mutagenized 4T1$_{M}$ (R) breast carcinoma. The analysis was performed on whole blood cells gated for CD45^{+}/CD11b^{+}, Ly6C$^{(lo)}$/Ly6G^{+}, and Ly6E$^{(hi)}$. Notably, the percentage of Ly6E$^{(hi)}$ gated cells is higher in mice bearing 4T1$_{M}$ tumors. **(B)** Representative flow cytometry plots taken from peripheral blood mononuclear cells (PBMCs) of patients with NSCLC at baseline. Data is stratified into non-responders (NR) and responders (R) based on RECIST criteria at 3 and/or 6 months. The analysis was performed on PBMCs gated for CD45^{+}, Lin^{-}/HLA-DR^{-}, CD14^{-}/CD15^{+}, and Ly6E$^{(hi)}$. Notably, the percentage of Ly6E$^{(hi)}$ gated cells is higher in responding patients.

FIGURE S4

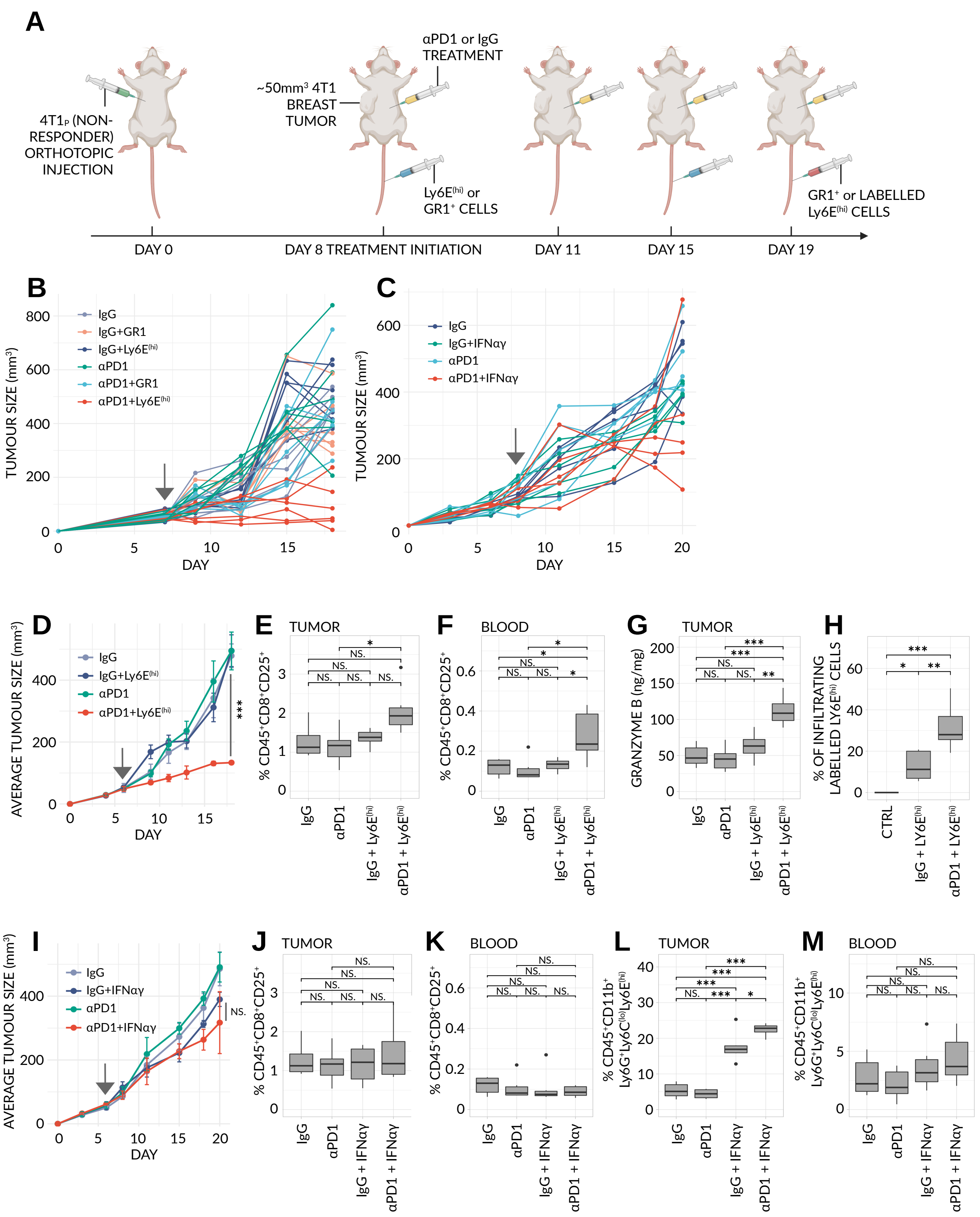
A
4T1P (NON-RESPONDER) ORTHOTOPIC INJECTION
~50mm³ 4T1 BREAST TUMOR
αPD1 or IgG TREATMENT
Ly6E(hi) or GR1+ CELLS
GR1+ or LABELLED Ly6E(hi) CELLS
DAY 0
DAY 8 TREATMENT INITIATION
DAY 11
DAY 15
DAY 19
B
TUMOUR SIZE (mm³)
DAY
IgG
IgG+GR1
IgG+Ly6E(hi)
αPD1
αPD1+GR1
αPD1+Ly6E(hi)
C
IgG
IgG+IFNαγ
αPD1
αPD1+IFNαγ
D
AVERAGE TUMOUR SIZE (mm³)
E
TUMOR
% CD45+CD8+CD25+
F
BLOOD
G
GRANZYME B (ng/mg)
H
% OF INFILTRATING LABELLED LY6E(hi) CELLS
CTRL
IgG + LY6E(hi)
αPD1 + LY6E(hi)
IgG
αPD1
IgG + Ly6E(hi)
αPD1 + Ly6E(hi)
I
J
K
L
% CD45+CD11b+ Ly6G+Ly6C(lo)Ly6E(hi)
M
IgG + IFNαγ
αPD1 + IFNαγ
NS.

Figure S4. Ly6E$^{(hi)}$ neutrophil cell therapy, but not systemic IFNα/γ treatment, induces immunotherapy response, related to Figure 4. **(A)** Schematic overview of the adoptive transfer protocol (see Methods for details). In brief, non-responding 4T1 tumors ($4T1_P$) were orthotopically injected into BALB/c mice. Treatment with either a monotherapy (control IgG or αPD1) or a combined therapy, with GR1$^+$ or Ly6E$^{(hi)}$ neutrophils, was initiated on Day 8 at an average tumor size of ~50mm^3. Additional doses were given throughout the time-course as marked. On Day 19, Ly6E$^{(hi)}$ neutrophils were labeled with a Live Cell Labeling allophycocyanin (APC) kit to track migration of these cells into the TME (see: Fig S4H) and as written in Methods. **(B-C)** Raw, individual tumor growth profiles of mice bearing parental, non-responsive $4T1_P$ breast tumors treated with either a monotherapy (control IgG or αPD1) or a combined therapy, with GR1$^+$ or Ly6E$^{(hi)}$ neutrophils (n=5 mice/group) **(B)**; or systemic IFNα/γ (n=6 mice/group) **(C)**; as specified. Treatment was initiated at a tumor size of ~50mm^3 (arrow) in all cases. **(D-H)** Ly6E$^{(hi)}$ neutrophils, generated *in-vitro,* were adoptively transferred into mice bearing parental, non-responsive $4T1_P$ breast tumors, with or without αPD1 or IgG control therapy. Data is a biological repeat of the experiment shown in Fig 4. **(D)** Averaged tumor growth profiles for all conditions (n=5 mice/group). Frequencies of activated, CD25$^+$CD8$^+$ T cells in the TME **(E)**; and in the blood **(F)** at end point; as determined by flow cytometry. Granzyme B concentrations in tumor lysates, as measured by ELISA **(G)** and frequency of Ly6E$^{(hi)}$ neutrophils that have infiltrated into the tumor, as tracked by APC cell labeling (see: Fig. S4A) **(H)**. **(I-M)** In a separate experiment, mice bearing $4T1_P$ tumors were systemically administered IFNα/γ with or without αPD1 or IgG control therapy (n=6 mice/group). **(I)** Averaged tumor growth profiles for all conditions (see: Fig. S4C for raw data). Frequencies of: activated, CD25$^+$CD8$^+$ T cells and Ly6E$^{(hi)}$ neutrophils in the TME at end-point **(J, L)** and in the blood **(K, M)** were analyzed by flow cytometry. *Significance was assessed by means of a two-sample KS-test (growth) and a one-way ANOVA and Tukey's post-hoc HSD test (flow) (NS, p>0.01; * p<0.01; **, p<0.001; ***, p<0.0001).*

FIGURE S5

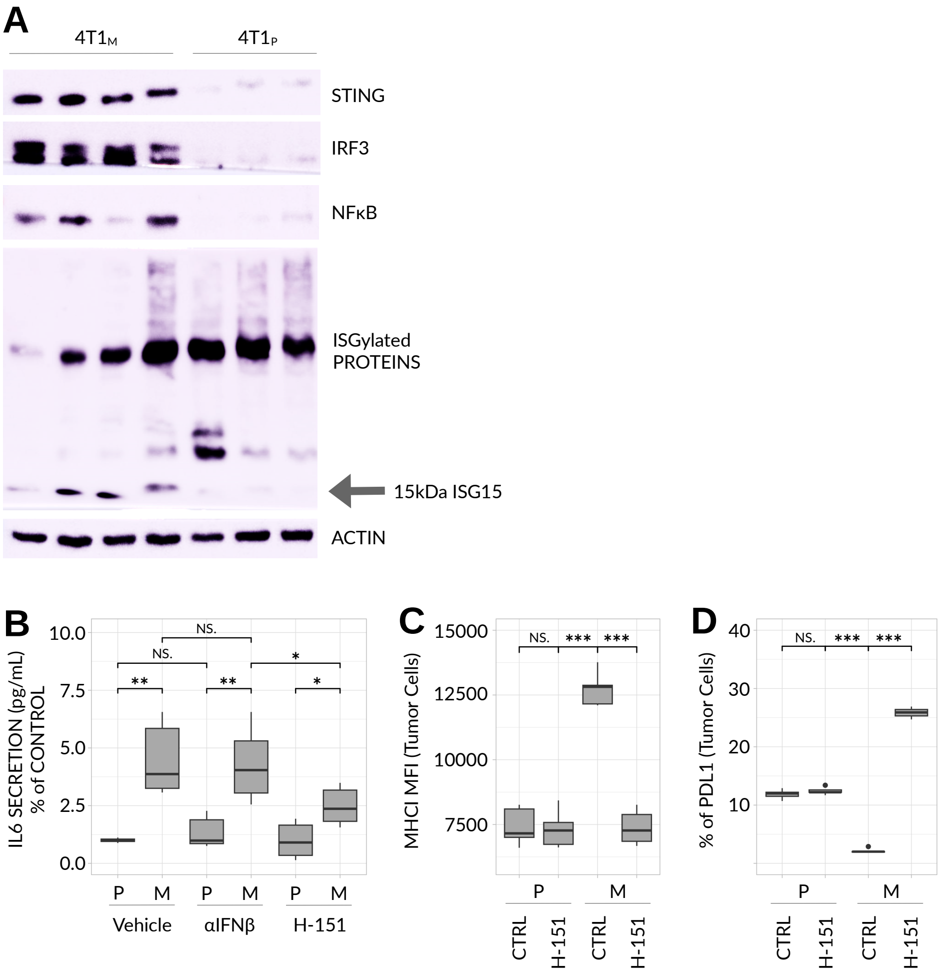

A
4T1M
4T1P
STING
IRF3
NFκB
ISGylated PROTEINS
15kDa ISG15
ACTIN
B
IL6 SECRETION (pg/mL) % of CONTROL
10.0
7.5
5.0
2.5
0.0
NS.
NS.
**
**
*
*
P
M
P
M
P
M
Vehicle
αIFNβ
H-151
C
MHCI MFI (Tumor Cells)
15000
12500
10000
7500
NS.
***
***
P
M
CTRL
H-151
CTRL
H-151
D
% of PDL1 (Tumor Cells)
40
30
20
10
NS.
***
***
P
M
CTRL
H-151
CTRL
H-151

Figure S5. STING activity is a hallmark of 4T1$_M$ tumors, related to Figure 5. **(A)** Western blot of STING-pathway related proteins in 4T1$_P$ and 4T1$_M$ tumor lysates (n=3-4 biological repeats/group). **(B)** Levels of secreted IL6, as assayed by ELISA from the conditioned media of cultured 4T1$_P$ (P) and 4T1$_M$ (M) cell lines, alone or in the presence of αIFNβ or the STING-inhibitor H151 (n=6 biological repeats/group). **(C-D)** Levels of cell-surface MHCI **(C)**; or PDL1 **(D)** on cultured 4T1$_P$ (P) and 4T1$_M$ (M) cell lines as determined by flow cytometry with or without the STING-inhibitor H151 (n=3-5 biological repeats/group). *In (B-D), significance was assessed by means of a one-way ANOVA and Tukey's post-hoc HSD test (NS, p>0.01; *, p<0.01; **, p<0.001; ***, p<0.0001).*

FIGURE S6

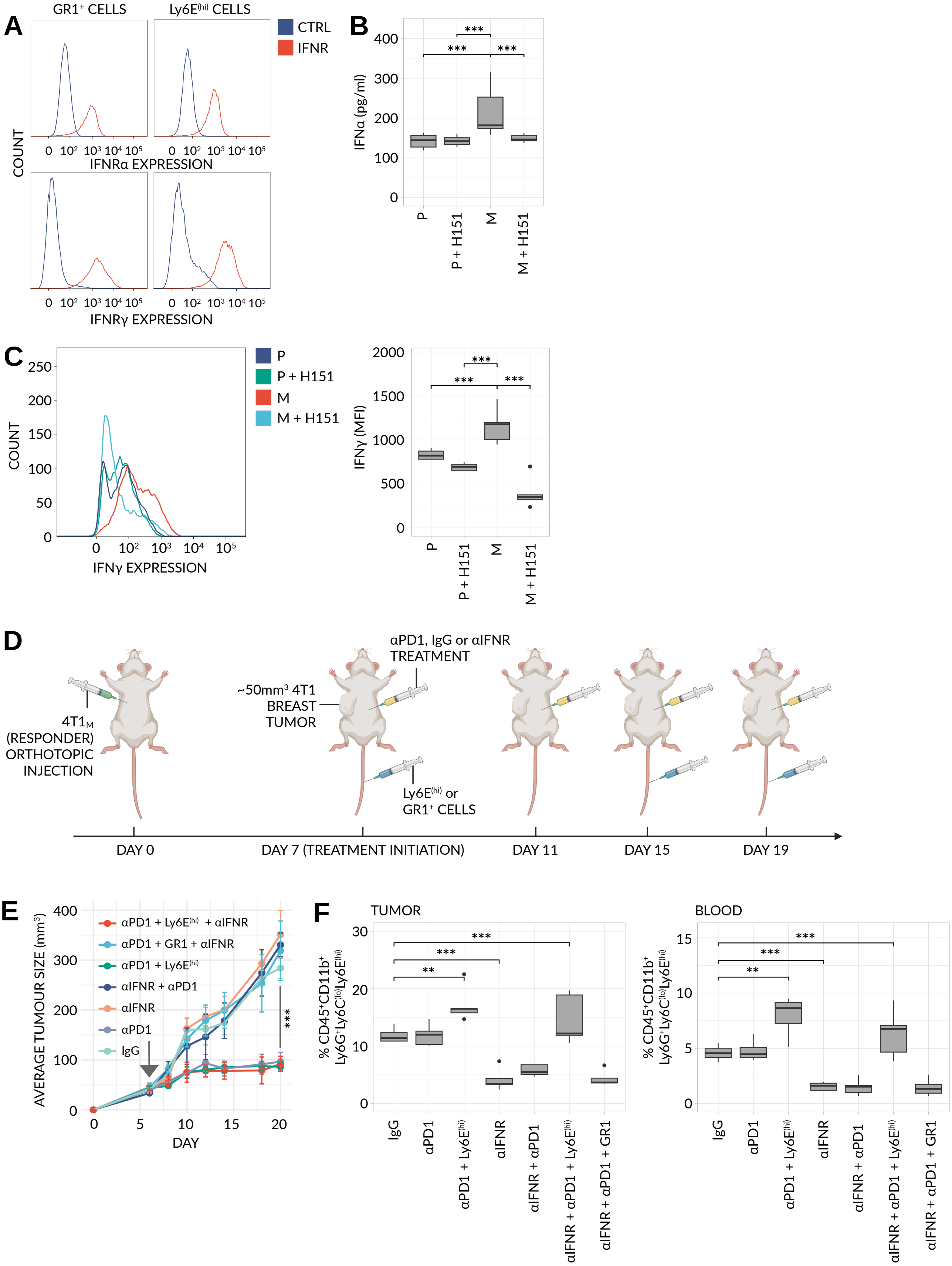
A
GR1+ CELLS
Ly6E(hi) CELLS
CTRL
IFNR
COUNT
IFNRα EXPRESSION
IFNRγ EXPRESSION
B
IFNα (pg/ml)
P
P + H151
M
M + H151
C
COUNT
IFNγ EXPRESSION
IFNγ (MFI)
D
4T1M (RESPONDER) ORTHOTOPIC INJECTION
~50mm3 4T1 BREAST TUMOR
αPD1, IgG or αIFNR TREATMENT
Ly6E(hi) or GR1+ CELLS
DAY 0
DAY 7 (TREATMENT INITIATION)
DAY 11
DAY 15
DAY 19
E
AVERAGE TUMOUR SIZE (mm3)
DAY
αPD1 + Ly6E(hi) + αIFNR
αPD1 + GR1 + αIFNR
αPD1 + Ly6E(hi)
αIFNR + αPD1
αIFNR
αPD1
IgG
F
TUMOR
BLOOD
% CD45+CD11b+ Ly6G+Ly6C(lo)Ly6E(hi)
IgG
αPD1
αPD1 + Ly6E(hi)
αIFNR
αIFNR + αPD1
αIFNR + αPD1 + Ly6E(hi)
αIFNR + αPD1 + GR1

Figure S6. Ly6E$^{(hi)}$ neutrophils rescue anti-PD1 responsiveness in 4T1 tumors made resistant through IFNR blockade, related to Figure 5. **(A)** Expression of IFNα/γ and their cognate receptors (IFNR-α/γ) within tumor and immune cells was examined using spleens from BALB/c mice, bearing $4T1_P$ tumors, that were harvested and prepared as single-cell suspensions (n=5 biological repeats/group). Expression levels of INFRα and IFNRγ were subsequently analyzed on the surface of $GR1^+$ and Ly6E$^{(hi)}$ neutrophils using flow cytometry. Histogram plots, depicting expression levels, are shown. **(B-C)** Non-responsive $4T1_P$ (P) and responsive $4T1_M$ (M) cancer cells were cultured in the presence or absence of the STING inhibitor, H151 (see: Methods) (n=8 biological repeats/group). The cells were prepared as lysates, and the levels of IFNα were quantified using ELISA **(B)**, while IFNγ levels were assessed by means of flow cytometry (n=5 biological repeats/group) **(C)**. Of note, IFNγ levels remained below the detection level of ELISA – necessitating the use of flow cytometry. A representative histogram and summarizing boxplots displaying the mean fluorescent intensity (MFI) of IFNγ are shown. **(D)** Schematic overview of the rescue experiment, whereby Ly6E$^{(hi)}$ neutrophils are adoptively transferred into mice bearing $4T1_M$ tumors made resistant to anti-PD1. In brief, $4T1_M$ tumors were orthotopically injected into BALB/c mice and treatment with monotherapy (control IgG, αPD1, αIFNRα/γ) or combined therapy (αPD1, αIFNRα/γ) with or without adoptively transferred $GR1^+$ or Ly6E$^{(hi)}$ neutrophils, was initiated on Day 7 at an average tumor size of ~50mm^3. Additional doses were given throughout the time-course as marked. On day 20, mice were sacrificed, and tumors and blood were analyzed for Ly6E$^{(hi)}$ neutrophils. **(E)** Averaged tumor growth profiles for the mice described above are shown (n=5 mice/group). **(F)** Tumors were harvested and prepared as single-cell suspensions and blood was drawn for the analysis of Ly6E$^{(hi)}$ neutrophil levels using flow cytometry. *Significance was assessed by means of a two-sample KS-test (growth) and one-way ANOVA and Tukey's post-hoc HSD test (flow and ELISA) (**, $p < 0.001$; ***, $p<0.0001$).*

FIGURE S7

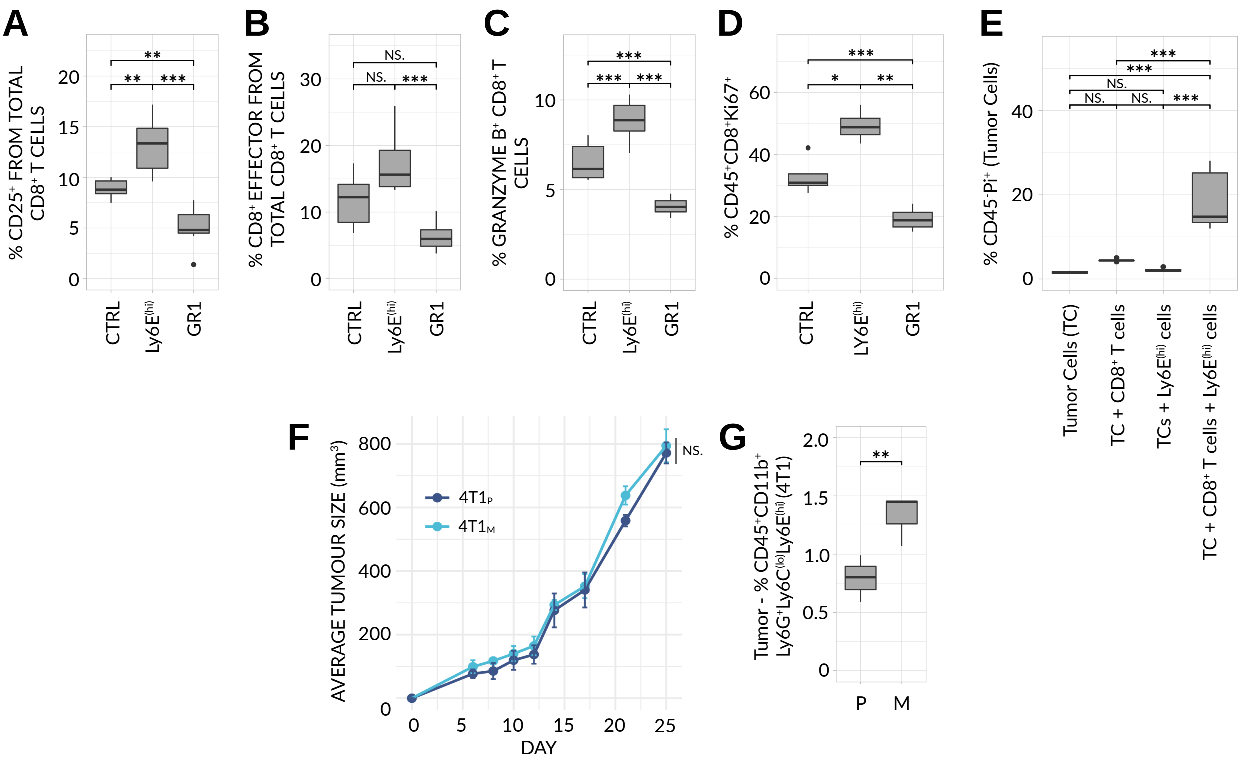

A
% CD25+ FROM TOTAL CD8+ T CELLS
CTRL
Ly6E(hi)
GR1
B
% CD8+ EFFECTOR FROM TOTAL CD8+ T CELLS
C
% GRANZYME B+ CD8+ T CELLS
D
% CD45+CD8+Ki67+
LY6E(hi)
E
% CD45-PI+ (Tumor Cells)
Tumor Cells (TC)
TC + CD8+ T cells
TCs + Ly6E(hi) cells
TC + CD8+ T cells + Ly6E(hi) cells
F
AVERAGE TUMOUR SIZE (mm3)
DAY
4T1P
4T1M
NS.
G
Tumor - % CD45+CD11b+ Ly6G+Ly6C(lo)Ly6E(hi) (4T1)
P
M

Figure S7. Ly6E$^{(hi)}$ neutrophils contribute to and act upstream of anti-tumor T cell activity, related to Figure 5. **(A-D)** $CD8^+$ T cells, obtained from the spleens of $4T1_P$ tumor bearing mice, were cultured *in-vitro* either alone (CTRL) or with conditioned media derived from $GR1^+$ cells or IFNαγ-induced, Ly6E$^{(hi)}$ (Ly6E) neutrophils (n=8 biological repeats/group). Frequencies or levels of: activated, $CD8^+CD25^+$ T cells **(A)**; effector $CD8^+$ T cells **(B)**; Granzyme B^+ $CD8^+$ T cells **(C)**; and proliferating $CD8^+$ T cells **(D)**, were determined by flow cytometry. **(E)** In a separate experiment, $4T1_P$ cells were cultured in the presence of $CD8^+$ T cells or Ly6E$^{(hi)}$ neutrophils for 24 hours in a ratio of 1:10:10. T cell killing efficacy was analyzed by flow cytometry using PI to detect dead tumor cells (n=5 biological repeats/group). **(F-G)** $4T1_P$ and $4T1_M$ cells were implanted in 8 week old SCID mice (n=3 mice/group). **(F)** Averaged tumor growth curves for both tumor types. **(G)** Frequencies of Ly6E$^{(hi)}$ neutrophils as assessed by flow cytometry in blood profiles as tumors reached a size of ~50mm^3. *Significance was assessed by means of a two-sample KS-test (growth) and a one-way ANOVA and Tukey's post-hoc HSD test (flow) (NS, $p>0.01$; * $p<0.01$; **, $p<0.001$; ***, $p<0.0001$).*

FIGURE S8

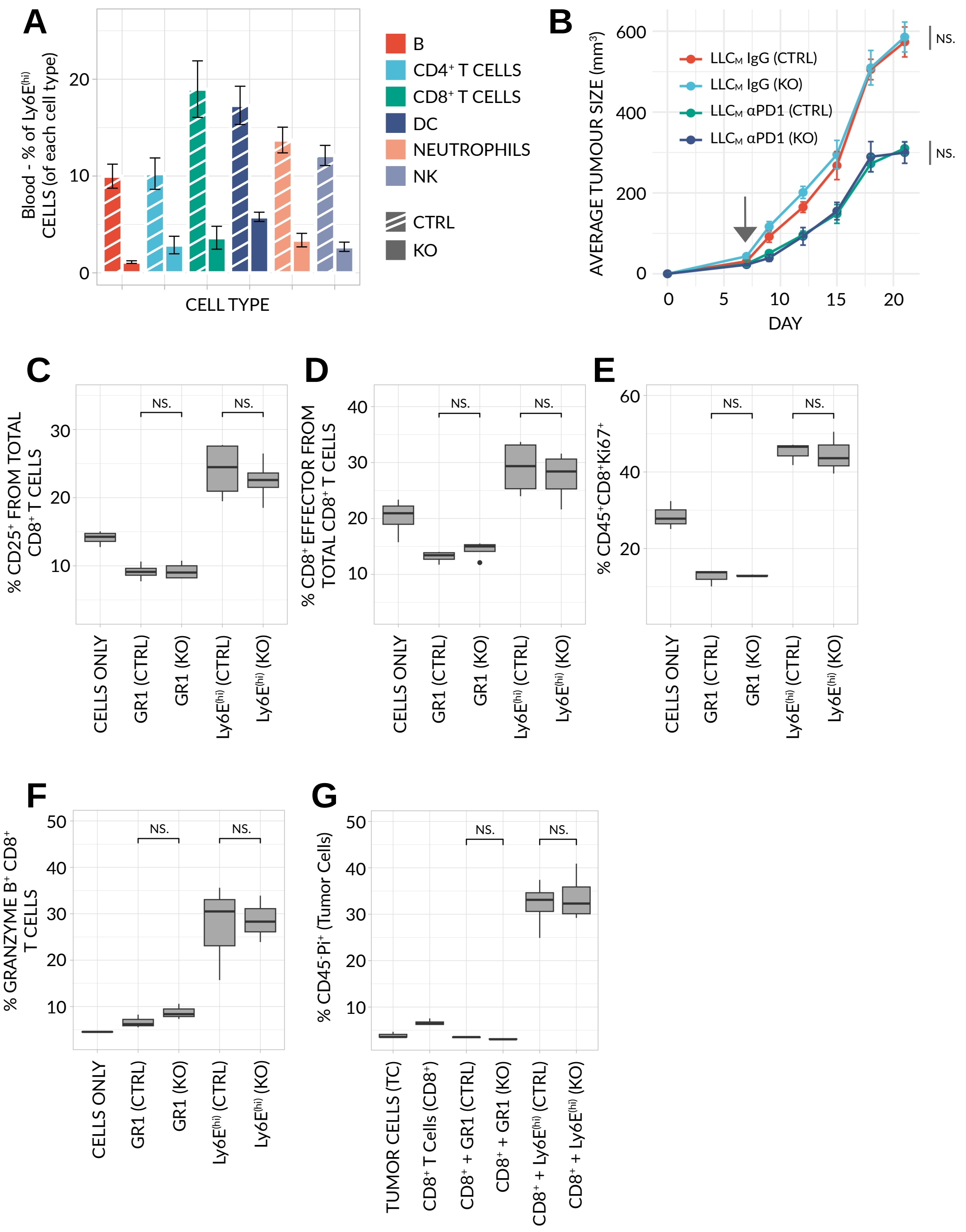
A
Blood - % of Ly6E(hi) CELLS (of each cell type)
CELL TYPE
B
CD4+ T CELLS
CD8+ T CELLS
DC
NEUTROPHILS
NK
CTRL
KO
B
AVERAGE TUMOUR SIZE (mm3)
DAY
LLCM IgG (CTRL)
LLCM IgG (KO)
LLCM αPD1 (CTRL)
LLCM αPD1 (KO)
NS.
C
% CD25+ FROM TOTAL CD8+ T CELLS
D
% CD8+ EFFECTOR FROM TOTAL CD8+ T CELLS
E
% CD45+CD8+Ki67+
CELLS ONLY
GR1 (CTRL)
GR1 (KO)
Ly6E(hi) (CTRL)
Ly6E(hi) (KO)
F
% GRANZYME B+ CD8+ T CELLS
G
% CD45-PI+ (Tumor Cells)
TUMOR CELLS (TC)
CD8+ T Cells (CD8+)
CD8+ + GR1 (CTRL)
CD8+ + GR1 (KO)
CD8+ + Ly6E(hi) (CTRL)
CD8+ + Ly6E(hi) (KO)

Figure S8. Ly6E itself has no functional role in immunotherapy response, related to Figure 5. **(A-G)** Lin^- bone-marrow progenitor cells, isolated from C57BL/6 strains over-expressing Cas9, were transfected with lentivirus carrying a single guide RNA GFP-CRISPR-Cas9 construct targeting the Ly6E locus. A transfection rate of ~80%, as measured by GFP expression, was observed. Transfected cells were subsequently implanted into irradiated, otherwise WT C57BL/6 mice (C57BL/6Ly6EKO). **(A)** Frequencies of $Ly6E^{(hi)}$ cells across all major immune subtypes were determined by flow cytometry (n=5 mice/group). CTRL = single guide RNA without the Ly6E-targeting. **(B)** Averaged tumor growth profiles for C57BL/6CTRL or C57BL/6Ly6EKO mice implanted with mutagenized Lewis-lung carcinoma (LLC_M), and treated with αPD1 or control IgG antibodies (n=6-7 mice/group). **(C-G)** $CD8^+$ T cells, obtained from the spleens of LLC tumor bearing mice, were cultured *in-vitro* either alone or with conditioned media derived from $GR1^+$ cells or IFNαγ-induced, $Ly6E^{(hi)}$ neutrophils (n=4-5 biological repeats/group). Frequencies or levels of: activated, $CD8^+CD25^+$ T cells **(C)**; effector $CD8^+$ T cells **(D)**; proliferating $CD8^+$ T cells **(E)**; and Granzyme B^+ $CD8^+$ T cells **(F)**, were determined by flow cytometry. **(G)** In a separate experiment, $4T1_P$ cells were cultured in the presence of $CD8^+$ T cells or $Ly6E^{(hi)}$ neutrophils for 24 hours in a ratio of 1:10:10. T cell killing efficacy was analyzed by flow cytometry using PI to detect dead tumor cells (n=4 biological repeats/group). *Significance was assessed by means of a two-sample KS-test (growth) and one-way ANOVA and Tukey's post-hoc HSD test (flow and ELISA) (NS, p>0.01).*

FIGURE S9

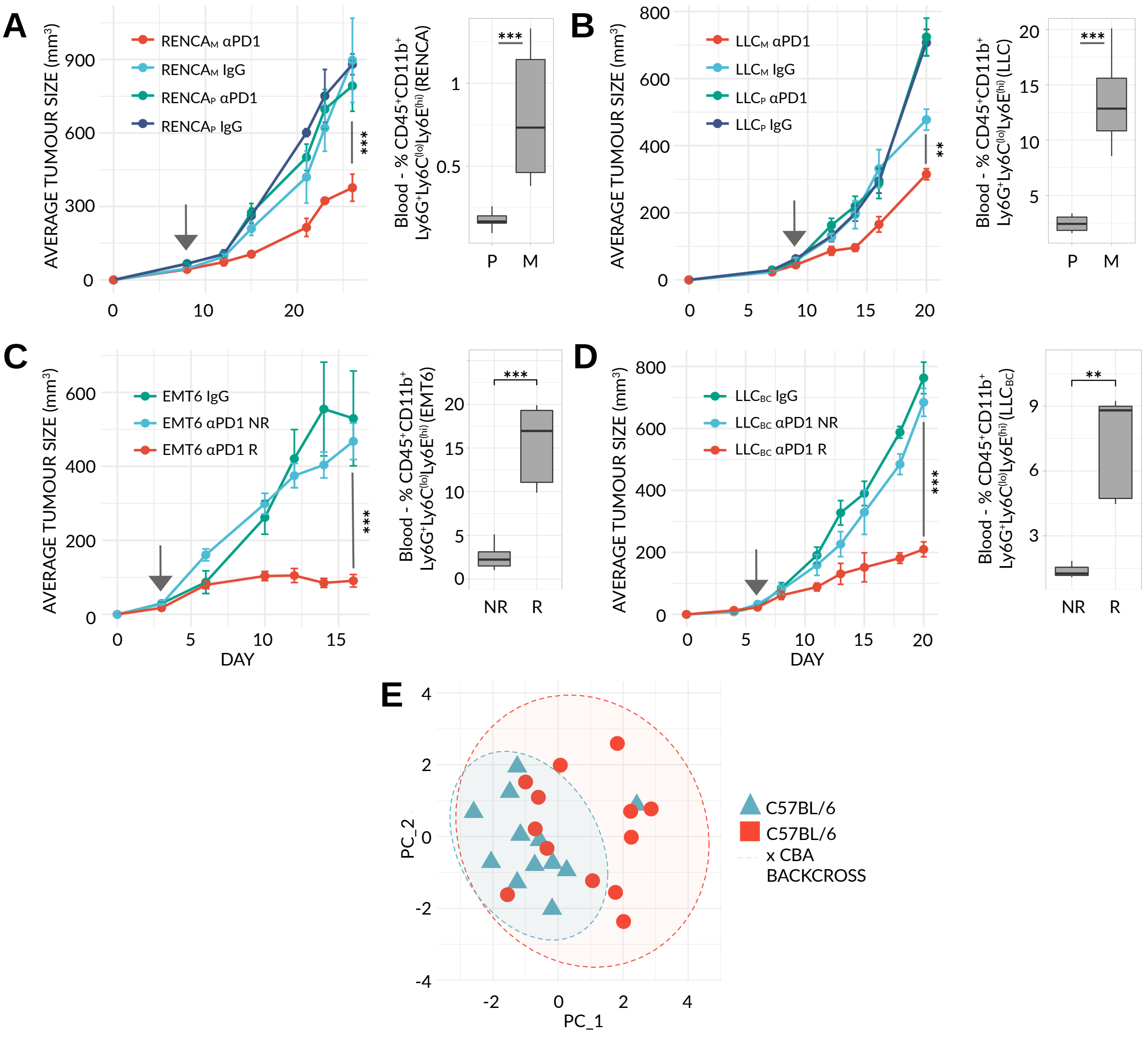

A
RENCAM αPD1
RENCAM IgG
RENCAP αPD1
RENCAP IgG
AVERAGE TUMOUR SIZE (mm3)
Blood - % CD45+CD11b+ Ly6G+Ly6C(lo)Ly6E(hi) (RENCA)
P
M
B
LLCM αPD1
LLCM IgG
LLCP αPD1
LLCP IgG
Blood - % CD45+CD11b+ Ly6G+Ly6C(lo)Ly6E(hi) (LLC)
C
EMT6 IgG
EMT6 αPD1 NR
EMT6 αPD1 R
Blood - % CD45+CD11b+ Ly6G+Ly6C(lo)Ly6E(hi) (EMT6)
NR
R
DAY
D
LLCBC IgG
LLCBC αPD1 NR
LLCBC αPD1 R
Blood - % CD45+CD11b+ Ly6G+Ly6C(lo)Ly6E(hi) (LLCBC)
E
PC_1
PC_2
C57BL/6
C57BL/6 x CBA BACKCROSS

Figure S9. Ly6E$^{(hi)}$ neutrophils mark immunotherapy response in diverse mouse tumor models, related to Figure 5. **(A-D)** Averaged tumor growth profiles for: BALB/c mice implanted with parental ($RENCA_P$) or mutagenized ($RENCA_M$) renal cell carcinoma (n=6 mice/group) **(A)**; C57BL/6 mice implanted with parental (LLC_P) or mutagenized (LLC_M) Lewis lung carcinoma (n=6 mice/group) **(B)**; BALB/C mice implanted with spontaneously responding EMT6 breast cancer (IgG n=5, αPD1 n=45) **(C)** and C57BL/6 x CBA backcrossed mice implanted with parental Lewis lung cancer (LLC) (IgG n=5, αPD1 n=15) **(D)**. In all cases, the frequencies of Ly6E$^{(hi)}$ neutrophils in the blood of each model are shown (right) as determined by flow cytometry. In the case of (C-D) unsupervised, hierarchical clustering and pairwise comparison to respective control, IgG-treated mice was utilized to segregate mice into non-responding (NR) and responding (R) groups. In all cases, treatment was initiated at a tumor size of ~50mm^3 (arrow). *Significance was assessed by means of two-sample KS-test (growth) and a Mann-Whitney test (flow) (**, p<0.001; ***, p<0.0001).* **(E)** Principal component analysis, based on the frequencies of all major immune cell types (NK, B, CD8/4$^+$ T cells, monocytes, granulocytes) in the blood of non-tumor bearing C57BL/6 (n=13) and C57BL/6 x CBA backcross (n=13) mice, as determined by flow cytometry and expressed as a % of CD45$^+$ cells. *95% confidence intervals are shown (ellipses).*

FIGURE S10

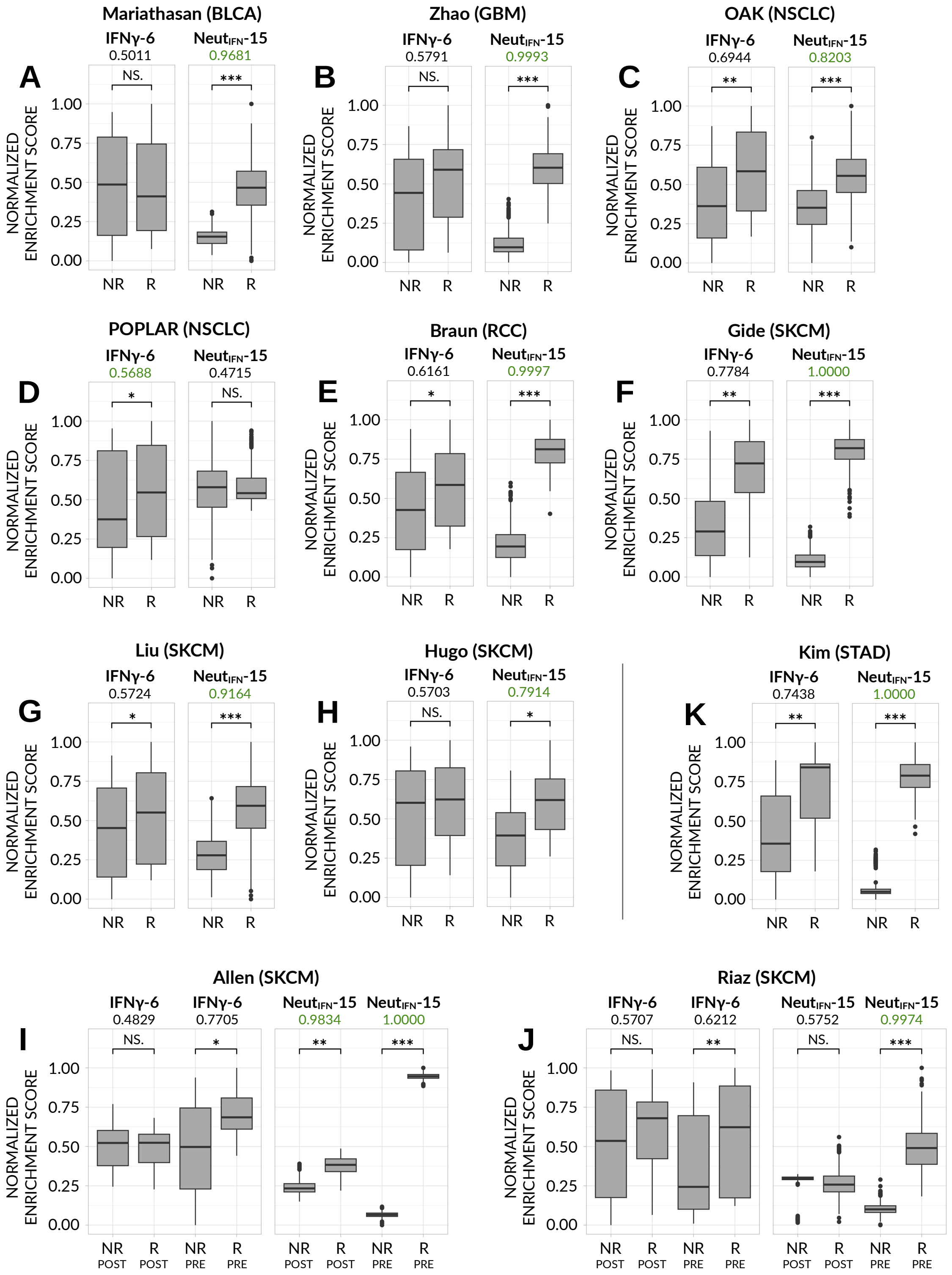

Mariathasan (BLCA)
IFNγ-6 0.5011
NeutIFN-15 0.9681
Zhao (GBM)
IFNγ-6 0.5791
NeutIFN-15 0.9993
OAK (NSCLC)
IFNγ-6 0.6944
NeutIFN-15 0.8203
POPLAR (NSCLC)
IFNγ-6 0.5688
NeutIFN-15 0.4715
Braun (RCC)
IFNγ-6 0.6161
NeutIFN-15 0.9997
Gide (SKCM)
IFNγ-6 0.7784
NeutIFN-15 1.0000
Liu (SKCM)
IFNγ-6 0.5724
NeutIFN-15 0.9164
Hugo (SKCM)
IFNγ-6 0.5703
NeutIFN-15 0.7914
Kim (STAD)
IFNγ-6 0.7438
NeutIFN-15 1.0000
Allen (SKCM)
IFNγ-6 0.4829
IFNγ-6 0.7705
NeutIFN-15 0.9834
NeutIFN-15 1.0000
Riaz (SKCM)
IFNγ-6 0.5707
IFNγ-6 0.6212
NeutIFN-15 0.5752
NeutIFN-15 0.9974
NORMALIZED ENRICHMENT SCORE
NR R
POST PRE

Figure S10. A Ly6E$^{(hi)}$ neutrophil-derived gene signature significantly outperforms the previously published IFNγ-6 signature, related to Figure 7. Boxplots depicting raw enrichment scores obtained from the IFNγ-6 signature (Left) and the Ly6E$^{(hi)}$ Neut$_{IFN}$-15 signature (Right). AUC values are shown for each dataset and signature, denoting the ability of the scores to stratify responders and non-responders. The highest performing signature within each dataset is denoted in green. *Significance was assessed by means of a one-way Mann-Whitney test (NS, p>0.01; *, p<0.01; **, p<0.001, ***, p<0.0001).*
