# Supplementary material for: Interferon-stimulated neutrophils as a predictor of immunotherapy response
Source: Cancer Cell. 2024 Feb 12;42(2):253–265.e12. doi: 10.1016/j.ccell.2023.12.005 (PMC10864002; doi:10.1016/j.ccell.2023.12.005)
Supplement: Document S2. Article plus supplemental information [file mmc4.pdf]

Cancer Cell

Article

Interferon-stimulated neutrophils as a predictor of immunotherapy response

Graphical abstract

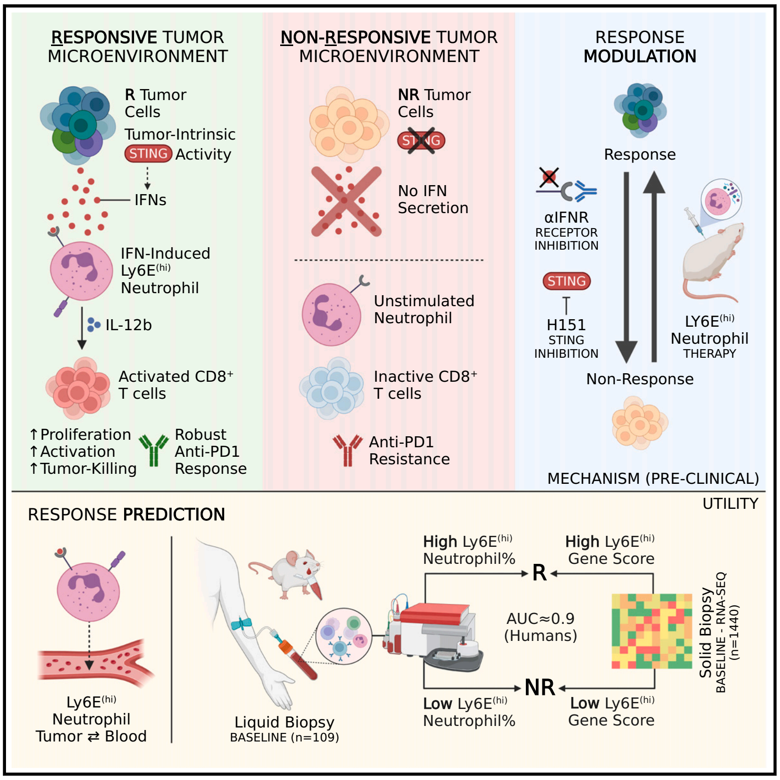

Highlights

- Ly6E^{hi} neutrophils are interferon-stimulated cells present in both mouse and human
- Ly6E^{hi} neutrophils accurately predict immunotherapy outcomes in different cancer types
- Activation of the STING pathway in tumors accounts for Ly6E^{hi} neutrophil enrichment
- Ly6E^{hi} neutrophils sensitize otherwise resistant tumors to immunotherapy in mice

Authors

Madeleine Benguigui, Tim J. Cooper, Prajakta Kalkar, ..., Amiram Ariel, Shai S. Shen-Orr, Yuval Shaked

Correspondence

timcooper@technion.ac.il (T.J.C.), yshaked@technion.ac.il (Y.S.)

In brief

A lack of reliable, highly predictive biomarkers remains a major obstacle in immuno-oncology. In this study, Benguigui et al. discover a promising new biomarker: interferon-stimulated, Ly6E^{hi} neutrophils—whose frequency in the blood of both mice and patients strongly correlates with immunotherapy outcomes across cancer types.

Benguigui et al., 2024, Cancer Cell 42, 253–265
February 12, 2024 © 2023 The Authors. Published by Elsevier Inc.
https://doi.org/10.1016/j.ccell.2023.12.005

CellPress

Cancer Cell

CellPress
OPEN ACCESS

Article

Interferon-stimulated neutrophils as a predictor of immunotherapy response

Madeleine Benguigui,[1,2,12] Tim J. Cooper,[1,2,3,12,*] Prajakta Kalkar,[4] Sagie Schif-Zuck,[4] Ruth Halaban,[5] Antonella Bacchiocchi,[5] Iris Kamer,[6] Abhilash Deo,[1,2] Bar Manobla,[1,2] Rotem Menachem,[1,2] Jozafina Haj-Shomaly,[1,2] Avital Vorontsova,[1,2] Ziv Raviv,[1,2] Chen Buxbaum,[1,2] Petros Christopoulos,[7,8] Jair Bar,[6,9] Michal Lotem,[10] Mario Sznol,[11] Amiram Ariel,[4] Shai S. Shen-Orr,[2,3] and Yuval Shaked[1,2,13,*]

[1]Cell Biology and Cancer Science, Rappaport Faculty of Medicine, Technion – Israel Institute of Technology, Haifa, Israel
[2]Rappaport Technion Integrated Cancer Center, Technion – Israel Institute of Technology, Haifa, Israel
[3]Department of Immunology, Rappaport Faculty of Medicine, Technion – Israel Institute of Technology, Haifa, Israel
[4]Department of Human Biology, the Faculty of Natural Sciences, University of Haifa, Haifa, Israel
[5]Department of Dermatology, Yale Cancer Center, Yale University School of Medicine, New Haven, CT, USA
[6]Institute of Oncology, Sheba Medical Center, Tel Hashomer, Ramat Gan, Israel
[7]Department of Thoracic Oncology, Thoraxklinik and National Center for Tumor Diseases (NCT) at Heidelberg University Hospital, 69126 Heidelberg, Germany
[8]Translational Lung Research Center Heidelberg, Member of the German Center for Lung Research (DZL), Heidelberg, Germany
[9]Faculty of Medicine, Tel-Aviv University, Tel-Aviv, Israel
[10]Department of Melanoma and Cancer Immunotherapy, Sharett Institute of Oncology, Hadassah Hebrew University Medical Center, Jerusalem, Israel
[11]Department of Medicine, Division of Medical Oncology, Yale University School of Medicine, New Haven, CT, USA
[12]These authors contributed equally
[13]Lead contact
*Correspondence: timcooper@technion.ac.il (T.J.C.), yshaked@technion.ac.il (Y.S.)
https://doi.org/10.1016/j.ccell.2023.12.005

SUMMARY

Despite the remarkable success of anti-cancer immunotherapy, its effectiveness remains confined to a subset of patients—emphasizing the importance of predictive biomarkers in clinical decision-making and further mechanistic understanding of treatment response. Current biomarkers, however, lack the power required to accurately stratify patients. Here, we identify interferon-stimulated, Ly6E^{hi} neutrophils as a blood-borne biomarker of anti-PD1 response in mice at baseline. Ly6E^{hi} neutrophils are induced by tumor-intrinsic activation of the STING (stimulator of interferon genes) signaling pathway and possess the ability to directly sensitize otherwise non-responsive tumors to anti-PD1 therapy, in part through IL12b-dependent activation of cytotoxic T cells. By translating our pre-clinical findings to a cohort of patients with non-small cell lung cancer and melanoma (n = 109), and to public data (n = 1440), we demonstrate the ability of Ly6E^{hi} neutrophils to predict immunotherapy response in humans with high accuracy (average AUC ≈ 0.9). Overall, our study identifies a functionally active biomarker for use in both mice and humans.

INTRODUCTION

In the era of personalized medicine, predictive biomarkers play a critical role in the clinical decision-making process by identifying optimized treatments tailored to each individual patient, and toward the particular characteristics of each tumor. In cancer, the integration of biomarkers into anti-cancer clinical trials significantly improves response rates.[1] Despite this, robust, predictive biomarkers for newer front-line cancer treatments remain underdeveloped or elusive. Immune checkpoint inhibitors (ICIs) (e.g., anti-PD1 and anti-CTLA4), a revolutionary form of immunotherapy, drastically improve 5-year survival rates in patients with advanced metastatic disease[2]; yet, only a fraction of patients exhibit durable response.[3] Pre-existing biomarkers for ICI outcome, including those used in clinical practice such as PDL1 immunohistochemistry (IHC), tumor mutational burden, or a variety of gene-signatures, are limited in their predictive power (AUC ≈ 0.6–0.75) and require, often inaccessible, tissue biopsies to profile.[4] Notably, these biomarkers are all tumor-intrinsic, yet, immunotherapy response depends on a complex, dynamic interplay between the tumor and the host.[5] Newer efforts to define biomarkers have therefore centered on varying aspects of the immune system, such as the rate of tumor-infiltrating T cells[6,7] or levels of myeloid-derived suppressor cells (MDSCs).[8] Nevertheless, accurate biomarkers for ICI outcome—applicable to multiple cancer types—remain a crucial but unfulfilled need in clinical oncology.

This is an open access article under the CC BY license (http://creativecommons.org/licenses/by/4.0/).

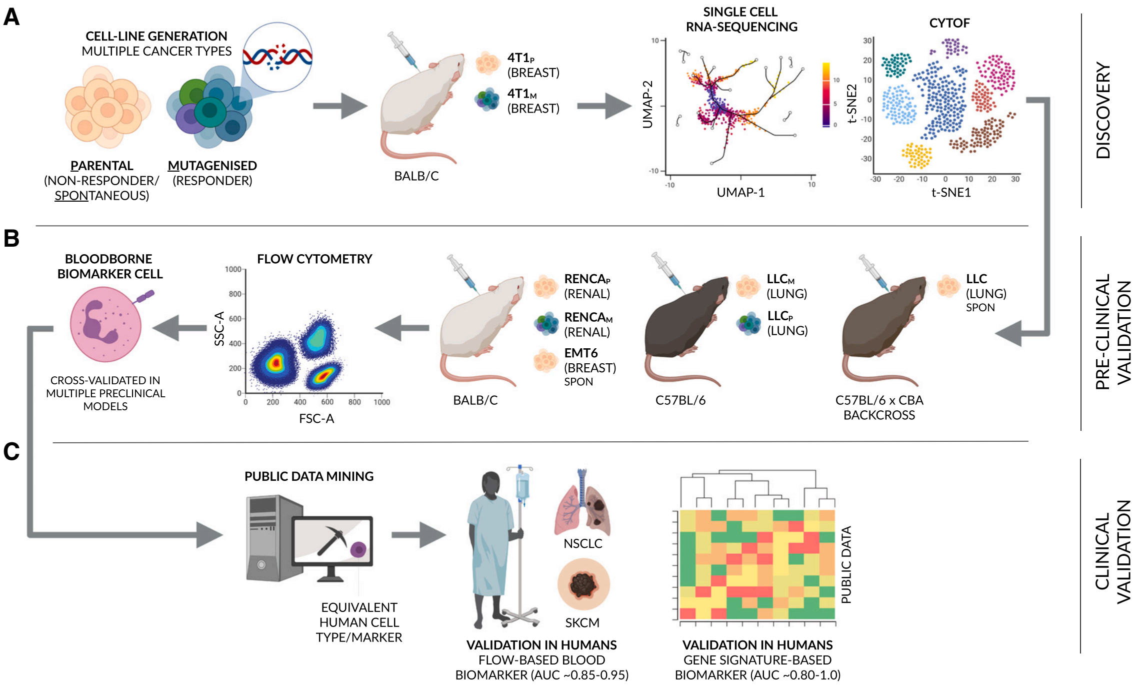

Figure 1. A multi-model approach to identify a clinically relevant biomarker for immunotherapy response

A schematic overview of the paper. (A and B) In brief, several mouse strains in combination with multiple cancer cell lines and clones were used to initially screen for and subsequently cross-validate a biomarker for immunotherapy response in mouse. (C) To clinically translate our findings, public data and data from a cohort of patients with non-small cell lung cancer (NSCLC) and skin cutaneous melanoma (SKCM) were used to assess the accuracy and utility of the identified biomarker in humans (see STAR Methods and introduction for additional, step-by-step details). Mouse strains: BALB/c, C57BL/6, and a C57BL/6 x CBA backcross. Cancer cell lines: 4T1 breast cancer, Lewis lung carcinoma (LLC), renal cell carcinoma (RENCA), and EMT6 breast cancer. P = parental cell line, M = mutagenized clone.

Biomarkers that integrate tumor- and host-dependent factors may, theoretically, outperform pre-existing markers. Thus, here, we combine single-cell RNA-sequencing (scRNA-seq) and a pre-clinical tumor model encompassing clones with intrinsically low and high immunogenicity, as generated through mutagenesis, to identify cellular states predictive of response that also reflect tumor-intrinsic patterning of host cells (Figure 1A). Specifically, we identify interferon-simulated, Ly6E^{hi} neutrophils—induced by tumor-intrinsic STING-signaling—as a tumor-infiltrating and blood-borne predictive biomarker for immunotherapy response in both mice and humans (AUC $\approx$ 0.9, humans) across a multitude of additional models and cancer types, respectively (Figures 1B and 1C). Moreover, we derive a 15-gene Ly6E^{hi} signature that accurately stratifies responders and non-responders in human, bulk RNA-seq data (average AUC > 0.9). Finally, we expand upon the functional characteristics of this neutrophil subtype, revealing its ability to directly sensitize otherwise non-responsive tumors to anti-PD1 in mice—in part through the modulation of cytotoxic CD8$^+$ T cell activity.

RESULTS

An interferon-stimulated subtype of neutrophil marks response to anti-PD1 in 4T1 breast cancer models

A prerequisite of biomarker discovery at baseline (i.e., pre-treatment) is the use of a stable and predictable model whose response outcome is known *a priori*—a requirement notably difficult and time-consuming to fulfill in humans.[9,10] In order to search for a biomarker to predict immunotherapy response, we therefore focused our initial efforts on pre-clinical models. Specifically, we generated 4T1 breast carcinoma cell lines, comprising a mutagenized clone ($4T1_M$), that is responsive to anti-PD1, derived from a non-responsive parental cell line ($4T1_P$), thereby facilitating a biologically relevant comparison between the two related clones (Figure 2A, see STAR Methods and Figure S1A). The exposure of tumor cells to carcinogen results in increased total tumor mutational burden (tTMB), as previously demonstrated,[11,12] mimicking at least one potential tumor-dependent aspect of immunotherapy response. Using this model, and in-conjunction with mass cytometry (CyTOF) and flow cytometry, we confirmed that mutagenesis resulted in tumors with a higher degree of immunogenicity, characterized by reduced numbers of immunosuppressive cells (e.g., G- and M-MDSCs and PDL1$^+$ cells), increased numbers of anti-tumor immune cells (e.g., activated B and T cells) and elevated Granzyme B levels (Figures 2B–2D and S1B–S1I). $4T1_M$ and $4T1_P$ therefore constitute suitable models to initially study immunotherapy response.

Preclinical and clinical studies, thus far, have focused on a variety of immune cells as potential biomarkers for immunotherapy—most notably and primarily T cells[6,13] but also MDSCs.[14] MDSCs, which include granulocytic and monocytic subtypes, constitute a widely variable, heterogeneous group of cells that are strongly linked to negative outcomes in patients

CellPress
OPEN ACCESS

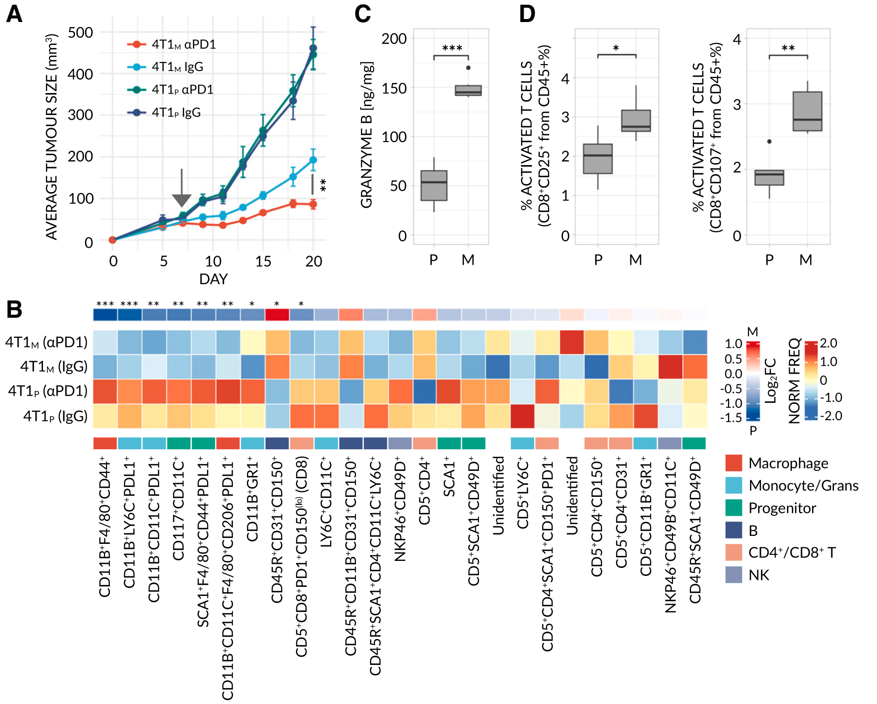

Figure 2. A mutagenized 4T1 breast cancer model displays an immunogenic phenotype

(A) Averaged tumor growth profile for BALB/c mice implanted with parental (non-responsive) or mutagenized (responsive) 4T1 breast cancer ($4T1_P$ - (P) and $4T1_M$ - (M), respectively), and treated with αPD1 or control IgG antibodies (n = 5 mice/group). Raw data are available in Figure S1A. Significance was assessed by means of two-sample KS-test (**, $p < 0.001$).

(B) $CD45^+$ cells from the tumor microenvironment (TME) of $4T1_P$ (205,678 cells) and $4T1_M$ (236,251 cells) tumors were segregated into 25 distinct, unsupervised clusters. A heatmap of normalized, scaled cluster frequencies per-sample is shown. Cluster genotypes and parental cell-types were annotated based on the expression of all markers, inspected in parallel (see Figure S1B). Generalized linear models (GLMs) were fit to detect differentially abundant ($4T1_P$ vs. $4T1_M$, combined treatments) clusters. Treatment was initiated at a tumor size of ~50 mm^3 (arrow). Significance was assessed by means of FDR-corrected, Bayesian-moderated t tests (*, FDR < 0.01; **, FDR < 0.001; ***, FDR < 0.0001).

(C) Granzyme B concentrations in untreated tumor lysates, as measured by ELISA (n = 6 mice/group).

(D) Frequency of activated ($CD25^+$ or $CD107^+$) cytotoxic T cells, as determined by flow cytometry, in 4T1 tumors (n = 6 mice/group). All CyTOF samples, tumors and lysates were taken at endpoint (tumor size of ~200–500 mm^3). In (C and D), significance was assessed by means of a one-way Mann-Whitney test (*, $p < 0.01$; **, $p < 0.001$; ***, $p < 0.0001$).

with cancer.[15] However, contrasting reports suggest different myeloid subsets are associated with anti-tumor activity.[16] Given the pleiotropic roles myeloid cells evidently play in tumor biology, and the prior efforts of the community to characterize T cell-based biomarkers, we focused our search on myeloid cells. To this end, we isolated $GR1^+$ cells, representing both monocytic and granulocytic immune cells in mice, from non-responsive and responsive 4T1 tumors and subsequently performed scRNA-seq to map $GR1^+$ subpopulations in detail. UMAP analysis of all $GR1^+$ cells isolated from these tumors revealed two major, coherent populations, representing monocytic and granulocytic phenotypes, as is congruent with previous literature[17] (Figure S2A). Within the monocytic compartment, we observed significant differences in macrophage subsets consistent with previous findings.[18] Namely, non-responsive mice were enriched for immunosuppressive, M2 macrophage-like cells while mice responsive to anti-PD1 were enriched for inflammatory, M1 macrophage-like cells (Figure S2B). No further differences were noted, prompting us to search for biomarkers within the granulocytic population. Counterintuitively and in contrast to the known immunosuppressive effects of myeloid cells, including granulocytic MDSCs,[19] we identified a subpopulation of neutrophil whose abundance significantly increases as a function of anti-PD1 response (Figures 3A and S2C). Moreover, this subpopulation exhibited up-regulated expression of 192 different genes (>1.5 log_2 fold-change), including 30 genes with a >2 log_2 fold-change—providing a large pool of candidate biomarkers, associated with this cellular subpopulation, for immunotherapy response within the phenotypically stable 4T1 model (Table S1A).

To narrow down the selection, and aid clinical translation, we reasoned that a successful biomarker must fit the following criteria: (1) the marker is mechanistically understood e.g., it is induced by a, or a series of, signaling pathway(s) identifiable in the data or active in the tumor microenvironment, (2) the marker is present on a host-cell (e.g., neutrophil), but is induced by tumor-intrinsic activity (see: introduction for rationale), (3) expression of the marker is predominately found in a metastable or highly differentiated cell state, as opposed to a transient state that may be difficult to consistently detect *in vivo* and finally, for practicality (4) the marker gene encodes a cell surface marker, permitting cost-effective analysis by simple flow cytometry.

Following this logic, we initially performed trajectory analysis for all neutrophilic cells and deduced the trajectory's directionality by calculating vectors of RNA velocity (Figures 3B and S2D) (see STAR Methods for details). Such an approach not only determines the endpoint(s) of cellular differentiation, but also provides mechanistic insight by revealing which biological processes and transcription factors are periodically activated as differentiation proceeds. Interestingly, we observed a branched, dual-lineage trajectory that diverges at an early stage (i.e., at the progenitor level) yet ultimately converges upon a single cell state (Figure 3B). Importantly, and consistent with the RNA-velocity-inferred direction, known progenitor genes are expressed at early pseudotime values (Figures S2E–S2I)[20]—suggesting this trajectory captures pathways of legitimate cellular development. We hypothesized that the convergence of two lineages toward the same cell state may reflect a common, underlying differentiation program. We therefore used a trajectory alignment algorithm[21] to match "homologous" segments of each lineage to one another and identified multiple, shared modules of genes—present in both branches—whose expression alters as a function of pseudotime (Figure 3C). By calculating the

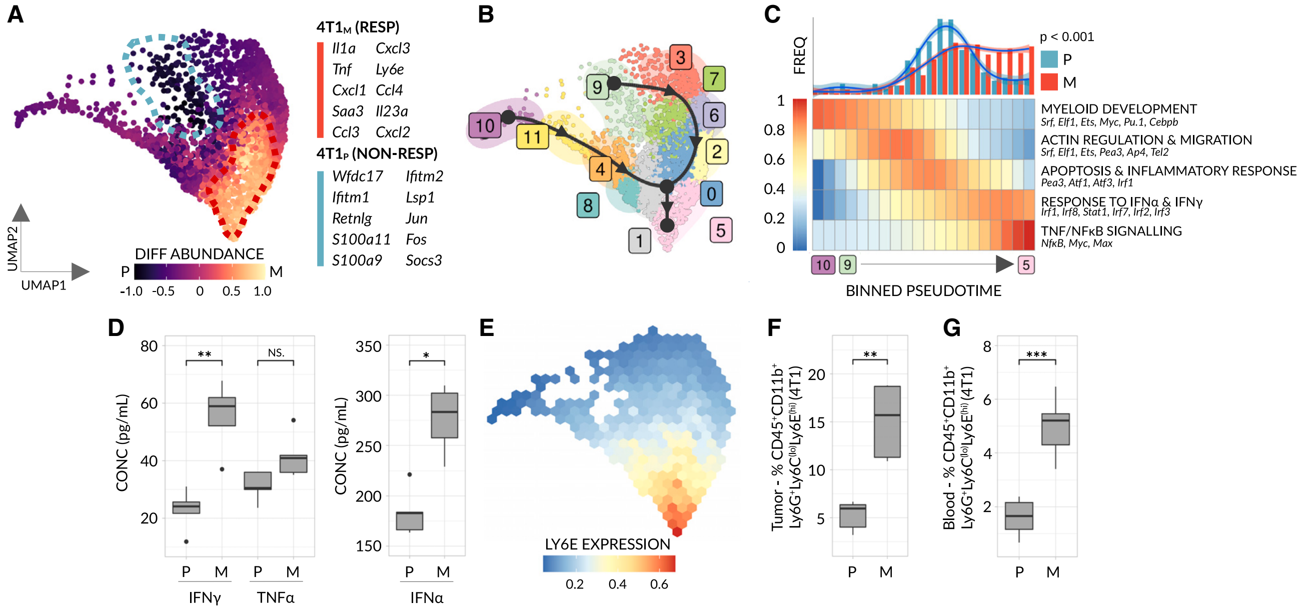

Figure 3. IFN-stimulated, Ly6E$^{(hi)}$ neutrophils mark response to αPD1 in 4T1 breast cancer

10X scRNA-seq was performed on GR1$^+$ cells obtained from parental (4T1$_P$) (non-responsive) and mutagenized (4T1$_M$) (responsive) 4T1 breast cancer tumors (n = 3 mice pooled/group). (A) UMAP plot of 2886 filtered, GR1$^+$ neutrophils (4T1$_P$ = 681 cells, 4T1$_M$ = 2185 cells), with cells colored based on differential abundance score. Two significantly enriched, cellular neighborhoods (dotted lines) are highlighted (see also Figure S2C). The top 10, most significant marker genes of each neighborhood are listed (FDR < 0.001, $\log_2$ fold-change > 1.5). Monocytic cells (not shown) were discarded from the analysis (see: Figure S2).

(B) Trajectory analysis for 12 distinct, GR1$^+$ granulocytic clusters. Solid black line = trajectory lineages, which form the basis of the pseudotemporal ordering as inferred by partition-graph based abstraction (PAGA). Black arrows = simplified RNA-velocity (for raw data, see Figure S2D).

(C) Top: A histogram of binned cell frequencies as a function of aligned pseudotime. Smoothed distributions, generated by loess regression, are overlaid. Significance was assessed by means of two-sample KS-test. Bottom: A heatmap of normalized, binned enrichment scores for all gene modules that display a significant association with pseudotime (FDR < 0.01). Only gene-modules common to both lineages are shown.

(D) Boxplots showing the concentration of IFNγ, TNFα and IFNα within untreated 4T1 tumor lysates (n = 4-5 mice/group).

(E) Binned, normalized expression of Ly6E. Data were imputed for visual clarity.

(F and G) Frequencies of Ly6E$^{(hi)}$ neutrophils, as determined by flow cytometry (n = 5-10 mice/group), in 4T1 tumors (F); and the blood of 4T1 bearing mice (G); For the gating strategy see Figure S3A. In (D, F, and G), significance was assessed by means of a one-way Mann-Whitney test (NS, p > 0.01; *, p < 0.01; **, p < 0.001, ***, p < 0.0001).

density of cells across the resulting aligned trajectory on a per-sample basis (Figure 3C - top) and characterizing each shared module (Figure 3C - bottom), we observed that neutrophils from non-responsive mice typically fail to progress beyond a progenitor-like, apoptotic state. In contrast, neutrophils from responsive mice differentiate further to a terminal state marked by response to interferon α/γ (IFNα/γ) and NFkB/TNFα signaling, suggesting exposure to IFN is a major driving force behind this differential progression. Consistent with this, previous studies have shown a link between IFNα/γ levels and response to immunotherapy.[22] When examining 4T1 tumors at the protein level, we observed a similar correlation between IFNα/IFNγ/TNFα levels and response, validating our scRNA-seq results (Figure 3D, see STAR Methods). Therefore, in order to select a candidate biomarker that fulfills our criteria (see previous text), we screened all 192 differentially expressed genes (Table S1A) for IFN-stimulated genes (ISGs) residing at the cell surface. Ly6E, a known ISG[23] and the only cell-surface marker to fulfill our criteria, was found to have a high expression-weighted pseudo-time value and constitute a prime biomarker candidate by which to assay this subtype of neutrophil (Figure 3E). Specifically, a high frequency of Ly6E^{hi} neutrophils in the tumor significantly correlates with immunotherapy response in the 4T1 model (Figure 3F and see S3A; gating strategy). Despite the discovery of Ly6E^{hi} neutrophils in tumor samples, we hypothesized that these cells may also additionally form in or cycle back into the blood. Indeed, Ly6E^{hi} neutrophils similarly mark response when assayed in the blood of mice bearing 4T1 tumors (Figure 3G), and importantly, the ability of Ly6E^{hi} neutrophils to distinguish responsive and non-responsive mice is established at early stages of tumor growth (~50 mm^3) —collectively suggesting Ly6E^{hi} neutrophils may serve as a predictive, blood-borne biomarker of anti-PD1 response in this model.

Ly6E^{hi} neutrophils overcome resistance to anti-PD1 therapy

Biomarkers can be surrogate—that is, passive bystanders generated as a byproduct of the main biological mechanism(s) underpinning immunotherapy response (e.g., the presence of IFNα/γ in the microenvironment of responding tumors) —or they may be functionally involved in response itself. We reasoned that functionally active biomarkers may possess a wider degree of applicability, beyond a single preclinical model or cancer-type. Thus, to distinguish between these two possibilities, we artificially generated Ly6E^{hi} neutrophils *in vitro* by exposing GR1$^+$ cells to a cocktail of IFNα/γ (Figure 4A), as

CellPress
OPEN ACCESS

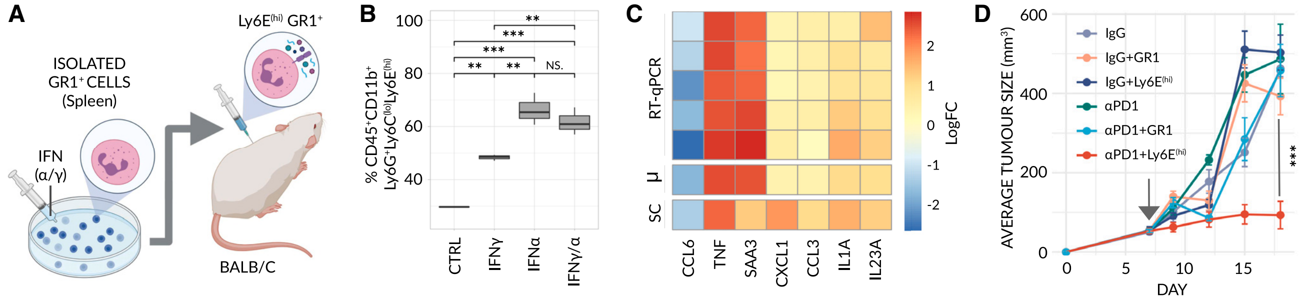

Figure 4. Ly6E$^{(hi)}$ neutrophils sensitize non-responding 4T1 tumors to αPD1 treatment

(A) Schematic of adoptive transfer. Isolated GR1^{+} cells are treated *in vitro* with IFNγ/α, inducing a Ly6E$^{(hi)}$-like state, characterized by secretion of effector molecules, and injected into BALB/c mice bearing parental, non-responsive 4T1 breast tumors.

(B) Frequency of Ly6E$^{(hi)}$ neutrophils following exposure of GR1^{+} cells to IFNγ, IFNα or both, as determined by flow cytometry (n = 3 mice/group). Significance was assessed by means of a one-way ANOVA and Tukey's post-hoc HSD test (NS, $p > 0.01$; **, $p < 0.001$; ***, $p < 0.0001$).

(C) A heatmap comparing normalized, $\log_2$-fold changes from RT-qPCR (treated [+IFNγ/α] vs. untreated control GR1^{+} cells) and scRNA-seq (Ly6E$^{(hi)}$ neutrophils vs. all remaining neutrophils) (n = 7 biological repeats/group). SC = scRNA-seq. μm = averaged RT-qPCR values.

(D) Averaged tumor growth profiles for mice bearing parental, non-responsive 4T1 breast tumors treated with either a monotherapy (control IgG or αPD1) or a combined therapy, with GR1^{+} or Ly6E$^{(hi)}$ neutrophils, as specified (n = 6 mice/group). A time-course of the adoptive transfer is depicted in (Figure S4A). Raw data are available in (Figure S4B). Treatment was initiated at a tumor size of ∼50 mm^3 (arrow). Significance was assessed by means of two-sample KS-test (***, $p < 0.0001$).

informed by scRNA-seq analysis (Figures 3C and 3D). To ensure that the resulting cells resemble the Ly6E^{hi} phenotype observed in our scRNA-seq data, we analyzed the induction of Ly6E at the protein level and the mRNA expression levels of selected differentially expressed, secreted factors by RT-qPCR, based on our scRNA-seq data. Firstly, we observed a strong induction of Ly6E on the surface of neutrophils following IFN treatment (Figure 4B). Secondly, we observed a striking correlation between the $\log_2$ fold-changes of the RT-qPCR (treated vs. untreated) and the scRNA-seq (response vs. non-response) (Figure 4C), collectively suggesting these cells are analogous.

Subsequently, we sought to test, *in vivo*, the effect of these generated cells on tumors resistant to anti-PD1. We, therefore, administered Ly6E^{hi} neutrophils, by adoptive transfer (see Figure S4A, for treatment protocol), to mice bearing non-responsive 4T1 tumors, and observed a significant reduction in tumor growth following anti-PD1 therapy but no efficacy of these cells as a monotherapy (Figures 4D and S4B). Consistent with these results, we examined the levels of various immune cells in a separate experiment and found that the frequency of blood-borne and tumor-infiltrating activated cytotoxic CD8^{+} T cells was significantly higher in mice treated with both anti-PD1 and Ly6E^{hi} neutrophils relative to those treated with either monotherapy alone (Figures S4D–S4F). This trend was further recaptured when measuring intra-tumoral granzyme B levels (Figure S4G). Of note, we identified fluorescently labeled Ly6E^{hi} neutrophils in treated tumors (Figure S4H), further indicating that Ly6E^{hi} neutrophils successfully infiltrate and play a role in the responding tumor microenvironment.

Given the IFN-stimulated phenotype of Ly6E^{hi} neutrophils, we next evaluated whether IFN-γ and IFN-α (IFNγ/α) alone can sensitize resistant tumors to the same extent. While mice treated with a combination of IFNγ/α and anti-PD1 display a marginal reduction in tumor growth, it is not significant (Figures S4C and S4I) and no change in the levels of activated CD8^{+} T cells was observed in either the blood or the tumor (Figures S4J and S4K). Nevertheless, despite the lack of response, the levels of Ly6E^{hi} neutrophils in the tumor were significantly elevated by IFNγ/α treatment, reinforcing the fact that IFN induces Ly6E^{hi} neutrophils both *in vivo* and *in vitro* (Figures S4L and S4M and 4B, respectively). This apparent paradox suggests IFN-γ and/or IFN-α, when given systemically, mediate additional pleiotropic effects beyond the generation of Ly6E^{hi} neutrophils—effects which inhibit immunotherapy response and overwrite the ability of Ly6E^{hi} neutrophils to overcome non-responsiveness. Furthermore, our results suggest that Ly6E^{hi} neutrophils themselves represent an isolated, distinctly anti-tumorigenic effect of IFN. Such findings are consistent with but potentially build upon the apparent ineffectiveness of systemic IFN-treatment in augmenting ICI therapy in humans.[24]

The STING signaling pathway accounts for IFN-induced Ly6E^{hi} neutrophils which in-turn directly support anti-tumor immunity

Cytosolic double-stranded DNA (dsDNA), generated under conditions of cellular stress, hypoxia or chromosomal instability, is known to induce tumor-intrinsic STING pathway activity and the subsequent secretion of IFNs (e.g., IFNα) from cancer cells.[25,26] Given the IFN-stimulated phenotype of Ly6E^{hi} neutrophils, the use of a model with high mutational burden, and our desire to identify a biomarker patterned by tumor-intrinsic properties, we asked whether STING signaling is responsible for the generation of these cells in the tumor microenvironment. To this end, we quantified the levels of STING-pathway associated factors in non-responsive $4T1_P$ and responsive $4T1_M$ clones. We observed significantly higher levels of cytosolic dsDNA and a significant up-regulation of STING and its downstream signaling components (IRF3, NF-κB, and native ISG15 [15 kDa]) in $4T1_M$ relative to $4T1_P$ (Figures 5A, 5B, and S5A). Consistent with this, $4T1_M$ cells secrete higher levels of IL-6, up-regulate cell-surface MHCI, and down-regulate PDL1—all known readouts of STING activity (Figures S5B–S5D).[27,28] Importantly, these trends are reversed with use of the STING inhibitor, H151 (Figures S5B–S5D). Interestingly, $4T1_M$ tumors show a reduced level of

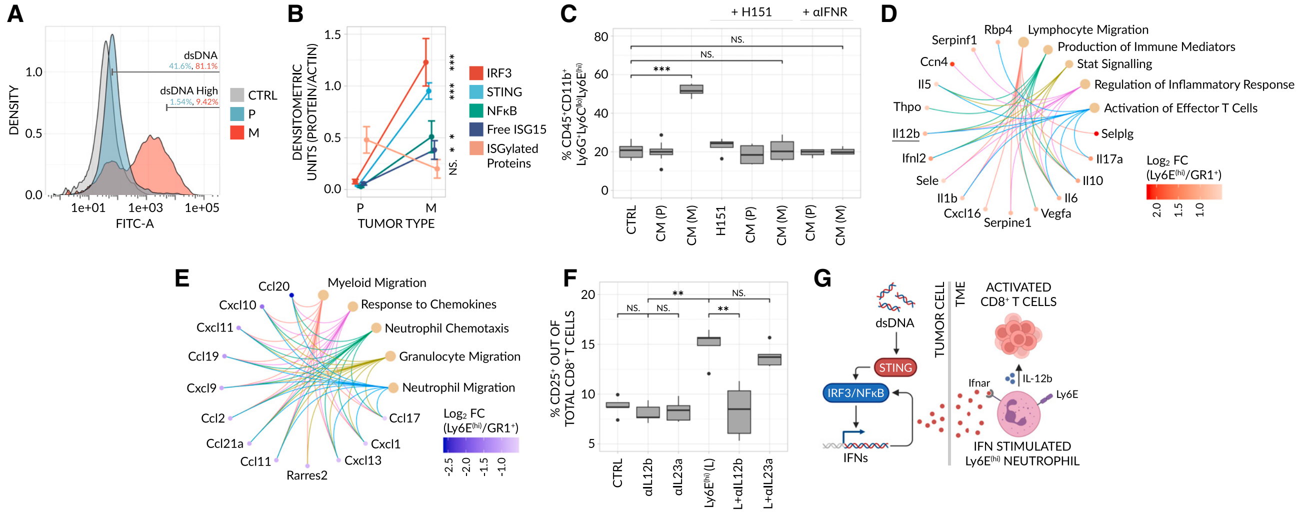

Figure 5. Tumor-intrinsic STING activity induces the Ly6E$^{(hi)}$ phenotype and in-turn supports activation of effector T cells

(A) Density plots of dsDNA levels in cultured $4T1_P$ and $4T1_M$ cell-lines, as determined by α-dsDNA staining and flow cytometry. dsDNA levels were quantified relative to an unstained, IgG2a isotype control (CTRL) (n = 5 biological repeats/group).

(B) Densitometry quantification of western blots (see Figure S5A) for STING-pathway related proteins in $4T1_P$ and $4T1_M$ tumor lysates (n = 3–4 biological repeats/group). Each protein was normalized relative to an actin control.

(C) Isolated GR1$^+$ cells were cultured *in vitro* with conditioned media generated from $4T1_P$ (P) or $4T1_M$ (M) tumors in the presence or absence of the STING-inhibitor H151 or αIFNR-α/γ, and the frequencies of Ly6E$^{(hi)}$ neutrophils were determined by flow cytometry (n = 6 biological repeats/group). CTRL = GR1$^+$ cells only.

(D and E) Conditioned media was generated from GR1$^+$ cells or IFNαγ-induced Ly6E$^{(hi)}$ neutrophils, and subsequently assayed on a cytokine array (n= 3 mice pooled/group). Hyper-geometric, over-representation tests and the Gene Ontology (GO) database were used to determine enriched pathways for Ly6E$^{(hi)}$ neutrophils (D); and GR1$^+$ cells (E). Only differentially expressed proteins with a $\log_2 FC > 0.35$ were included and only significant pathways (FDR < 0.01) are shown.

(F) Isolated CD8$^+$ T cells were cultured *in vitro* with α-IL-12b or α-IL23a neutralizing antibodies, with or without conditioned media from IFNα/γ-induced Ly6E$^{(hi)}$ neutrophils (L), and the levels of activated CD25$^+$CD8$^+$ T cells were determined by flow cytometry (n = 5 mice/group). CTRL = CD8$^+$ T cells only. In (B, C, and F), significance was assessed by means of a one-way ANOVA and Tukey's post-hoc HSD test (NS, $p > 0.01$; *, $p < 0.01$; **, $p < 0.001$; ***, $p < 0.0001$).

(G) Schematic of the proposed mechanism. Tumor-intrinsic STING activity, as induced by cytosolic dsDNA as a result of hypoxia, genomic instability and/or cell stress, transcriptionally activates an IFN response. Tumor-secreted IFNα, for example, subsequently binds to Ifnar-expressing Neutrophils in the TME, inducing the Ly6E$^{(hi)}$ phenotype and in-turn activation and proliferation of CD8$^+$ T cells through IL-12b. Collectively, this supports immunotherapy response and anti-tumor activity. It is important to note that this mechanism is STING-specific, but that Type II IFNs (e.g., IFNγ)—derived from other sources or mechanisms—are also able to elicit equivalent effects, as shown in our work.

ISGylated proteins (Figure 5B), suggesting ISGylation machinery is suppressed or abnormal in these tumors despite robust STING and ISG15 induction. Nevertheless, and critically, conditioned media of $4T1_M$ cells strongly induces the Ly6E^{hi} neutrophil phenotype *in vitro*, in a STING-dependent manner, and this induction is reversed when blocking IFN receptors (IFNRα/γ) (Figure 5C). In contrast, no such dynamics are seen with media derived from $4T1_P$. Consistent with these results, IFNRα/γ are expressed at a high level on GR1$^+$ cells (Figure S6A) - confirming their ability to respond to IFN. Interestingly, receptor expression is maintained on Ly6E^{hi} cells (Figure S6A) and we further show that the higher levels of IFNα/γ previously observed within $4T1_M$ tumors (see: Figure 3D) are entirely STING-dependent (Figures S6B and S6C). In order to expand upon these observations, we assessed the effects of IFNR-α/γ inhibition *in vivo*. Mice bearing responsive $4T1_M$ tumors—treated with αIFNR-α/γ—were no longer able to mount an effective response to anti-PD1 (Figures S6D and S6E) and this lack of response was marked by lower levels of Ly6E^{hi} neutrophils (Figure S6F). Importantly, adoptive transfer of Ly6E^{hi} neutrophils was able to rescue immunotherapy response despite IFNR-α/γ blockade (Figures S6D–S6F). Taken together, our results strongly suggest that STING activation—intrinsic to $4T1_M$ responsive cancer cells—accounts for the induction of Ly6E^{hi} neutrophils, as mediated by IFN, and in-turn the ability of these cells to predict but also induce immunotherapy response.

Given the ability of Ly6E^{hi} neutrophils to mediate immunotherapy response in 4T1-bearing mice (see Figures 4D, S4, and S6D–S6F), we sought to uncover the Ly6E^{hi}-dependent molecular mechanisms responsible for this. Since adoptive transfer of Ly6E^{hi} neutrophils into $4T1_P$-bearing mice induced cytotoxic CD8$^+$ T cell activity (see Figures S4E and S4F), we explored whether Ly6E^{hi} neutrophils directly mediate this activation and whether this activity is dependent on Ly6E itself, or through secreted factors induced post-IFN-stimulation. To address these two questions, we first co-cultured Ly6E^{hi} neutrophils or unstimulated GR1$^+$ cells with CD8$^+$ T cells. While Ly6E^{hi} neutrophils promote the proliferation and activation of cytotoxic CD8$^+$ T cells, GR1$^+$ cells substantially inhibit such activities (Figures S7A–S7D). Consistent with this, Ly6E^{hi} neutrophils significantly promote T cell mediated tumor cell killing *in vitro*, relative to control cultures (Figure S7E). We subsequently

knocked Ly6E down in the bone-marrow of mice (Figure S8A, see STAR Methods), and repeated these experiments. Importantly, no change in response to anti-PD1 was observed *in vivo* (Figure S8B) and all positive effects of IFN-induced Ly6E^{hi} neutrophils on T cells and T cell-mediated tumor killing were retained regardless of Ly6E status (Figures S8C–S8G)—suggesting that Ly6E has no functional role in the mechanism of response, but rather serves solely, in our study, as means to assay this subpopulation of neutrophil.

Therefore, we next compared the secretome of Ly6E^{hi} neutrophils, relative to all other GR1^{+} cells, in order to determine the potential mechanism(s) underlying the induction of T cell activation. Based on pathway analysis of differentially expressed proteins, Ly6E^{hi} neutrophils support the activation and positive regulation of CD8^{+} T cells—through cytokines such as IL-12b, IL-1β, IL-6, and IL-10—while unstimulated GR1^{+} cells support an immunosuppressive tumor microenvironment through the recruitment of additional immunosuppressive myeloid cells (Figures 5D and 5E). Consistent with this and relative to all other GR1^{+} neutrophil subsets, Ly6E^{hi} neutrophils are significantly down-regulated at the mRNA level for secreted, immunosuppressive factors such as S100A8, S100A9, and CCL6,[29–31] while up-regulated for pro-inflammatory factors such as TNF-α, IL23a, IL-12b, and IL-1α (Table S1B). To validate this further, we co-cultured Ly6E^{hi} neutrophils with CD8^{+} T cells in the presence or absence of neutralizing antibodies targeting IL-12b and IL23a, both of which were up-regulated in Ly6E^{hi} neutrophils compared to GR1^{+} cells. Interestingly, and in line with a recent publication,[32] we found that IL-12b but not IL23a induced the activity of CD8^{+} T cells (Figure 5F). These results therefore suggest that Ly6E^{hi} neutrophils may augment cytotoxic CD8^{+} T cell activity, through secretion of IL-12b.

To establish a clear order of events, we further tested if the levels of Ly6E^{hi} neutrophils are reciprocally dependent upon T cell activity by utilizing SCID mice lacking an adaptive immune system and found this not to be the case. Instead, we observed that the ability of blood-borne Ly6E^{hi} neutrophils to distinguish responding and non-responding 4T1 tumors in immunocompetent mice remains intact within SCID mice (Figures S7F–S7G). Collectively, our results suggest that Ly6E^{hi} neutrophils not only serve as a predictive biomarker for immunotherapy response in mice bearing 4T1 tumors but also: (1) are functionally involved in the mechanism of response; (2) operate upstream of T cells; (3) can be induced by an entity other than the adaptive immune system or host (e.g., tumor-intrinsic STING signaling, via IFNα or via IFNγ through yet-to-be characterized mechanisms); and (4) contribute to anti-tumor immunity by directly activating cytotoxic CD8^{+} T cells via IL-12b (Figure 5G).

Cross-validation of Ly6E^{hi} neutrophils as a biomarker for response in various preclinical tumor models

The majority of translational studies, to their detriment, continue to employ simplistic approaches involving only single mouse strains or cancer types. Therefore, the identification of Ly6E^{hi} neutrophils in one preclinical model prompted us to validate them as blood borne biomarkers in a diverse array of additional models capturing both tumor- and host-dependent variation, as both aspects play a key role in drug efficacy.[33] We therefore employed tumor models based on cell lines, encompassing: (1) clones with or without mutagenesis in two strains of mice (RENCA renal cell carcinoma, and Lewis Lung carcinoma (LLC)), as in our previous 4T1 approach; (2) cell lines that spontaneously respond to immunotherapy (EMT6 breast cancer); and (3) mixed background mice, containing variable baseline immune states, implanted with LLC tumors (Figure S9). In all cases, we observed that the frequency of Ly6E^{hi} neutrophils predicts response to anti-PD1 prior to treatment—to a significant degree and in a model agnostic manner (Figures S9A–S9D). Collectively, our data suggest that IFN-stimulated, Ly6E^{hi} neutrophils are a potential "pan-mechanistic" marker for therapy outcome in mouse, whether the response is driven by tumor-, host-dependency or strain-specific differences and that IFN-secretion into the tumor microenvironment may therefore be a common step in the mechanism of response.

Ly6E^{hi} neutrophils predict immunotherapy response in human

Species-specific differences typically hinder the ability to translate findings, such as a biomarker, from mouse to human.[34] To help overcome this, we employed a set of pre-clinical models (see Figure 1) to identify Ly6E^{hi} neutrophils as a potential "pan-mechanistic" biomarker in mouse with a greater degree of confidence that the marker may be conserved in humans. Nevertheless, it remained unclear whether Ly6E would be a marker of the same, IFN-stimulated cell state in human. To address this limitation and further bridge the cross-species gap, we first built a functional signature based upon the biological processes that mark response in mouse (see Figure 3C), namely IFNα/γ response and NF-κB/TNFα signaling. Subsequently, we analyzed public, scRNA-seq data from the blood of 8 patients with non-small cell lung cancer (NSCLC) obtained prior to treatment and applied the mouse-derived signature to all 6607 identifiable human neutrophils[35] (Figure 6A). We observed a cluster of cells highly enriched for our signature, marked by genes induced by IFN (Figure 6B). Notably, this cluster displayed a high level of Ly6E expression, suggesting Ly6E is an appropriate marker by which to assay these cells in human (Figure 6C). Subsequently, to test whether Ly6E^{hi} neutrophils predict response to immunotherapy in humans, we obtained pre-treatment peripheral blood mononuclear cells (PBMCs) from a limited, independent mixed cohort of patients with advanced metastatic NSCLC ($n = 50$) and malignant melanoma ($n = 59$) predominately treated with ICI-based therapy and quantified the levels of Ly6E^{hi} neutrophils. For the sake of clarity, it is important to note that low-density neutrophils found in chronic disease states are present in PBMC fractions.[36] As in mouse, high levels of Ly6E^{hi} neutrophils were strongly correlated with response and positive, clinical outcome (Figures 6D, 6E, and S3B for gating strategy). Remarkably, Ly6E^{hi} neutrophils stratify between non-responder and responder groups (AUC $\approx$ 0.9) in both cancer types, whereas pre-existing biomarkers, namely, PDL1 IHC and total neutrophil count measured in the same group of patients with NSCLC, underperformed (AUC $\approx$ 0.6 and 0.75, respectively) (Figure 6F). To further strengthen and broaden these findings, we used cell-specific deconvolution and expression imputation methods to estimate the levels of Ly6E^{hi} neutrophils in 1,237 publicly available, bulk RNA-seq samples taken from six different cancer types prior to immunotherapy treatment.[37–40] We

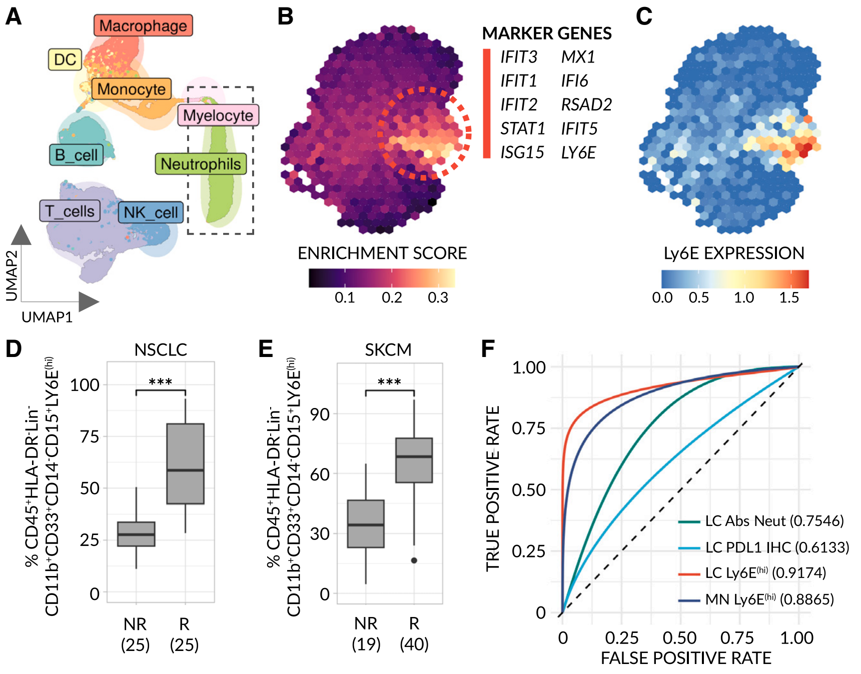

Figure 6. Ly6E$^{(hi)}$ neutrophils serve as a predictive biomarker for immunotherapy response in humans

(A) UMAP plot of 11702 filtered, CD45$^+$ cells taken from publicly available non-small cell lung cancer (NSCLC) scRNA-seq data (patient blood samples at baseline, n = 8)[35], with cells colored by cell type.

(B) Binned UMAP plot of isolated neutrophils (dotted box in (A)), with cells colored by the extent of their enrichment for a Ly6E$^{(hi)}$ functional signature. The top 10, most significant marker genes of the enriched cluster (dotted lines) are listed (FDR < 0.001, $\log_2$ fold-change > 1.5).

(C) Binned, normalized expression of Ly6E. Data were imputed for visual clarity.

(D and E) Frequency of Ly6E$^{(hi)}$ neutrophils in the blood of an independent cohort of patients with NSCLC (n = 50) (D) and skin cutaneous melanoma (SKCM) (n = 59) (E), as determined by flow cytometry. For the gating strategy see Figure S3B. Data are stratified by RECIST categories at 3 and/or 6 months (NR = progressive disease (PD) and R = stable disease (SD), partial or complete response (P/CR)). Sample sizes are denoted for each individual group. Significance was assessed by means of a one-way Mann-Whitney test (***, p < 0.0001). (F) Smoothed area under the curve (AUC)-receiver operating characteristics (ROC) plots for Ly6E$^{(hi)}$ neutrophils (95% CIs: 0.855–0.9705 (NSCLC - LC), 0.7913–0.9606 (Melanoma - MN)), absolute neutrophil count (Abs Neut) (95% CIs: 0.534–0.9328 (in NSCLC)) and tumor PDL1 IHC (95% CIs: 0.3554–0.9338 (in NSCLC)) in our cohort of patients (NR vs. R). Confidence intervals were determined using 1,000 stratified, bootstrap replicates.

observed that, in all but one dataset, neutrophils in responders relative to non-responders are highly enriched for a Ly6E^{hi}-neutrophil derived, IFN-stimulated signature (Neut$_{IFN}$-15, genes: IFIT1, MX1, HERC5, IFI6, ISG15, IFIT3, RSAD2, GBP1, IFIT2, XAF1, PARP9, UBE2L6, IRF7, PARP14, and APOL6)—including in urothelial bladder carcinoma, glioblastoma, NSCLC, renal cell carcinoma, melanoma, and stomach adenocarcinoma datasets, at the pre-treatment stage (Figure 7A, top). Conversely, the previously published IFN-γ 6 signature,[41] which has no overlap in genes with Neut$_{IFN}$-15, underperforms on these datasets (average AUC 0.62 vs. AUC 0.88, respectively) (Figure 7A, bottom and S10 for raw data). Moreover, in one dataset where pre-existing biomarkers (PDL-1 IHC, tTMB, and STK11/KEAP1 status) were measured, Neut$_{IFN}$-15 predicted outcome with significantly higher accuracy (Figure 7B). Of note, in 203 samples taken post ICI therapy, the ability of Ly6E^{hi} neutrophils to stratify between responders and non-responders is weakened (Figure 7A). These results, taken together, suggest that the levels of Ly6E^{hi} neutrophils—whether measured in the blood or the tumor—serve as a predictive biomarker for immunotherapy response in both mice and humans across a multitude of different tumor types.

DISCUSSION

The efficacy of immunotherapy is governed by complex mechanisms dependent upon the interactions between host (e.g., the immune system) and malignant cells. By narrowing our search to host cell biomarkers not only predictive of response, but for which we also have a tumor-dependent mechanism, we discovered interferon-stimulated, Ly6E^{hi} neutrophils as a blood borne, predictive biomarker with potentially high predictive power in both mice and humans (AUC ≈ 0.9 in humans). Importantly, Ly6E^{hi} neutrophils appear to remain predictive in a diverse array of cancer types. Our approach may therefore have revealed a "pan cancer" biomarker that can be assayed in a cost-effective manner by liquid biopsy, however further clinical validations are required.

Neutrophilic GR1$^+$ cells or MDSCs are ordinarily and strongly pro-tumorigenic, acting to suppress anti-tumor immunity.[19] Yet, Ly6E^{hi} neutrophils exhibit anti-tumorigenic properties, induce immunotherapy response in mice and enhance immunity against tumors, further highlighting the plasticity and importance of myeloid cell state in the tumor microenvironment.[42] In highly mutated, murine 4T1 tumors, induction of tumor-intrinsic STING activity is responsible for IFN-secretion and the generation of Ly6E^{hi} neutrophils in the tumor microenvironment, mediated specifically by IFNα/γ. Due to technical limitations, it remains unclear if STING activity is the driving force behind the Ly6E^{hi} phenotype in all cases and cancers. Nevertheless, given the broad predictive power of our biomarker, and the fact that the Ly6E^{hi} phenotype is induced by IFN, localized IFN activity in the tumor microenvironment may prove to be a crucial and common step in the mechanism of immunotherapy response regardless of the exact source of IFN or the exact IFN involved in a given case (IFNα or IFNγ).[43] Consistent with this, studies demonstrate that IFNγ or its related pathways serve as predictors of immunotherapy response[41,44,45]—albeit with a lower predictive power than Ly6E^{hi} neutrophils. Moreover, up- and down-regulation of MHCI and PDL1 respectively, due in part to IFN stimulation, can also stratify between responsive and non-responsive tumors[46–48] and IFNs are currently under clinical

CellPress
OPEN ACCESS

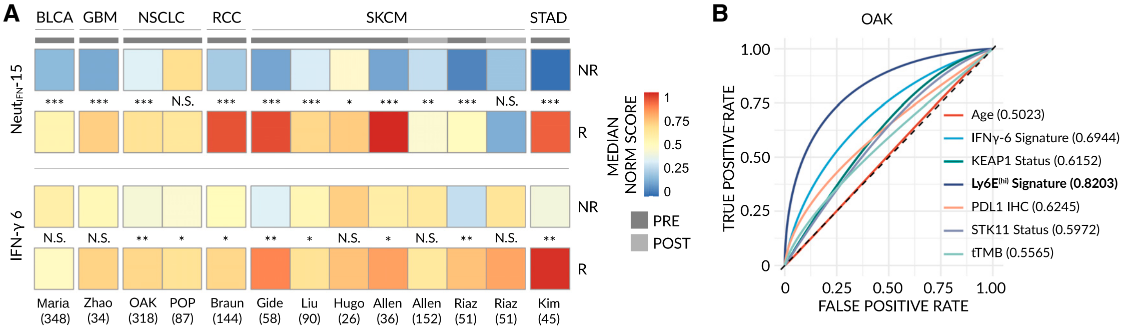

Figure 7. A Ly6E$^{(hi)}$ neutrophil-derived gene signature outperforms pre-existing biomarkers in the prediction of immunotherapy response

(A) Bulk RNA-seq expression profiles were obtained from 1,440 publicly available samples from 11 datasets across 6 cancer types[37–40] (see STAR Methods) and scored for a 15-gene Ly6E$^{(hi)}$-signature (Neut$_{IFN}$-15) (top) or a previously published 6-gene IFNγ-signature[41] (bottom). A heatmap of median, normalized enrichment scores for each dataset is shown and significant differences between groups were tested (NR vs. R). Samples were taken either pre-treatment (PRE) or post-treatment (POST). Raw data are available in Figure S10. BLCA = urothelial bladder cancer; GBM = glioblastoma multiforme; NSCLC = non-small cell lung cancer; RCC = renal cell carcinoma; SKCM = skin cutaneous melanoma; STAD = stomach adenocarcinoma. Significance was assessed by means of a one-way Mann-Whitney test (NS, $p > 0.01$; *, $p < 0.01$; **, $p < 0.001$, ***, $p < 0.0001$).

(B) Smoothed area under the curve (AUC)-receiver operating characteristics (ROC) plots for total tumor mutation burden (tTMB) (95% CIs: 0.4865-0.6722), Age (95% CIs: 0.4374-0.5766), PDL1 immunohistochemistry (IHC) (95% CIs: 0.5534-0.7172), STK11 mutational status (95% CIs: 0.5246-0.6874), KEAP1 mutational status (95% CIs: 0.5334-0.7085), IFNγ-6 signature scores (95% CIs: 0.6253-0.7561) and Ly6E$^{(hi)}$ Neut$_{IFN}$-15 signature scores (95% CIs: 0.7714-0.9105) in data from the OAK NSCLC study[39] (NR vs. R). Confidence intervals were determined using 1,000 stratified, bootstrap replicates.

evaluation as a combinatorial therapy with ICIs.[49] However, IFN has also been shown to counter-intuitively exhibit pro-tumorigenic effects and promote resistance to anti-PD1 therapy.[50] Indeed, our study demonstrates that systemic IFNγ/α treatment in combination with anti-PD1 resulted in a non-significant reduction in tumor growth and no change in cytotoxic CD8$^+$ T cell activation. It is plausible that IFN acts, in part, via Ly6E^{hi} neutrophils to augment immunotherapy outcome but that additional, negative effects of IFN—or chronic, systemic IFN treatment[51]—"tip the scales" and counterbalance this. Regardless, our study further provides mechanistic insights into the complex role IFNs play in cancer biology and by "zooming in" and identifying a specific anti-tumorigenic effect of IFN, i.e., generation of Ly6E^{hi} neutrophils, it may be possible to develop therapeutic approaches that lack the negative aspects of IFN—as our adoptive transfer results suggest.

We show that Ly6E^{hi} neutrophils not only act as a biomarker but also function as an immunomodulator—sensitizing otherwise resistant tumors to anti-PD1 therapy, in part, by creating an environment permissive to CD8$^+$ T cell activation through secretion of known activating factors such as IL-12b.[52] Critically, Ly6E^{hi} neutrophils appear to act upstream of the central anti-tumor T cell response, potentially "priming" tumors to respond. Consistent with this, treatment-elicited neutrophils acquire an IFN-gene signature following treatment with anti-PD1, and are essential to the response process in humans.[32] Our work thus expands upon this study, by demonstrating the presence of predictive, IFN-stimulated neutrophils *prior* to treatment.

It is important to note that, while our preclinical work focused entirely on anti-PD1, our clinical cohort is mixed—comprising patients with metastatic NSCLC and melanoma (n = 109) treated with either ICI monotherapy (anti-PD1, anti-CLTA4, or anti-PDL1) or ICIs in combination with other treatment modalities (e.g., chemotherapy). Therefore, while Ly6E^{hi} neutrophils remained highly predictive in all cases, further prospective clinical studies, including those related to neoadjuvant settings,[53] should be designed to validate the robustness of these results within each treatment arm and the ability of Ly6E^{hi} neutrophils to differentiate between non-responders and responders in a variety of treatment scenarios and tumor backgrounds e.g., specific mutations. Nevertheless, our main conclusions were further supported by the analysis of publicly available bulk RNA-seq datasets taken pre-treatment from 1,237 patients with cancer who underwent ICI therapy. In all samples analyzed, except one, the enrichment of a Ly6E^{hi} neutrophil-derived gene signature (Neut$_{IFN}$-15) correlated strongly (average AUC > 0.9) with patients who responded to immunotherapy—suggesting Ly6E^{hi} neutrophils are widely applicable as a biomarker. Furthermore, additional limitations exist in this study. *First*, our preclinical models were based on cancer cell lines and did not include genetically engineered mouse models or patient-derived xenografts. While this is a preclinical limitation, clinically, we demonstrate the validity of Ly6E^{hi} neutrophils as a potential biomarker regardless of these limitations. Moreover, and consistently with previous publications,[11] we demonstrate that high mutational burden contributes to ICI-responsive tumors, as mutagenesis induces a high degree of immunogenicity. It is worth mentioning that high mutational burden does not necessarily correlate with ICI outcome[54]; however, for our preclinical approach, the use of this artificial model aided prospective prediction of ICI therapy outcome. *Second*, the detection of Ly6E^{hi} neutrophils in clinical samples lacks a clear, demarcated population of cells when analyzed by flow cytometry. Rather, the Ly6E^{hi} phenotype is defined in relative terms compared to other samples and the expression of Ly6E itself occupies a continuum as opposed to discrete positive or negative states. Thus, future clinical studies should focus on refining Ly6E^{hi} neutrophil identification by, for example, utilizing internal markers expressed by these cells, CITE-seq or cell surface marker screening in order to adequately stratify between responders and non-responders using predefined, absolute

thresholds. Alternatively, machine-learning classifiers may be able to determine an appropriate flow-based threshold—given a sufficiently large test cohort. *Third*, owing to their short half-life and fragility in peripheral blood,[55] neutrophils are typically overlooked or discarded as a source of potential biomarkers or biology, and the methodologies used to collect PBMCs in human often exclude neutrophils due to their high density. However, in diseased states such as cancer, a subset of neutrophils adopts a low-density state,[36] making them clinically accessible and warranting further studies. Our study, itself, further demonstrates that neutrophils can be reliably detected in frozen PBMCs obtained from human samples.

Overall, while there are a number of limitations to our study which deserve further exploration and clinical validation, we nevertheless provide strong evidence that IFN-stimulated, Ly6E^{hi} neutrophils predict ICI outcome and are functionally involved in the generation of response.

STAR★METHODS

Detailed methods are provided in the online version of this paper and include the following:

- KEY RESOURCES TABLE
- RESOURCE AVAILABILITY
  - Lead contact
  - Materials availability
  - Data and code availability
- EXPERIMENTAL MODEL AND SUBJECT DETAILS
  - The establishment of diverse models to study predictive biomarkers for immunotherapy
  - Cell lines and culture
  - Mouse tumor models
  - Blood collection from patients with cancer
- METHODS DETAILS
  - Cell line mutagenesis
  - Tumor lysate preparation and protein measurement
  - Cytokine array and biological pathway enrichment
  - Flow cytometry acquisition and analysis
  - Time of flight mass cytometry (CyTOF)
  - Adoptive transfer of Ly6E^{hi} neutrophils experiments
  - Real-Time quantitative PCR (RT-qPCR)
  - CD8^{+} T cell assay
  - Tumor cell killing assay
  - dsDNA acquisition
  - STING signaling pathway analysis
  - Ly6E knock-down in bone marrow cells
  - Single cell RNA sequencing on GR1^{+} cells
- QUANTIFICATION AND STATISTICAL ANALYSIS
  - CyTOF pre-processing and analysis
  - Single cell RNA-seq alignment and pre-processing
  - Classification of cell types
  - Dimensionality reduction, unsupervised clustering and differential abundance analysis
  - Data visualization
  - Differential gene expression analysis
  - RNA-velocity and trajectory inference
  - Gene modules and pathway analysis
  - Trajectory alignment
  - Human analysis of blood scRNA-seq
  - Cell-specific deconvolution
  - Statistical analysis

SUPPLEMENTAL INFORMATION

Supplemental information can be found online at https://doi.org/10.1016/j.ccell.2023.12.005.

ACKNOWLEDGMENTS

This work is supported primarily by an H2020 European Research Council Grant (771112) and the Israel Science Foundation (194/18) as awarded to Y.S., and NIH CA231325 awarded to S.S.O. M.B. is supported by Ariane de Rothschild Women Doctoral Program. T.J.C. is supported by RTICC-Rubinstein fellowship. A select number of illustrations were drawn using BioRender.

AUTHOR CONTRIBUTIONS

Conception and design: T.J.C. and Y.S. Acquisition of data: M.B., P.K., S.S.Z., A.B., R.H., I.K., A.D., B.M., R.M., J.H.S., A.V., Z.R., C.M., P.C., J.B., M.S., and M.L. Analysis and interpretation of data: T.J.C., M.B., M.L., M.S., J.B., P.C., A.A., S.S.O., and Y.S. Writing, review, and/or revision of the manuscript: T.J.C., A.A., S.S.O., and Y.S. Study supervision: T.J.C. and Y.S.

DECLARATION OF INTERESTS

M.B., T.J.C., and Y.S. declare that they hold a pending patent on the use of Ly6E^{hi} neutrophils as a predictive biomarker for immunotherapy. P.C. serves on the advisory board to AstraZeneca, Boehringer Ingelheim, Chugai, Pfizer, Novartis, MSD, Takeda and Roche; receives research funds from AstraZeneca, Amgen, Boehringer Ingelheim, Novartis, Roche, and Takeda, receives speaker honoraria from AstraZeneca, Janssen, Novartis, Roche, Pfizer, Thermo Fisher, Takeda. J.B. serves as a consultant to AbbVie, Amgen, AstraZeneca, Bayer, MSD, Merck-Serono, Roche, Takeda, BMS, Medison, Pfizer, and received research funds from Immunai, OncoHost. M.S. holds equity in Actym, Adaptive Biotechnologies, Amphivena, Asher, Evolveimmune, Intensity, Nextcure, Normunity, Oncohost, Johnson and Johnson, GlaxoSmith Kline; serves as a consultant to Adagene, Adaptimmune, Agenus, Alkermes, Alligator, Anaptys, Asher, Astra Zeneca, Biond, Biontech, Boston Pharmaceuticals, Bristol-Myers, Dragonfly, Evaxion, Evolveimmune, Genentech-Roche, Gilead, Glaxo Smith Kline, Ichnos, Idera, Immunocore, Incyte, Innate pharma, Iovance, iTEOS, Jazz Pharmaceuticals, Kadmon-Sanofi, Kanaph, Merck, Molecular Partners, Nextcure, Nimbus, Normunity, Numab, Ocellaris-Lilly, Oncohost, Ontario Institute for Cancer Research, Partner Therapeutics, Pfizer, Pierre-Fabre, PIO Therapeutics, Pliant, Regeneron, Rootpath, Rubius, Sapience, Simcha, Stcube, Sumitomo, Targovax, Teva, Turnstone, Verastem, Xilio. S.S.O. holds equity in CytoReason and serves as a consultant. Y.S. is a co-founder of OncoHost and RemedyCell, holds equity in these company and also serves as a consultant to both companies.

Received: August 19, 2022
Revised: June 2, 2023
Accepted: December 7, 2023
Published: January 4, 2024

REFERENCES

1. Schwaederle, M., Zhao, M., Lee, J.J., Lazar, V., Leyland-Jones, B., Schilsky, R.L., Mendelsohn, J., and Kurzrock, R. (2016). Association of Biomarker-Based Treatment Strategies With Response Rates and Progression-Free Survival in Refractory Malignant Neoplasms: A Meta-analysis. JAMA Oncol. *2*, 1452–1459.
2. Robert, C. (2020). A decade of immune-checkpoint inhibitors in cancer therapy. Nat. Commun. *11*, 3801.
3. Valero, C., Lee, M., Hoen, D., Zehir, A., Berger, M.F., Seshan, V.E., Chan, T.A., and Morris, L.G.T. (2021). Response Rates to Anti-PD-1

Immunotherapy in Microsatellite-Stable Solid Tumors With 10 or More Mutations per Megabase. JAMA Oncol. *7*, 739–743.

4. Lu, S., Stein, J.E., Rimm, D.L., Wang, D.W., Bell, J.M., Johnson, D.B., Sosman, J.A., Schalper, K.A., Anders, R.A., Wang, H., et al. (2019). Comparison of Biomarker Modalities for Predicting Response to PD-1/PD-L1 Checkpoint Blockade: A Systematic Review and Meta-analysis. JAMA Oncol. *5*, 1195–1204.
5. Rieth, J., and Subramanian, S. (2018). Mechanisms of Intrinsic Tumor Resistance to Immunotherapy. Int. J. Mol. Sci. *19*, 1340.
6. Brummel, K., Eerkens, A.L., de Bruyn, M., and Nijman, H.W. (2023). Tumour-infiltrating lymphocytes: from prognosis to treatment selection. Br. J. Cancer *128*, 451–458.
7. Filipovic, A., Miller, G., and Bolen, J. (2020). Progress Toward Identifying Exact Proxies for Predicting Response to Immunotherapies. Front. Cell Dev. Biol. *8*, 155.
8. Krieg, C., Nowicka, M., Guglietta, S., Schindler, S., Hartmann, F.J., Weber, L.M., Dummer, R., Robinson, M.D., Levesque, M.P., and Becher, B. (2018). High-dimensional single-cell analysis predicts response to anti-PD-1 immunotherapy. Nat. Med. *24*, 144–153.
9. Frangogiannis, N.G. (2012). Biomarkers: hopes and challenges in the path from discovery to clinical practice. Transl. Res. *159*, 197–204.
10. Taube, S.E., Clark, G.M., Dancey, J.E., McShane, L.M., Sigman, C.C., and Gutman, S.I. (2009). A perspective on challenges and issues in biomarker development and drug and biomarker codevelopment. J. Natl. Cancer Inst. *101*, 1453–1463.
11. Kuczynski, E.A., Krueger, J., Chow, A., Xu, P., Man, S., Sundaravadanam, Y., Miller, J.K., Krzyzanowski, P.M., and Kerbel, R.S. (2018). Impact of Chemical-Induced Mutational Load Increase on Immune Checkpoint Therapy in Poorly Responsive Murine Tumors. Mol. Cancer Therapeut. *17*, 869–882.
12. Nebot-Bral, L., Hollebecque, A., Yurchenko, A.A., de Forceville, L., Danjou, M., Jouniaux, J.M., Rosa, R.C.A., Pouvelle, C., Aoufouchi, S., Vuagnat, P., et al. (2022). Overcoming resistance to alphaPD-1 of MMR-deficient tumors with high tumor-induced neutrophils levels by combination of alphaCTLA-4 and alphaPD-1 blockers. J. Immunother. Cancer *10*, e005059.
13. Simoni, Y., Becht, E., Fehlings, M., Loh, C.Y., Koo, S.L., Teng, K.W.W., Yeong, J.P.S., Nahar, R., Zhang, T., Kared, H., et al. (2018). Bystander CD8(+) T cells are abundant and phenotypically distinct in human tumour infiltrates. Nature *557*, 575–579.
14. Weber, R., Fleming, V., Hu, X., Nagibin, V., Groth, C., Altevogt, P., Utikal, J., and Umansky, V. (2018). Myeloid-Derived Suppressor Cells Hinder the Anti-Cancer Activity of Immune Checkpoint Inhibitors. Front. Immunol. *9*, 1310.
15. Gabrilovich, D.I. (2017). Myeloid-Derived Suppressor Cells. Cancer Immunol. Res. *5*, 3–8.
16. Zingoni, A., Molfetta, R., Fionda, C., Soriani, A., Paolini, R., Cippitelli, M., Cerboni, C., and Santoni, A. (2018). NKG2D and Its Ligands: "One for All, All for One". Front. Immunol. *9*, 476.
17. Loeuillard, E., Yang, J., Buckarma, E., Wang, J., Liu, Y., Conboy, C., Pavelko, K.D., Li, Y., O'Brien, D., Wang, C., et al. (2020). Targeting tumor-associated macrophages and granulocytic myeloid-derived suppressor cells augments PD-1 blockade in cholangiocarcinoma. J. Clin. Invest. *130*, 5380–5396.
18. DeNardo, D.G., and Ruffell, B. (2019). Macrophages as regulators of tumour immunity and immunotherapy. Nat. Rev. Immunol. *19*, 369–382.
19. Gabrilovich, D.I., and Nagaraj, S. (2009). Myeloid-derived suppressor cells as regulators of the immune system. Nat. Rev. Immunol. *9*, 162–174.
20. Lawrence, S.M., Corriden, R., and Nizet, V. (2018). The Ontogeny of a Neutrophil: Mechanisms of Granulopoiesis and Homeostasis. Microbiol. Mol. Biol. Rev. *82*. e00057-17.
21. Alpert, A., Moore, L.S., Dubovik, T., and Shen-Orr, S.S. (2018). Alignment of single-cell trajectories to compare cellular expression dynamics. Nat. Methods *15*, 267–270.
22. Romero, D. (2019). Interferon enhances immune-checkpoint inhibition. Nat. Rev. Clin. Oncol. *16*, 6.
23. Mar, K.B., Rinkenberger, N.R., Boys, I.N., Eitson, J.L., McDougal, M.B., Richardson, R.B., and Schoggins, J.W. (2018). LY6E mediates an evolutionarily conserved enhancement of virus infection by targeting a late entry step. Nat. Commun. *9*, 3603.
24. Zhu, S., Zhang, T., Zheng, L., Liu, H., Song, W., Liu, D., Li, Z., and Pan, C.X. (2021). Combination strategies to maximize the benefits of cancer immunotherapy. J. Hematol. Oncol. *14*, 156.
25. Hong, C., Tijhuis, A.E., and Foijer, F. (2019). The cGAS Paradox: Contrasting Roles for cGAS-STING Pathway in Chromosomal Instability. Cells *8*.
26. Kwon, J., and Bakhoum, S.F. (2020). The Cytosolic DNA-Sensing cGAS-STING Pathway in Cancer. Cancer Discov. *10*, 26–39.
27. Caiazza, C., Brusco, T., D'Alessio, F., D'Agostino, M., Avagliano, A., Arcucci, A., Ambrosino, C., Fiume, G., and Mallardo, M. (2022). The Lack of STING Impairs the MHC-I Dependent Antigen Presentation and JAK/STAT Signaling in Murine Macrophages. Int. J. Mol. Sci. *23*, 14232.
28. Lee, J.J., Kim, S.Y., Kim, S.H., Choi, S., Lee, B., and Shin, J.S. (2022). STING mediates nuclear PD-L1 targeting-induced senescence in cancer cells. Cell Death Dis. *13*, 791.
29. Deguchi, A., Tomita, T., Ohto, U., Takemura, K., Kitao, A., Akashi-Takamura, S., Miyake, K., and Maru, Y. (2016). Eritoran inhibits S100A8-mediated TLR4/MD-2 activation and tumor growth by changing the immune microenvironment. Oncogene *35*, 1445–1456.
30. Lin, C.R., Wei, T.Y.W., Tsai, H.Y., Wu, Y.T., Wu, P.Y., and Chen, S.T. (2015). Glycosylation-dependent interaction between CD69 and S100A8/S100A9 complex is required for regulatory T-cell differentiation. Faseb. J. *29*, 5006–5017.
31. Zhang, Y., Lazarus, J., Steele, N.G., Yan, W., Lee, H.J., Nwosu, Z.C., Halbrook, C.J., Menjivar, R.E., Kemp, S.B., Sirihorachai, V.R., et al. (2020). Regulatory T-cell Depletion Alters the Tumor Microenvironment and Accelerates Pancreatic Carcinogenesis. Cancer Discov. *10*, 422–439.
32. Gungabeesoon, J., Gort-Freitas, N.A., Kiss, M., Bolli, E., Messemaker, M., Siwicki, M., Hicham, M., Bill, R., Koch, P., Cianciaruso, C., et al. (2023). A neutrophil response linked to tumor control in immunotherapy. Cell *186*, 1448–1464.e20.
33. Sharma, P., Hu-Lieskovan, S., Wargo, J.A., and Ribas, A. (2017). Primary, Adaptive, and Acquired Resistance to Cancer Immunotherapy. Cell *168*, 707–723.
34. Normand, R., Du, W., Briller, M., Gaujoux, R., Starosvetsky, E., Ziv-Kenet, A., Shalev-Malul, G., Tibshirani, R.J., and Shen-Orr, S.S. (2018). Found In Translation: a machine learning model for mouse-to-human inference. Nat. Methods *15*, 1067–1073.
35. Zilionis, R., Engblom, C., Pfirschke, C., Savova, V., Zemmour, D., Saatcioglu, H.D., Krishnan, I., Maroni, G., Meyerovitz, C.V., Kerwin, C.M., et al. (2019). Single-Cell Transcriptomics of Human and Mouse Lung Cancers Reveals Conserved Myeloid Populations across Individuals and Species. Immunity *50*, 1317–1334.e10.
36. Sagiv, J.Y., Voels, S., and Granot, Z. (2016). Isolation and Characterization of Low- vs. High-Density Neutrophils in Cancer. Methods Mol. Biol. *1458*, 179–193.
37. Chen, Z., Luo, Z., Zhang, D., Li, H., Liu, X., Zhu, K., Zhang, H., Wang, Z., Zhou, P., Ren, J., et al. (2023). TIGER: A Web Portal of Tumor Immunotherapy Gene Expression Resource. Dev. Reprod. Biol. *21*, 337–348.
38. Fehrenbacher, L., Spira, A., Ballinger, M., Kowanetz, M., Vansteenkiste, J., Mazieres, J., Park, K., Smith, D., Artal-Cortes, A., Lewanski, C., et al. (2016). Atezolizumab versus docetaxel for patients with previously treated non-small-cell lung cancer (POPLAR): a multicentre, open-label, phase 2 randomised controlled trial. Lancet *387*, 1837–1846.
39. Rittmeyer, A., Barlesi, F., Waterkamp, D., Park, K., Ciardiello, F., von Pawel, J., Gadgeel, S.M., Hida, T., Kowalski, D.M., Dols, M.C., et al. (2017). Atezolizumab versus docetaxel in patients with previously treated

non-small-cell lung cancer (OAK): a phase 3, open-label, multicentre randomised controlled trial. Lancet *389*, 255–265.

40. Zhang, Z., Wang, Z.X., Chen, Y.X., Wu, H.X., Yin, L., Zhao, Q., Luo, H.Y., Zeng, Z.L., Qiu, M.Z., and Xu, R.H. (2022). Integrated analysis of single-cell and bulk RNA sequencing data reveals a pan-cancer stemness signature predicting immunotherapy response. Genome Med. *14*, 45.
41. Ayers, M., Lunceford, J., Nebozhyn, M., Murphy, E., Loboda, A., Kaufman, D.R., Albright, A., Cheng, J.D., Kang, S.P., Shankaran, V., et al. (2017). IFN-gamma-related mRNA profile predicts clinical response to PD-1 blockade. J. Clin. Invest. *127*, 2930–2940.
42. Dou, A., and Fang, J. (2021). Heterogeneous Myeloid Cells in Tumors. Cancers *13*, 3772.
43. Yu, R., Zhu, B., and Chen, D. (2022). Type I interferon-mediated tumor immunity and its role in immunotherapy. Cell. Mol. Life Sci. *79*, 191.
44. Grasso, C.S., Tsoi, J., Onyshchenko, M., Abril-Rodriguez, G., Ross-Macdonald, P., Wind-Rotolo, M., Champhekar, A., Medina, E., Torrejon, D.Y., Shin, D.S., et al. (2020). Conserved Interferon-gamma Signaling Drives Clinical Response to Immune Checkpoint Blockade Therapy in Melanoma. Cancer Cell *38*, 500–515.e3.
45. Minn, A.J., and Wherry, E.J. (2016). Combination Cancer Therapies with Immune Checkpoint Blockade: Convergence on Interferon Signaling. Cell *165*, 272–275.
46. Johnson, D.B., Estrada, M.V., Salgado, R., Sanchez, V., Doxie, D.B., Opalenik, S.R., Vilgelm, A.E., Feld, E., Johnson, A.S., Greenplate, A.R., et al. (2016). Melanoma-specific MHC-II expression represents a tumour-autonomous phenotype and predicts response to anti-PD-1/PD-L1 therapy. Nat. Commun. *7*, 10582.
47. Pistillo, M.P., Carosio, R., Banelli, B., Morabito, A., Mastracci, L., Ferro, P., Varesano, S., Venè, R., Poggi, A., and Roncella, S. (2020). IFN-gamma upregulates membranous and soluble PD-L1 in mesothelioma cells: potential implications for the clinical response to PD-1/PD-L1 blockade. Cell. Mol. Immunol. *17*, 410–411.
48. Rodig, S.J., Gusenleitner, D., Jackson, D.G., Gjini, E., Giobbie-Hurder, A., Jin, C., Chang, H., Lovitch, S.B., Horak, C., Weber, J.S., et al. (2018). MHC proteins confer differential sensitivity to CTLA-4 and PD-1 blockade in untreated metastatic melanoma. Sci. Transl. Med. *10*, eaar3342.
49. Davar, D., Wang, H., Chauvin, J.M., Pagliano, O., Fourcade, J.J., Ka, M., Menna, C., Rose, A., Sander, C., Borhani, A.A., et al. (2018). Phase Ib/II Study of Pembrolizumab and Pegylated-Interferon Alfa-2b in Advanced Melanoma. J. Clin. Oncol. *36*, JCO1800632.
50. Yu, M., Peng, Z., Qin, M., Liu, Y., Wang, J., Zhang, C., Lin, J., Dong, T., Wang, L., Li, S., et al. (2021). Interferon-gamma induces tumor resistance to anti-PD-1 immunotherapy by promoting YAP phase separation. Mol. Cell *81*, 1216–1230.e9.
51. Gocher, A.M., Workman, C.J., and Vignali, D.A.A. (2022). Interferon-gamma: teammate or opponent in the tumour microenvironment? Nat. Rev. Immunol. *22*, 158–172.
52. Xue, D., Moon, B., Liao, J., Guo, J., Zou, Z., Han, Y., Cao, S., Wang, Y., Fu, Y.X., and Peng, H. (2022). A tumor-specific pro-IL-12 activates preexisting cytotoxic T cells to control established tumors. Sci. Immunol. *7*, eabi6899.
53. Bilusic, M., and Gulley, J.L. (2021). Neoadjuvant Immunotherapy: An Evolving Paradigm Shift? J. Natl. Cancer Inst. *113*, 799–800.
54. Jardim, D.L., Goodman, A., de Melo Gagliato, D., and Kurzrock, R. (2021). The Challenges of Tumor Mutational Burden as an Immunotherapy Biomarker. Cancer Cell *39*, 154–173.
55. Summers, C., Rankin, S.M., Condliffe, A.M., Singh, N., Peters, A.M., and Chilvers, E.R. (2010). Neutrophil kinetics in health and disease. Trends Immunol. *31*, 318–324.
56. Chevrier, S., Crowell, H.L., Zanotelli, V.R.T., Engler, S., Robinson, M.D., and Bodenmiller, B. (2018). Compensation of Signal Spillover in Suspension and Imaging Mass Cytometry. Cell Syst. *6*, 612–620.e5.
57. Weber, L.M., Nowicka, M., Soneson, C., and Robinson, M.D. (2019). diffcyt: Differential discovery in high-dimensional cytometry via high-resolution clustering. Commun. Biol. *2*, 183.
58. Hafemeister, C., and Satija, R. (2019). Normalization and variance stabilization of single-cell RNA-seq data using regularized negative binomial regression. Genome Biol. *20*, 296.
59. Hao, Y., Hao, S., Andersen-Nissen, E., Mauck, W.M., 3rd, Zheng, S., Butler, A., Lee, M.J., Wilk, A.J., Darby, C., Zager, M., et al. (2021). Integrated analysis of multimodal single-cell data. Cell *184*, 3573–3587.e29.
60. Aran, D., Looney, A.P., Liu, L., Wu, E., Fong, V., Hsu, A., Chak, S., Naikawadi, R.P., Wolters, P.J., Abate, A.R., et al. (2019). Reference-based analysis of lung single-cell sequencing reveals a transitional profibrotic macrophage. Nat. Immunol. *20*, 163–172.
61. Zhao, J., Jaffe, A., Li, H., Lindenbaum, O., Sefik, E., Jackson, R., Cheng, X., Flavell, R.A., and Kluger, Y. (2021). Detection of differentially abundant cell subpopulations in scRNA-seq data. Proc. Natl. Acad. Sci. USA *118*, e2100293118.
62. Saelens, W., Cannoodt, R., Todorov, H., and Saeys, Y. (2019). A comparison of single-cell trajectory inference methods. Nat. Biotechnol. *37*, 547–554.
63. Delile, J., Rayon, T., Melchionda, M., Edwards, A., Briscoe, J., and Sagner, A. (2019). Single cell transcriptomics reveals spatial and temporal dynamics of gene expression in the developing mouse spinal cord. Development *146*.
64. van Dijk, D., Sharma, R., Nainys, J., Yim, K., Kathail, P., Carr, A.J., Burdziak, C., Moon, K.R., Chaffer, C.L., Pattabiraman, D., et al. (2018). Recovering Gene Interactions from Single-Cell Data Using Data Diffusion. Cell *174*, 716–729.e27.
65. Finak, G., McDavid, A., Yajima, M., Deng, J., Gersuk, V., Shalek, A.K., Slichter, C.K., Miller, H.W., McElrath, M.J., Prlic, M., et al. (2015). MAST: a flexible statistical framework for assessing transcriptional changes and characterizing heterogeneity in single-cell RNA sequencing data. Genome Biol. *16*, 278.
66. La Manno, G., Soldatov, R., Zeisel, A., Braun, E., Hochgerner, H., Petukhov, V., Lidschreiber, K., Kastriti, M.E., Lönnerberg, P., Furlan, A., et al. (2018). RNA velocity of single cells. Nature *560*, 494–498.
67. Bergen, V., Lange, M., Peidli, S., Wolf, F.A., and Theis, F.J. (2020). Generalizing RNA velocity to transient cell states through dynamical modeling. Nat. Biotechnol. *38*, 1408–1414.
68. Wolf, F.A., Angerer, P., and Theis, F.J. (2018). SCANPY: large-scale single-cell gene expression data analysis. Genome Biol. *19*, 15.
69. Wolf, F.A., Hamey, F.K., Plass, M., Solana, J., Dahlin, J.S., Göttgens, B., Rajewsky, N., Simon, L., and Theis, F.J. (2019). PAGA: graph abstraction reconciles clustering with trajectory inference through a topology preserving map of single cells. Genome Biol. *20*, 59.
70. Van den Berge, K., Roux de Bézieux, H., Street, K., Saelens, W., Cannoodt, R., Saeys, Y., Dudoit, S., and Clement, L. (2020). Trajectory-based differential expression analysis for single-cell sequencing data. Nat. Commun. *11*, 1201.
71. Risso, D., Purvis, L., Fletcher, R.B., Das, D., Ngai, J., Dudoit, S., and Purdom, E. (2018). clusterExperiment and RSEC: A Bioconductor package and framework for clustering of single-cell and other large gene expression datasets. PLoS Comput. Biol. *14*, e1006378.
72. Yu, G., Wang, L.G., Han, Y., and He, Q.Y. (2012). clusterProfiler: an R package for comparing biological themes among gene clusters. OMICS *16*, 284–287.
73. Newman, A.M., Steen, C.B., Liu, C.L., Gentles, A.J., Chaudhuri, A.A., Scherer, F., Khodadoust, M.S., Esfahani, M.S., Luca, B.A., Steiner, D., et al. (2019). Determining cell type abundance and expression from bulk tissues with digital cytometry. Nat. Biotechnol. *37*, 773–782.
74. Hänzelmann, S., Castelo, R., and Guinney, J. (2013). GSVA: gene set variation analysis for microarray and RNA-seq data. BMC Bioinf. *14*, 7.
75. Liberzon, A., Birger, C., Thorvaldsdóttir, H., Ghandi, M., Mesirov, J.P., and Tamayo, P. (2015). The Molecular Signatures Database (MSigDB) hallmark gene set collection. Cell Syst. *1*, 417–425.

CellPress
OPEN ACCESS

76. Lee, S.H., Cho, S.Y., Yoon, Y., Park, C., Sohn, J., Jeong, J.J., Jeon, B.N., Jang, M., An, C., Lee, S., et al. (2021). Bifidobacterium bifidum strains synergize with immune checkpoint inhibitors to reduce tumour burden in mice. Nat. Microbiol. *6*, 277–288.

77. Timaner, M., Beyar-Katz, O., and Shaked, Y. (2016). Analysis of the Stromal Cellular Components of the Solid Tumor Microenvironment Using Flow Cytometry. Curr. Protoc. Cell Biol. *70*, 19.18.1–19.18.12.

78. Park, W., and Lopes, G. (2019). Perspectives: Neutrophil-to-lymphocyte Ratio as a Potential Biomarker in Immune Checkpoint Inhibitor for Non-Small-Cell Lung Cancer. Clin. Lung Cancer *20*, 143–147.

79. Shaked, Y., Pham, E., Hariharan, S., Magidey, K., Beyar-Katz, O., Xu, P., Man, S., Wu, F.T.H., Miller, V., Andrews, D., and Kerbel, R.S. (2016). Evidence Implicating Immunological Host Effects in the Efficacy of Metronomic Low-Dose Chemotherapy. Cancer Res. *76*, 5983–5993.

80. LaFleur, M.W., Nguyen, T.H., Coxe, M.A., Yates, K.B., Trombley, J.D., Weiss, S.A., Brown, F.D., Gillis, J.E., Coxe, D.J., Doench, J.G., et al. (2019). A CRISPR-Cas9 delivery system for in vivo screening of genes in the immune system. Nat. Commun. *10*, 1668.

STAR★METHODS

KEY RESOURCES TABLE

REAGENT or RESOURCE	SOURCE	IDENTIFIER
Antibodies		
Anti-mouse CD45- 115In (30-F11)	BioLegend	Cat# 103120, RRID: AB_312985
Anti-mouse CD274 (B7-H1, PD-L1)- 141Pr (10F.9G2)	BioLegend	Cat# 124302, RRID:AB_961226
Anti-mouse Ly-6G/Ly-6C (Gr-1)- 142Nd (RB6-8C5)	BioLegend	Cat# 108402, RRID: AB_313366
Anti-mouse IL-10r- 143Nd (1B1.3a)	BioLegend	Cat# 112708, RRID: AB_313521
Anti-mouse F4/80-144Nd (BM8)	BioLegend	Cat# 123143, RRID: AB_2563767
Anti-Mouse CD4-145Nd (RM4-5)	Fluidigm Corporation	Cat# 3145002B, RRID: AB_2687832
Anti-mouse/human CD45R/B220- 146Nd (RA3-6B2)	BioLegend	Cat# 103202, RRID: AB_312987
Anti-mouse Ly-6C- 147Sm (HK1.4)	BioLegend	Cat# 128002, RRID: AB_1134213
Anti-mouse CD8- 149Sm (53-6.7)	BioLegend	Cat# 100716, RRID: AB_312755
Anti-mouse CD335 (NKp46)- 150Nd (29A1.4)	BioLegend	Cat# 137625, RRID: AB_2563744
Anti-mouse CD206 (MMR)- 151Eu (C068C2)	BioLegend	Cat# 141702, RRID: AB_10900233
Anti-mouse CD25- 152Sm (3C7)	BioLegend	Cat# 101913, RRID: AB_2562798
Anti-mouse IL-6r (CD126)- 153Eu (D7715A7)	BioLegend	Cat# 115808, RRID: AB_313679
Anti-mouse CD11c (N418)- 154Sm (N418)	BioLegend	Cat# 117302, RRID: AB_313770
Anti-mouse CCR9- 155Gd (9B1)	BioLegend	Cat# 129704, RRID: AB_1227487
Anti-mouse CD49b- 156Gd (HMα2)	BioLegend	Cat# 103513, RRID: AB_2563754
Anti-mouse CD19- 157Gd (6D5)	BioLegend	Cat# 115502, RRID: AB_313636
Anti-mouse CD279 (PD-1)- 158Gd (RMP1-30)	BioLegend	Cat# 109113, RRID: AB_2563735
Anti-mouse CD27- 159Tb (LG.3A10)	BioLegend	Cat# 124202, RRID: AB_1236456
Anti-mouse CD69- 160Gd (H1.2F3)	BioLegend	Cat# 104502, RRID: AB_313105
Anti-mouse CD150- 161Dy (SLAM) (459911)	R&D Systems	Cat# MAB4330, RRID: AB_1208051
Anti-Mouse CD31- 162Dy (MEC 13.3)	BD Biosciences	Cat# 553370, RRID: AB_394816
Anti-mouse CD127(IL-7Rα)- 163Dy (A7R34)	BioLegend	Cat# 135002, RRID: AB_1937287
Anti-mouse CD28-164Dy (37.51)	BioLegend	Cat# 102102, RRID: AB_312866
Anti-mouse CD115 (CSF-1R)- 165Ho (AFS98)	BioLegend	Cat# 135502, RRID: AB_1937292
Anti-mouse CD93 (C1qR1)- 167Er (223437)	R&D Systems	Cat# MAB1696, RRID: AB_2076062
Anti-mouse CD117 (c-Kit)- 168Er (2B8)	BioLegend	Cat# 105802, RRID: AB_313210
Anti-mouse CD365 (Tim-1)- 169Tm (RMT1-4)	BioLegend	Cat# 119502, RRID: AB_345368
Anti-mouse CD62L- 170Er (MEL-14)	BioLegend	Cat# 104402, RRID: AB_313089
Anti-mouse CD44- 171Yb (IM7)	BioLegend	Cat# 103014, RRID: AB_312965
Anti-mouse CD23- 172Yb (B3B4)	BioLegend	Cat# 101625, RRID: AB_2563731
Anti-mouse Ly-6A/E (Sca-1)- 173Yb (D7)	BioLegend	Cat# 108102, RRID: AB_313339
Anti-mouse CD309 (VEGFR2, Flk-1)- 174Yb (89B3A5)	BioLegend	Cat# 121902, RRID: AB_756162
Anti-mouse CD5- 175Lu (53-7.3)	BioLegend	Cat# 100602, RRID: AB_312731
Anti-mouse CD11b- 176Yb (M1/70)	BioLegend	Cat# 101249, RRID: AB_2562797
Alexa Fluor® 700 anti-mouse CD45 (30-F11)	BioLegend	Cat# 103127, RRID: AB_493715
PerCP anti-mouse/human CD11b (M1/70)	BioLegend	Cat# 101229, RRID: AB_2129375
PE/Cyanine7 anti-mouse Ly-6C (HK1.4)	BioLegend	Cat# 128017, RRID: AB_1732093
Brilliant Violet 510™ anti-mouse Ly-6G (1A8)	BioLegend	Cat# 127633, RRID: AB_2562937
Goat Anti-LY6E (polyclonal)	Novusbio	Cat# NBP1-52176
Alexa Fluor® 488 AffiniPure Donkey Anti-Goat IgG (H+L)	JacksonImmunoResearch	Cat# 705-545-147, RRID: AB_2336933
APC/Cyanine7 anti-mouse CD8a Antibody (53-6.7)	BioLegend	Cat# 100714, RRID: AB_312753
APC anti-mouse CD25 Antibody (PC61)	BioLegend	Cat# 102012, RRID: AB_312860

(*Continued on next page*)

Continued

REAGENT or RESOURCE	SOURCE	IDENTIFIER
Brilliant Violet 421™ anti-mouse CD107a (LAMP-1) Antibody (1D4B)	BioLegend	Cat# 121617, RRID: AB_2749905
APC/Cyanine7 anti-mouse CD11c Antibody (N418)	BioLegend	Cat# 117323, RRID: AB_830646
PE anti-mouse F4/80 Antibody (BM8)	BioLegend	Cat# 123110, RRID: AB_893498
Brilliant Violet 421™ anti-mouse CD206 (MMR) Antibody (C068C2)	BioLegend	Cat# 141717, RRID: AB_2562232
Brilliant Violet 605™ anti-mouse/human CD45R/B220 Antibody (RA3-6B2)	BioLegend	Cat# 103243, RRID: AB_2563312
Brilliant Violet 510™ anti-mouse CD4 Antibody (GK1.5)	BioLegend	Cat# 100449, RRID: AB_2564587
PE anti-mouse CD25 Antibody (PC61)	BioLegend	Cat# 102007, RRID: AB_312857
Brilliant Violet 421™ anti-mouse H-2Kd Antibody (SF1-1.1)	BioLegend	Cat# 116623, RRID: AB_2565656
APC anti-mouse CD274 (B7-H1, PD-L1) Antibody (10F.9G2)	BioLegend	Cat# 124312, RRID: AB_10612741
PE anti-mouse IFN-γ Antibody (XMG1.2)	BioLegend	Cat# 505807, RRID: AB_315401
APC anti-mouse IFNAR-1 Antibody (MAR1-5A3)	BioLegend	Cat# 127313, RRID: AB_2122746
PE Anti-Mouse IFN-gamma-R-alpha CD119 Antibody (GR-20)	Elabscience	Cat# E-AB-F1115D
Brilliant Violet 421™ anti-mouse/human CD44 Antibody (IM7)	BioLegend	Cat# 103040, RRID: AB_10895752
PE/Cyanine7 anti-mouse CD62L Antibody (MEL-14)	BioLegend	Cat# 104418, RRID: AB_313103
FITC anti-human/mouse Granzyme B Recombinant Antibody (QA16A02)	BioLegend	Cat# 372206, RRID: AB_2687029
PE anti-mouse Ki-67 Antibody (16A8)	BioLegend	Cat# 652404, RRID: AB_2561524
APC anti-human/mouse Granzyme B Recombinant Antibody (QA16A02)	BioLegend	Cat# 372204, RRID: AB_2687027
APC/Cyanine7 anti-human CD45 Antibody (HI30)	BioLegend	Cat# 304014, RRID: AB_314402
PerCP anti-human HLA-DR Antibody (L243)	BioLegend	Cat# 307628, RRID: AB_893574
Brilliant Violet 510™ anti-human Lineage Cocktail (CD3, CD14, CD16, CD19, CD20, CD56) (OKT3; M5E2; 3G8; HIB19; 2H7; HCD56)	BioLegend	Cat# 348807
Brilliant Violet 605™ anti-human CD11b Antibody (ICRF44)	BioLegend	Cat# 301332, RRID: AB_2562020
PE/Cyanine7 anti-human CD33 Antibody (WM53)	BioLegend	Cat# 303434, RRID: AB_2734264
APC anti-human CD14 Antibody (63D3)	BioLegend	Cat# 367118, RRID: AB_2566791
FITC anti-human CD15 (SSEA-1) Antibody (HI98)	BioLegend	Cat# 301904, RRID: AB_314196
Recombinant Anti-human LY6E Antibody-Pe conjugated	Creative Biolabs	Cat# MOB-636-PE
InVivoMAb anti-mouse PD-1 (CD279) (RMP1-14)	BioXCell	Cat# BE0146, RRID: AB_10949053
Anti-PD-1 (RMP1-14)	ichorbio	Cat# ICH1132, RRID: AB_2921498
InVivoMAb rat IgG2a isotype control, anti-trinitrophenol (2A3)	BioXCell	Cat# BE0089, RRID: AB_1107769
Rat IgG2a *In Vivo* Isotype Control – Low Endotoxin (1-1)	ichorbio	Cat# ICH2244, RRID: AB_2921379
InVivoMAb anti-mouse IFNAR-1 (MAR1-5A3)	BioXCell	Cat# BE0241, RRID: AB_2687723
InVivoMAb anti-mouse IFNγR (CD119)	BioXCell	Cat# BE0029, RRID: AB_1107576
PE anti-mouse Ly-6G/Ly-6C (Gr-1) Antibody (RB6-8C5)	BioLegend	Cat# 108408, RRID: AB_313373
Mouse IL-12/IL-23 p40 Antibody	R&D Systems	Cat# MAB4991, RRID: AB_2123749
Mouse IL-23 p19 Antibody	R&D Systems	Cat# AF1619, RRID: AB_354897
Anti- ds DNA antibody (35I9 DNA)	abcam	Cat# ab27156, RRID: AB_470907
ISG15 Antibody (F-9)	Santa Cruz Biotechnology	Cat# sc-166755, RRID: AB_2126308
IRF3 (D83B9) Rabbit mAb	Cell signaling	Cat# 4302S, RRID: AB_1904036

(*Continued on next page*)

Continued

REAGENT or RESOURCE	SOURCE	IDENTIFIER
NF-kB (D14E12) Rabbit mAb	Cell signaling	Cat# 8242S, RRID: AB_10859369
STING (D2P2F) Rabbit mAb	Cell signaling	Cat# 13647S, RRID: AB_2732796
Anti-Mouse IFN-Beta (neutralizing antibody, Rabbit IgG)	PBL assay science	Cat# 32401-1, RRID: AB_10891517
Biological samples		
Human blood samples	Sheba medical center, Tel Hashomer (Ramat Gan, Israel)	0226-13
Human blood samples	Yale University School of Medicine (New Haven, CT, USA)	0609001869
Human blood samples	Rambam Heath Care campus (Haifa, Israel), Israel National Biobank for Research (Midgam)	RMB-0631-17
Human blood samples	Hadassah Medical Center (Jerusalem, Israel), Israel National Biobank for Research (Midgam)	RMB-0631-17
Chemicals, peptides, and recombinant proteins		
Dulbecco's Modified Eagle's Medium - high glucose	Sigma	Cat# D5796
Fetal Bovine Serum	Biological Industries	Cat# 10270-106
DPBS, no calcium, no magnesium	Biological Industries	Cat# 02-023-1A
StemSpam SFEM II media	StemCell	Cat# 09605
1-methyl-3-nitro-1-nitrosoguanidine	Apollo Scientific	Cat# OR301388
Propidium Iodide Solution	BioLegend	Cat# 421301
STING inhibitor (H151)	Cayman chemical	Cat# 25857
Fix/Perm Buffer (4x)	BioLegend	Cat# 421401
Perm Buffer (10x)	BioLegend	Cat# 421402
Recombinant Mouse IFN-α (carrier-free)	BioLegend	Cat# 752802
Recombinant Murine IFN-γ	Peprotech	Cat# 315-05
cOmplete™ Protease Inhibitor Cocktail	Roche	Cat# 11697498001
PhosSTOP	Roche	Cat# 4906845001
Critical commercial assays		
IFN alpha Mouse ELISA Kit	Invitrogen	Cat# BMS6027
Mouse Granzyme B DuoSet ELISA	R&D Systems	Cat# DY1865-05
LEGENDplex™ MU Th1/Th2 Panel (8-plex) w/ FP V03	BioLegend	Cat# 741053
Proteome Profiler Mouse XL Cytokine Array	R&D Systems	Cat# ARY028
EasySep™ Mouse PE Positive Selection Kit II	Stemcell Technologies	Cat# 17666
Live Cell Labeling Kit - Red Fluorescence - Cytopainter	abcam	Cat# ab187965
Total RNA Purification Micro Kit	Norgen	Cat# 35300
High-Capacity cDNA Reverse Transcription Kit	Applied Biosystems	Cat# 4374966
Fast SYBR™ Green Master Mix	Bio-Rad	Cat# 4385612
MojoSort™ Mouse CD8 T Cell Isolation Kit	BioLegend	Cat# 480008
MagCellect Mouse Hematopoietic Cell Lineage Depletion Kit	R&D Systems	Cat# MAGM209
ELISA Mouse IL-6	BioLegend	Cat# 431301

(*Continued on next page*)

Continued

REAGENT or RESOURCE	SOURCE	IDENTIFIER
**Deposited data**		
Single cell RNA sequencing of GR1^{+} cells	This paper	GEO: GSE226962
CyTOF data	This paper	https://github.com/ShakedLab/Ly6E_Biomarker
Bulk RNA-seq data (BLCA)	https://doi.org/10.1186/s13073-022-01050-w	EGAS00001002556
Bulk RNA-seq data (GBM, RCC, SKCM – Gide, SKCM – Hugo, SKCM – Allen, SKCM – Riaz, STAD)	https://doi.org/10.1016/j.gpb.2022.08.004	http://tiger.canceromics.org/
Bulk RNA-seq data (NSCLC – OAK and POPLAR)	https://doi.org/10.1016/S0140-6736(16)00587-0. https://doi.org/10.1016/S0140-6736(16)32517-X.	EGAD00001007703
Bulk RNA-seq data (SKCM – Liu)	https://doi.org/10.1186/s13073-022-01050-w	phs000452
**Experimental models: Cell lines**		
4T1	ATCC	CRL-25390
EMT6	ATCC	CRL-2755
LLC	ATCC	CRL-1642
RENCA	ATCC	CRL-2947
HEK-293FT	Cellosaurus	CVCL-6911
4T1 mutagenized	This paper	N/A
LLC mutagenized	This paper	N/A
RENCA mutagenized	This paper	N/A
**Experimental models: Organisms/strains**		
BALB/c mice	Envigo	Cat# 162
C57BL/6 mice	Envigo	Cat# 057
CBA/JCrHsd mice	Envigo	Cat# 055
SCID mice	Envigo	Cat# 182
Constitutive Cas9-expressing mice	JAX mice	Cat# 026179
C57BL/6 x CBA backcrossed mice	This paper	N/A
**Oligonucleotides**		
mTNFα<br>F: CTGAACTTCGGGGTGATCGG<br>R: GGCTTGTCACTCGAATTTTGAGA	Sigma-Aldrich	N/A
mCXCL1<br>F: CTGGGATTCACCTCAAGAACATC<br>R: CAGGGTCAAGGCAAGCCTC	Sigma-Aldrich	N/A
mIL1α<br>F: TCTCAGATTCACAACTGTTCGTG<br>R: AGAAAATGAGGTCGGTCTCACTA	Sigma-Aldrich	N/A
mIL23a<br>F: CAGCAGCTCTCTCGGAATCTC<br>R: TGGATACGGGGCACATTATTTTT	Sigma-Aldrich	N/A
mSaa3<br>F: TGCCATCATTCTTTGCATCTTGA<br>R: CCGTGAACTTCTGAACAGCCT	Sigma-Aldrich	N/A
mCCL3<br>F: TGTACCATGACACTCTGCAAC<br>R: CAACGATGAATTGGCGTGGAA	Sigma-Aldrich	N/A
mCCL6<br>F: AAGAAGATCGTCGCTATAACCCT<br>R: GCTTAGGCACCTCTGAACTCTC	Sigma-Aldrich	N/A

(*Continued on next page*)

Continued		
REAGENT or RESOURCE	SOURCE	IDENTIFIER
Ly6E gRNA forward: 5'CACCG AGCA AGCTAAGCCTGCGCAC 3'	Sigma-Aldrich	N/A
Ly6E gRNA reverse: 5'AAAC GTGCGC AGGCTTAGCTTGCT C 3'	Sigma-Aldrich	N/A
Recombinant DNA		
pXPR_053 plasmid	Addgene	Addgene Plasmid # 113591, RRID: Addgene_113591
psPAX2 plasmid	Addgene	Addgene Plasmid #12259, RRID: DGRC_12259
pMD2.G (VSV-G) plasmid	Addgene	Addgene Plasmid #12259, RRID: DGRC_12259
Software and algorithms		
FlowJo V.10	BD Bioscience	https://www.flowjo.com/
Legendplex v8.0	BioLegend	https://www.biolegend.com/
LAS-4000	Fujifilm	https://www.fujifilm.com/
ImageJ v1.53	ImageJ	https://imagej.net/
R (v4.1.0)	R Core Team	https://www.python.org
Python (v3.8.5)	Python Software Foundation	https://www.r-project.org/
CellRanger (v5.0.1)	10X Genomics	https://www.10xgenomics.com/
CATALYST (v1.24.0)	https://doi.org/10.1016/j.cels.2018.02.010	https://bioconductor.org/packages/release/bioc/html/CATALYST.html
diffcyt (v1.20.0)	https://doi.org/10.1038/s42003-019-0415-5	https://bioconductor.org/packages/release/bioc/html/diffcyt.html
SCTransform (v0.3.2)	https://doi.org/10.1186/s13059-019-1874-1	https://github.com/satijalab/sctransform
Seurat (v4.0.3)	https://doi.org/10.1016/j.cell.2021.04.048	https://satijalab.org/seurat/
SingleR (v1.6.1)	https://doi.org/10.1038/s41590-018-0276-y	https://bioconductor.org/packages/release/bioc/html/SingleR.html
celldex (v1.2.0)	https://doi.org/10.1038/s41590-018-0276-y	https://bioconductor.org/packages/release/data/experiment/html/celldex.html
DASeq (v1.0.0)	https://doi.org/10.1073/pnas.2100293118	https://github.com/KlugerLab/DAseq
dynplot (v1.1.1)	https://doi.org/10.1038/s41587-019-0071-9	https://dynverse.org/
schex (v1.6.3)	https://doi.org/10.1242/dev.173807	https://bioconductor.org/packages/release/bioc/html/schex.html
MAGIC (v2.0.3)	https://doi.org/10.1016/j.cell.2018.05.061	https://github.com/KrishnaswamyLab/MAGIC
MAST (v1.18.0)	https://doi.org/10.1186/s13059-015-0844-5	https://bioconductor.org/packages/release/bioc/html/MAST.html
velocyto (v0.17)	https://doi.org/10.1038/s41586-018-0414-6	https://velocyto.org/
scvelo (v0.2.4)	https://doi.org/10.1038/s41587-020-0591-3	https://scvelo.readthedocs.io/en/stable/
scanpy/PAGA (v1.8.0)	https://doi.org/10.1186/s13059-017-1382-0; https://doi.org/10.1186/s13059-019-1663-x	https://scanpy.readthedocs.io/en/stable/
tradeSeq (v1.6.0)	https://doi.org/10.1038/s41467-020-14766-3	https://bioconductor.org/packages/release/bioc/html/tradeSeq.html

(*Continued on next page*)

Continued

REAGENT or RESOURCE	SOURCE	IDENTIFIER
clusterExperiment (v2.12.0)	https://doi.org/10.1371/journal.pcbi.1006378	https://bioconductor.org/packages/release/bioc/html/clusterExperiment.html
clusterProfiler (v4.0.0)	https://doi.org/10.1371/journal.pcbi.1006378	https://bioconductor.org/packages/release/bioc/html/clusterProfiler.html
cellAlign (v0.1.0)	https://doi.org/10.1038/nmeth.4628	https://github.com/shenorrLabTRDF/cellAlign
CIBERSORTx	https://doi.org/10.1038/s41587-019-0114-2	https://cibersortx.stanford.edu/
GSVA (v1.47.3)	https://doi.org/10.1186/1471-2105-14-7	https://bioconductor.org/packages/release/bioc/html/GSVA.html
msigdbr (v7.4.1)	https://doi.org/10.1016/j.cels.2015.12.004	https://www.gsea-msigdb.org/gsea/msigdb

RESOURCE AVAILABILITY

Lead contact

Further information and requests for resources and reagents should be directed to and will be fulfilled by the lead contact, Yuval Shaked (yshaked@technion.ac.il)

Materials availability

All unique reagents generated in this study are available from the lead contact without restriction, unless commercially available.

Data and code availability

- Raw sequencing, single-cell RNA-seq (scRNA-seq) and RNA-velocity data have been deposited in GEO and are publicly available as of the date of publication. Accession numbers are listed in the key resources table. Processed CYTOF and scRNA-seq data are additionally available as of the date of publication at GitHub: (https://github.com/ShakedLab/Ly6E_Biomarker). This paper analyzes existing, publicly available data. The accession numbers for these datasets are listed in the key resources table.
- This paper does not report original code.
- Any additional information required to reanalyze the data reported in this paper is available from the lead contact upon request.

EXPERIMENTAL MODEL AND SUBJECT DETAILS

The establishment of diverse models to study predictive biomarkers for immunotherapy

One of the major obstacles in immuno-oncology is the use of mouse models to study immunotherapy. Here we used several models to search and validate biomarkers for ICI therapy (Figure 1). In this approach we used the 4T1 tumor model in which cells were mutagenized as indicated below to generate clones responsive and resistant to immunotherapy. Subsequently, tumors or blood was harvested at baseline (the pre-treatment stage), and subjected to high resolution single cell assays (e.g., single cell RNA sequencing [scRNA-seq] or mass cytometry [CyTOF]) to identify cell states that differentiate between eventual responders and non-responders (Figure 1A). Additional studies were performed to analyze the mode of action of this specific cellular biomarker. Subsequently, we validated this potential cellular biomarker in other models – establishing its ability to predict immunotherapy response in mice regardless of the underlying mechanism(s) (Figure 1B). Specifically, we used multiple cancer types (breast, lung, renal cancers), three different mouse strains (BALB/c, C57BL/6 and C57BL/6 x CBA backcrossed), and multiple clones of the same tumor cell lines (4T1 murine breast carcinoma, LLC lung carcinoma and RENCA renal cell carcinoma, all parental clones were obtained from the ATCC) (Figure 1B and Table S3).

Mutagenized model

We generated cell line pairs comprising a clone that is responsive to anti-PD1 from a non-responsive parental cell line. The responsive clones were generated through mutagenesis (see below), therefore mimicking mutational load – a clinically relevant metric for immunotherapy response. This process provides pairs of cells originating from the same cell line, allowing a biologically relevant comparison.

Spontaneous model

We used a tumor cell line that displays a natural, spontaneous response to anti-PD1 (EMT6 cell line). This model mimics a host dependent mechanism of response to immunotherapy.

Backcrossed model

We generated a mixed background strain. Specifically, C57BL mice were bred with CBA mice to create an F1 generation. F1 progeny are unable to grow syngeneic C57BL/6 tumors. We therefore backcrossed with inbred C57BL/6 mice for 5 generations, as opposed

to the standard 10 generations. The resulting mice are compatible with C57BL/6 syngeneic cell lines, but retain enough heterogeneity to drive a variable host-dependent response to anti-PD1 (See Figure S9E).

Clinical translation

To translate the use of this cellular biomarker into humans, a functionally equivalent cell state can be identified through public data mining (Figure 1C). Functional equivalence is superior to the use of direct orthologues (e.g. "Gene-A" in both mice and humans) as they may not necessarily mark the same cell state in a different species. Here, we analyzed published scRNA-seq data from the blood of patients with non-small cell lung carcinoma (NSCLC) to identify cells undergoing similar biological processes to the cells identified in mice. Subsequently, the cellular biomarker was validated in a separate retrospective cohort of patients with NSCLC and melanoma treated with ICI-based therapy, as well as in publicly available datasets of additional tumor types, as outlined below.

Cell lines and culture

4T1, EMT6 (murine breast carcinoma cell lines), RENCA (murine renal carcinoma), and LLC (murine Lewis lung carcinoma) were purchased from the American Type Culture Collection (Manassas, VA, USA) and were used within 6 months of thawing. Cells were routinely tested to be mycoplasma-free. All the cell lines were maintained under 37°C and 5% CO_2 conditions in Dulbecco's modified Eagle's medium (DMEM, Sigma-Aldrich, Rehovot, Israel, Cat# D5796) supplemented with 10% fetal calf serum (FCS, Biological Industries, Israel, Cat# 10270-106), 1% L-glutamine (Cat# 03-020-1B), 1% sodium pyruvate (Cat# S8636), and 1% Pen-Strep-Neomycin (Cat# 03-034-1B) in solution (Biological Industries, Israel).

Mouse tumor models

The use of animals and experimental protocols were approved by the Animal Care and Use Committee of the Technion. Female BALB/c, C57BL/6, and combined immunodeficient (SCID) mice (8 weeks of age) were purchased from Envigo, Israel. Mixed background mice were created by backcrossing female C57BL/6 and CBA female mice with pure C57BL/6 male mice for 5 generations. All mice were maintained under specific pathogen-free conditions in the animal facility. $4T1_P$, $4T1_M$ and EMT6 ($5x10^5$/50μL in serum free medium) were orthotopically injected into the mammary fat pad of 8–10-week-old female BALB/c mice or SCID mice. $RENCA_P$, $RENCA_M$, LLC_P and LLC_M ($5x10^5$/50μL in serum free medium) were subcutaneously injected into the flanks of 8–10-week-old female BALB/c and C57BL/6 mice, respectively. Mice were randomly grouped before therapy. Typically, the number of mice per group was set to 5, to reach statistical power, unless indicated otherwise in the text. In all experiments, when tumors reached ~50 mm^3 mice were treated with anti-mouse anti-PD-1 (clone RMP1-14, BioXCell Cat# BE0146 or ichorbio Cat# ICH1132) antibody. The antibody was given twice a week in a dose of 100μg/mouse for up to 2-week period. The control groups were injected with IgG isotype control (BioXCell Cat# BE0089 or ichorbio Cat# ICH2244). In some experiments mice were treated with the combination of IFNα (BioLegend, Cat# 752802) and IFNγ (Peprotech, Israel, Cat# 315-05) in a total dose of 2μg/mouse for 10 days or with antibodies blocking IFN-Rα (Clone MAR1-5A3,BioXCell, Cat# BE0241) and IFN-Rγ (Clone GR-20, BioXCell, Cat# BE0029), at a dose of 50μg/mouse twice a week, as previously described.[76]

Blood collection from patients with cancer

Blood collection from human subjects was approved by ethic committees at Sheba medical center, Tel Hashomer (Ramat Gan, Israel) (IRB: 0226-13), Yale University School of Medicine (New Haven, CT, USA) (IRB: 0609001869) as well as Rambam Heath Care campus (Haifa, Israel) and Hadassah Medical Center (Jerusalem, Israel) through the national bio-bank (Midgam, Israel) (IRB: RMB-0631-17). All patients signed informed consent. Blood was drawn at baseline, before immunotherapy, from patients with non-small cell lung cancer (n=50) and melanoma (n=59). Patient characteristics are indicated in Table S2. Peripheral blood mononuclear cells (PBMCs) were isolated from ficoll tubes and stored in freezing medium at -80°C, until further analyzed. PBMCs were then thawed and analyzed by flow cytometry using a mixture of antibodies indicated above. Patients were stratified to responders and non-responders based on RECIST criteria at 3 and/or 6 months where partial/complete response and stable disease patients were considered responders, and progressive disease patients were considered non-responders. The correlations of $Ly6E^{hi}$ neutrophil levels with response rates were then calculated.

METHODS DETAILS

Cell line mutagenesis

Parental cell lines that are resistant to ICI therapy were cultured with 1-methyl-3-nitro-1-nitrosoguanidine (MNNG, Apollo Scientific, Cat# OR301388) for 2 hours. After the cells were washed with PBS and growth medium was added, cells were allowed to recover over 5 days and multiclonal mutational cells were created. Mutagenized cells were validated *in-vivo* for their response to ICI therapy. Using this procedure, we have generated responsive clones to ICI therapy including 4T1 parental cells ($4T1_P$) and its mutagenized clone ($4T1_M$), LLC parental cell line (LLC_P) and its mutagenized clone (LLC_M), and RENCA parental cell line ($RENCA_P$) and its mutagenized clone ($RENCA_M$). 4T1 tumors were also evaluated for immunogenicity as described in Figure 2.

Tumor lysate preparation and protein measurement

$4T1_P$ and $4T1_M$ tumor tissues were placed in a 1.6 mL tube containing RIPA buffer (5M NaCl (Fisher Chemical, Cat# 231-598-3), 0.5M EDTA (Sigma-Aldrich, Cat# EDS) pH=8, 1M Tris (Alfa Aesar, Cat# A12274) pH=8, 1% NP-40, 10% sodium deoxycholate

(Sigma-Aldrich, Cat#D6750), 10% SDS (Fisher Scientific, Cat# BP2436-1), protease inhibitor cocktail (1:100, Roche, Cat# 11697498001) and phosphatase inhibitor (1:20 PhosSTOP, Roche, Cat# 4906845001). Stainless steel beads (Cat# SSB14B, Next Advance, New York, USA) were added and tumor tissue was homogenized using the Bullet Blender Tissue Homogenizer (Next Advance) according to the manufacturer's protocol. The homogenate was centrifuged and supernatant was collected. The protein concentration of the tumor lysates was determined using Protein Assay Dye Reagent Concentrate (Bio-Rad, California, USA, Cat# 500-0006). The quantification of IFNγ and TNFα was carried out by using the LEGENDplex Mouse Th1/Th2 Panel (BioLegend, San Diego, CA, USA, Cat# 741053), in accordance with the manufacturer's instructions. In addition, IFNα (Invitrogen, Cat# BMS6027) and Granzyme B (R&D Systems, Minneapolis, MN, USA, Cat# DY1865-05) were quantified by specific ELISA according to the manufacturer's instructions. All experiments were performed using at least three biological repeats.

Cytokine array and biological pathway enrichment

$GR1^+$ cells or IFN-induced $Ly6E^{hi}$ neutrophils were cultured in serum-free medium for 24 hours to generate conditioned medium (10^6 cells/ml). The conditioned medium was applied to a proteomic profiler mouse XL cytokine array (R&D, MN, Cat# ARY028), in accordance with the manufacturer's instructions. Relative levels of the different proteins were calculated based on densitometry and compared between $GR1^+$ and $Ly6E^{hi}$ neutrophils to obtain $\log_2$(fold changes). Over-representation tests were performed using *clusterProfiler* [v4.0.0][72] and gene-lists from the Gene Ontology (GO) database to characterize all differentially expressed proteins with an absolute $\log_2FC > 0.35$. Only significantly enriched (FDR < 0.01, Bonferroni correction method) pathways were retained.

Flow cytometry acquisition and analysis

Validation of cell subpopulations in tumors and peripheral blood was carried out as follows. Cells from tumors after the tumor underwent single cell suspension as previously described[77] or peripheral blood after the samples underwent red blood cell lysis, were immunostained for the following surface markers: Murine and human granulocytic population were defined as $CD45^+/CD11b^+/Ly6C^{Lo}Ly6G^+$ and $CD45^+/Lin^-HLA\text{-}DR^-/CD33^+CD11b^+/$ $CD14^-CD15^+$, respectively, as previously described.[18,78] Figure S3 represents the gating strategy for the detection of $Ly6E^{hi}$ neutrophils in mouse and human. In addition, immune cells were defined based on the following surface markers: B cells, ($CD45^+/B220^+$), activated cytotoxic T cells, ($CD45^+/CD8^+/CD25^+$), T helper cells ($CD45^+/CD4^+$), monocytes ($CD45^+/CD11b^+/Ly6C^+/Ly6G^{lo}$) and M1 macrophages ($CD45^+/CD11b^+/F4/80^+/CD206^-/CD11c^+$), M2 macrophages ($CD45^+/CD11b^+/F4/80^+/CD206^+/CD11c^-$). In some experiments, IFNγ, IFN-Rα, and IFN-Rγ were evaluated by flow cytometry, after cell permeabilization was carried out, when required. All monoclonal antibodies were purchased from BD Biosciences, BioLegend, R&D systems, Militenyi Biotec, and Elabscience. Ly6E antibodies for mouse and human were purchased from Novusbio, Novus Biologicals, CO, USA, and Creative Biolabs, NY, USA, respectively. All antibodies were used in accordance with the manufacturer's instructions. At least 300,000 events were acquired using a BD LSRFortessa flow cytometer and analyzed with FlowJo V.10 software (FlowJo, Ashland, Oregon, USA).

Time of flight mass cytometry (CyTOF)

$4T1_P$ and $4T1_M$ ($5x10^5/50\mu L$ in serum free medium) were orthotopically injected into the mammary fat pad of 8–10-week-old female BALB/c mice (n=5 mice/group). When tumors reached ~50 mm^3, mice were treated with anti-mouse anti-PD-1 or IgG control for 2 weeks, as described above. At endpoint, mice were sacrificed and tumors were prepared as single cell suspensions. The cells were acquired by CyTOF as previously described.[79] Briefly, an equal number of tumor cells were pooled per group (5 mice/group) and $3x10^6$ cells were collected from each pool for CyTOF acquisition. The cells were washed with cell staining media (PBS without Ca^{2+}/Mg^{2+}, 2% bovine serum albumin, and 0.09% Azide) and immunostained with a mix of metal tagged antibodies (See: key resources table). Following acquisition, the cells were gated and analyzed, as described below.

Adoptive transfer of $Ly6E^{hi}$ neutrophils experiments

$GR1^+$ cells were isolated (positive isolation, EasySep Mouse PE, Stemcell Technologies, Cat# 17666) from the spleens of 4T1 tumor bearing mice and cultured overnight with 5% medium containing IFNα and IFNγ (10 ng/ml each, BioLegend Cat# 752802 and Peprotech Cat# 315-05). Subsequently, cells were collected, centrifuged and washed twice with PBS. $Ly6E^{hi}$ neutrophils were analyzed by flow cytometry and by RT-qPCR as described below. The experimental procedure was carried out as described in the schematic illustration (Figures S4A and S6D) Specifically, $Ly6E^{hi}$ neutrophils ($1x10^6$ cells per mouse) were intravenously injected into mice bearing 50 mm^3 $4T1_P$ or $4T1_M$ tumors (n=6-7 mice/group), and 4 hours later, mice were treated with anti-PD-1 or IgG control. $Ly6E^{hi}$ neutrophils were adoptively transferred for a total of 3 times. In some experiments, at the time of the last injection, the cells were first labelled with Live Cell Labeling - Red Fluorescence - (Cytopainter, abcam, Cat# ab187965), in accordance with the manufacture's protocol. Tumor volume was measured twice a week. When tumors reached endpoint, the experiment was terminated.

Real-Time quantitative PCR (RT-qPCR)

RNA was extracted from the *in-vitro* $Ly6E^{hi}$ induced cells using Total RNA Purification Kit (Norgen, Ontario, Canada, Cat# 35300). cDNA was synthesized using High-Capacity cDNA Reverse Transcription Kit (Applied Biosystems, California, USA, Cat# 4374966). RT-PCR reaction was performed using SYBR Green Master Mix and ran in the CFX Connect Real-Time PCR Detection System (Bio-Rad, Cat# 4385612). Analysis was performed using the ΔΔCt method. Five biological repeats were carried out. Primers are listed in Table S4.

CD8$^+$ T cell assay

GR1$^+$ and CD8$^+$ T cells were isolated from the spleens of 4T1 tumor bearing mice using EasySep Mouse PE, and MojoSort™ Mouse CD8$^+$ T Cell Isolation Kit (BioLegend, Cat# 480008), respectively. Ly6E^{hi} neutrophils were generated *in-vitro* as described above. The GR1$^+$ cells and Ly6E^{hi} neutrophils (1x10^6/ml) were cultured in serum-free medium for 24 hours to generate conditioned medium (CM). CD8$^+$ T cells (0.5x10^6/ml) were cultured with CM of GR1$^+$ or Ly6E^{hi} neutrophils cells for 24 hours, after which the cells were washed and analyzed by flow cytometry for the evaluation of activated CD8$^+$ T cells (CD8$^+$/CD25$^+$), effector CD8$^+$ T cells (CD8$^+$/CD44$^+$/CD62L$^-$), Granzyme B$^+$ and Ki67$^+$ cells. In some experiments, CD8$^+$ T cells (0.5x10^6/ml) were cultured with conditioned medium of Ly6E^{hi} neutrophils together with neutralizing antibodies anti-IL12p40 (1μg/ml) or anti-IL23p19 (2μg/ml) (R&D systems, Cat# MAB4991 and Cat# AF1619, respectively). After 24 hours, the cells were collected, washed and analyzed by flow cytometry for the evaluation of activated CD8$^+$ T cells (CD8$^+$/CD25$^+$). The experiments were performed using five biological repeats.

Tumor cell killing assay

4T1 cells were seeded in a 24-well plate (20,000 cells/well) along with CD8$^+$ T cells and IFN-induced Ly6E^{hi} neutrophils (200,000 cells, 1:1 ratio) for 24 hours. Subsequently, PI (500 nM) was added to cultures in order to identify dead cells. T cell killing effect was analyzed by flow cytometry. The results are representative of five biological replicates.

dsDNA acquisition

4T1$_P$ and 4T1$_M$ cancer cells were washed and fixed with 4% PFA (Electron Microscopy Sciences, Cat# 15710), 5% sucrose (Merck KGaA, Cat# S0389) in PBS for 15min at RT, following permeabilization and blocking with 0.1% Tween-20 (Santa Cruz Biotechnology, Inc, Cat# sc-29113), 5% BSA (MP BIOMEDICALS, Cat# 02160069) and 25mg/ml glycine (MP BIOMEDICALS, Cat# 808822) for 30min on ice. Anti-dsDNA (0.5μg, Clone 35I9 DNA , Abcam, Cat# ab27156) or IgG2a isotype control (Clone RTK2758, BioLegend, Cat# 400501) were added for 20 min at RT, followed by a secondary antibody (Alexa Fluor 488 donkey anti rat, Invitrogen, Cat# A21208) for another 20min. At least 30,000 events were acquired using a BD CantoII flow cytometer and analyzed by FlowJo software (FlowJo, BD Biosciences). The experiment was performed using 4-5 biological repeats.

STING signaling pathway analysis

Protein estimation from tumor lysates was performed using the Bradford method (Biorad, Cat# 5000006) and isolated proteins were added with a sample buffer, run by SDS-PAGE (10%) (Thermo Fisher Scientific Inc, Cat# BP1311), and then transferred to a polyvinylidene difluoride (PVDF) membrane (Bio-Rad, Cat# 1620177). Subsequently, the membranes were blocked for 1 hour with 5% BSA (MP BIOMEDICALS, Cat# 02160069) in TBST (0.1% Tween 20 in Tris-buffered saline, MP BIOMEDICALS, Cat# 04819620). Membranes were then immunoblotted overnight at 4°C with anti-rabbit STING (Clone D2P2F, Cell signaling, Cat# 13647S), anti-mouse ISG15 (Clone F-9, Santa Cruz Biotechnology, Cat# sc-166755) anti-rabbit IRF3 (Clone D8389, Cell signaling, Cat# 4302S), and anti-rabbit NF-kB (Clone D14E12, Cell signaling, Cat# 8242S). Next, the membranes were blotted with appropriate secondary antibodies (1:10,000 dilution, 1 hour at room temperature, Jackson ImmunoResearch, Cat# 711-035-152) conjugated with horseradish peroxidase. Membranes were developed with WesternBright™ ECL kit (Advansta, Cat# K-12045) and analyzed using an Luminescent Image Analyzer LAS-4000 (Fujifilm Corporation) and "Image Reader LAS-4000" software (Fujifilm Corporation). Densitometric analysis was performed using imageJ analysis software. Band densitometric intensities among different samples were normalized to Actin.

In some experiments, 4T1$_P$ and 4T1$_M$ cells (2x10^6/ml) were incubated with H151 (20μM, Clone 25857, Cayman chemical, Cat# 25857) or IFN-β neutralizing antibody (1 μg/ml, Clone 32401-1, PBL assay science, Cat# 32401-1) or vehicle in DMEM under a humidified 5% CO2 atmosphere at 37°C overnight. Next, culture supernatants were collected and IL-6 levels were determined by standard ELISA (BioLegend, Cat# 431301) or cells were analyzed for PDL1 and MHCI expression (BioLegend, Cat# 124312 and Cat# 116623) using flow cytometry.

To evaluate the induction of Ly6E^{hi} neutrophils from GR1$^+$ cells in the presence of tumor cells, 4T1$_P$ and 4T1$_M$ cells (2x10^6/ml) were cultured in the presence or absence of H151. After 24 hours the cells were washed and cultured in serum-free medium to generate conditioned medium (CM). Subsequently, CM was cultured with GR1$^+$ isolated cells (positive isolation, EasySep Mouse PE, Stemcell Technologies) from the spleens of 4T1 tumor bearing mice under a humidified 5% CO2 atmosphere at 37°C overnight, and analyzed for the expression of Ly6E by flow cytometry. All experiments were performed with at least 3 biological repeats.

Ly6E knock-down in bone marrow cells

Constitutive Cas9-expressing mice (JAX mice, #026179), were used to create CRISPR-Cas9 knockout of immune cells using CHIME (CHimeric IMmune Editing), in line with a previous publication,[80] with some modifications. Specifically, Ly6E gRNA (forward: 5'CACCG AGCAAGCTAAGCCTGCGCAC 3'; reverse: 5'AAAC GTGCGCAGGCTTAGCTTGCT C 3') was cloned into the pXPR_053 vector plasmid containing GFP. Next, lentiviral particles were generated by co-transfecting HEK-293FT cells with packaging (psPAX2, Addgene Plasmid #12259) and envelope (pMD2.G, Addgene Plasmid #12259) plasmids together with pXPR_053 (control vector, Addgene Plasmid # 113591) or pXPR_053 vector containing gRNA specific for the Ly6E gene in StemSpam SFEM II media (Stemcell technologies, Cat# 09605). After 24 hours, fresh SFEM II media was added, and two days later, supernatants were centrifuged at 3000 x RPM for 10 minutes and filtered through a 0.45 μm syringe filter. The viral particles were then transduced into the Lineage negative cells (Lin$^-$) harvested from the bone marrow of donor Cas9 overexpressing mice using MagCellect mouse

hematopoietic cell lineage depletion kit (R&D systems, Cat# MAGM209). Transduced Lin^- cells were then allowed to grow *in-vitro* for 24 hours with effective transduction of over 80%. Subsequently, the cells were intravenously injected (100,000 cells/mouse) into lethally irradiated (1000rad) wild type C57BL/6 mice and allowed to reach bone marrow reconstitution by week 8 post bone marrow transplantation. The recipient mice were implanted with LLC_M cells. When tumor size reached $\sim$50 mm^3, blood was drawn and Ly6E expression was assessed on different immune cells. Subsequently mice were treated with αPD1 or control IgG antibodies (n=6-7 mice/group), and tumor growth was assessed. In some experiments, the recipient mice were used to study Ly6E knockdown *in-vitro* as outlined in the text.

Single cell RNA sequencing on $GR1^+$ cells

The evaluation of $GR1^+$ myeloid cells in responsive and non-responsive tumors was performed by single-cell RNA sequencing (scRNA-seq). Briefly, $4T1_P$ and $4T1_M$ tumors were prepared as single cell suspensions. Subsequently, $GR1^+$ cells were isolated by positive isolation (EasySep Mouse PE, Stemcell Technologies, Cat# 17666). The cells were then washed in PBS with 0.04% BSA and resuspended in 1000 cells/μL PBS. RNA was extracted and immediately was acquired by the 10X Genomics single cell sequencing system, as per manufacture's instructions. Bioinformatic analysis was then carried out as described below.

QUANTIFICATION AND STATISTICAL ANALYSIS

CyTOF pre-processing and analysis

$CD45^+$ gated FCS files were imported into R [v4.1.0] for unsupervised, cluster-based analysis using *CATALYST*[56] [1.24.0]. Aggregated data was transformed (arcsinh) and clustered using *FlowSOM* (10 x 10 grid) and *ConsensusClusterPlus* meta-clustering to yield 25 distinct clusters, annotated based on the expression levels of all markers inspected in parallel. To detect clusters differentially abundant between responders ($4T1_M$) and non-responders ($4T1_P$), generalized linear models (GLMs) were fit using the adapted *edgeR* protocol in *diffcyt*[57] [v1.20.0] – which reports adjusted, FDR-corrected p-values for each comparison.

Single cell RNA-seq alignment and pre-processing

Raw, Illumina base calls (BCLs) were demultiplexed and the resulting FASTQ files were aligned to the mm10 (GRCm38, Ensembl 93) murine reference genome and normalized for sequencing depth using *10x Genomics CellRanger* [v 5.0.1] to generate expression matrices. 82.8-85.7% of reads mapped to the transcriptome across all samples. A median of 3,252 and 2801 unique molecular identifiers (UMI) per cell for $4T1_P$ and $4T1_M$ were observed respectively. R and Python [v3.8.5] were used for all downstream analyses. Genes expressed in <10 cells were discarded. High-quality cells were retained by excluding: (i) cells expressing <500 or >5000 unique genes and (ii) cells with a mitochondrial UMI proportion of >10% - yielding 4711 cells and a total of 14214 detectable genes. *SCTransform* [v0.3.2],[58] accessed via *Seurat* [v4.0.3],[59] was utilized to normalize and scale the data, select 3000 variable features and linearly regress out any remaining influence of mitochondrial UMI% on downstream analyses. *SCTransform* specifically mitigates technical factors, but retains biological heterogeneity, improving downstream analysis.

Classification of cell types

To classify all 4711 cells in an unsupervised manner, *SingleR* [v1.6.1][60] was utilized to compare the transcriptome of each cell to a dual-reference of sorted microarray (ImmGen) and mouse RNA-seq data provided by *celldex* [v1.2.0].[60] Thirty-four cells (1.18%) with ambiguous or poor-quality classifications were discarded – as determined by the SingleR *prunescores* function set to a threshold of 3 absolute mean deviations. Contaminating cells (i.e. non-$GR1^+$, or non-myeloid cells) were discarded and classifications were broadly verified in a supervised manner using known myeloid (*Cd11b*, *Cd11c*), monocytic (*Ly6c*, *Cs1fr*, *MHCII*) and granulocytic (*Ly6g*, *Cs3fr*, *Csf1*) marker genes (1811 monocytic, 2866 granulocytic cells in total).

Dimensionality reduction, unsupervised clustering and differential abundance analysis

Data from all samples was aggregated and, as calculated by the *Seurat* [v4.0.3] functions *RunPCA* and *RunUMAP* respectively (default parameters), the top 3000 variable features and 25 principal components were utilized to generate a uniform manifold approximation and projection (UMAP) for visualization of the data. To assess globular, cellular heterogeneity, transcriptionally distinct cell states were defined by shared k-nearest-neighbour (s-KNN) analysis and Louvain-Jaccard clustering via the *Seurat* [v4.0.3] functions *FindNeighbors* and *FindClusters* respectively, using a resolution of 0.75. Cellular neighborhoods displaying differential abundance between conditions were defined by *DASeq* [v1.0.0][61] using the top 10 principal components and k-values of [50-1000] at 50 step-wise intervals. Non-significant neighborhoods were discarded, as determined by a random permutations test.

Data visualization

Gene expression and UMAPs were visualized using *dynplot* [v1.1.1][62] or as binned, hexplots generated by *schex* [v1.6.3].[63] Where noted, *MAGIC* [v2.0.3][64] was used to impute the data, based on an automatically calculated level of diffusion (parameter *t=auto*). Imputed data was solely used for the purposes of visualization.

Differential gene expression analysis

All differentially expressed genes were identified using the scRNA-seq-specific tool *MAST* [v1.18.0][65] accessed via the *Seurat* [v4.0.3] *FindMarkers* function. Significance was assessed by calculating adjusted FDR p-values using the Bonferroni correction method and a gene was considered to be differentially expressed if its $\log_2$ fold-change was >±0.35.

RNA-velocity and trajectory inference

Using *velocyto* [v0.17],[66] the fractions of unspliced:spliced mRNAs were computed for all ~20,000 genes in the raw FASTQ data. The resulting *LOOM* files were imported to *Seurat* [v4.0.3] and pre-processed as above. RNA-velocity vectors were dynamically modeled using *scvelo* [v0.2.4][67] under default parameters (number of principal components = 30, number of neighbors = 30). To map the differentiation hierarchy of granulocytes, partition-based graph abstraction (*PAGA*, via *scanpy* [v1.8.0][68,69] was combined with velocity-inferred directionality to infer trajectories using the *scvelo* function *scvelo.tl.paga*. Optimal topology was ensured by discarding all non-significant cluster-to-cluster connections (connectivity score <0.1) and the resulting trajectories were projected back onto the original UMAP using *dynplot* [v1.1.1].

Gene modules and pathway analysis

To identify genes with pseudotime-associated patterns of expression, negative binomial generalized additive models (NB-GAMs) were fit to ~14,000 genes and the significance of association was statistically tested by *tradeSeq* [v1.6.0].[70] NB-GAMs were fit using the parameter *nknots=6* – a conservative estimate, as determined by the *tradeSeq* function, *evaluateK*, to avoid overfitting. Expression patterns were binned (n=20) along pseudotime and clustered via *clusterExperiment* [v2.12.0][71] to define distinct gene modules. To characterize each module, over-representation tests were performed using *clusterProfiler* [v4.0.0][72] and gene-lists from the HALLMARK database[75] (biological processes) and *msigdbr* [v7.4.1] (category = C3, transcription factors). The latter determines which, if any, transcription factors (TFs) regulate the genes present in each module. Only significantly enriched (FDR < 0.01, Bonferroni correction method) processes and TFs were retained.

Trajectory alignment

To compare trajectory lineages, a common pseudotemporal axis was defined using *cellAlign* [v0.1.0][21] - set to default, globalAlignment parameters as specified here: https://github.com/shenorrLab/cellAlign. In brief, inferred pseudotime values (defined by PAGA/RNA velocity), and the normalized expression values of all genes in modules common to both lineages were utilized to align the trajectories across 200 interpolated points and module enrichment values were averaged at corresponding, aligned pseudotime values.

Human analysis of blood scRNA-seq

Raw, scRNA-seq expression matrices were downloaded from the GEO Omnibus database (GSE127465)[35] (n=8, blood, patients with NSCLC at baseline). Data was imported into *Seurat* [v4.0.3] and pre-processed using *SCTransform* [v0.3.2] with identical filtering criteria to mouse - yielding 13403 cells and a total of 22413 detectable genes. To classify all 13404 cells in an unsupervised manner, *SingleR* [v1.6.1] was utilized to compare the transcriptome of each cell to the Human Primary Cell Atlas reference, as provided by *celldex* [v1.2.0]. 1701 (14.4%) non-immune cells or cells with ambiguous or poor-quality classifications were excluded. Human-specific gene-lists from the HALLMARK database, as accessed in R via *msigdbr* [v7.4.1], for (i) *interferon_alpha_response* (ii) *interferon_gamma_response* and (iii) *tnfa_signalling_via_nfkb* were combined to generate a functional signature representative of Ly6E^{hi} neutrophils. The enrichment of each, individual cell for the resulting signature was scored using the Seurat [v4.0.3] ssGSEA-like function, *AddModuleScore*.

Cell-specific deconvolution

1440 bulk RNA-seq samples were obtained from[37–40] and deconvolved using *CIBERSORTx*[73] to obtain relative cell-type frequency estimates for 10 distinct immune cell subsets (LM10 signature matrix). Representative cell-type-specific expression profiles and per-gene variance estimates were subsequently imputed using *CIBERSORTx* [mode: Group] at an aggregated per-group level (non-responders (NR) vs. responders (R)). To obtain robust statistical measures, data was subsequently bootstrapped using a negative binominal (NB) model-based simulation - where per-gene NB dispersion estimates were calculated as (CV_{GeneA}^2)-1/μ(GeneA). Each simulated sample was analyzed by gene-set variation analysis (*GSVA*) [v1.47.3][74] to score the enrichment of a 15-gene Ly6E^{hi}, IFN-γ/α stimulated signature (Neut$_{IFN}$-15: IFIT1, MX1, HERC5, IFI6, ISG15, IFIT3, RSAD2, GBP1, IFIT2, XAF1, PARP9, UBE2L6, IRF7, PARP14, APOL6 – derived from equivalent human Ly6E^{hi} neutrophils), within neutrophils specifically, or the pre-existing IFNγ-6 signature (Ayers et al., 2017) (IFNγ-6: IDO1, CXCL10, CXCL9, HLA-DRA, STAT1 and IFNG) at the level of the convolved data (as intended by the original authors). Neut$_{IFN}$-15 represents the 15 genes with near-exclusive expression in IFN-stimulated, Ly6E^{hi} neutrophils relative to all other human neutrophils.

Statistical analysis

All statistical tests were performed in R [v4.1.0]. Statistical, pairwise comparisons for ELISA, LEGENDplex and Flow Cytometry data were performed using unpaired, two-sample Mann-Whitney tests (R function: wilcox.test) for n=2, or by one-way ANOVA coupled with Tukey's post-hoc HSD test for n>2 (*R functions*: *aov and TukeyHSD*). Two-sample Kolmogorov-smirnov tests (R function: ks.test) were utilized to compare tumor growth curves. Mice were randomized before tumor implantation. The analysis of the results

CellPress
OPEN ACCESS

was performed blindly. At least 5 mice per group were used in order to reach statistical power considering a Gaussian distribution. For *in-vitro* studies, at least three biological repeats were carried out, unless indicated otherwise in the text. Where appropriate (e.g. differential gene expression analysis), p-values were adjusted using the Bonferroni correction method to control for type I error rates i.e. false discovery rate (FDR). In all cases, significant differences were considered if p-values or FDR were <0.01. The number of samples or independent experiments are indicated in the text. For patients with NSCLC and melanoma, the investigators were blinded to allocation (i.e. RECIST categories) during experiments and outcome assessment. Co-variates including age, sex and stage were not controlled for.

Cancer Cell, Volume *42*

Supplemental information

Interferon-stimulated neutrophils

as a predictor of immunotherapy response

Madeleine Benguigui, Tim J. Cooper, Prajakta Kalkar, Sagie Schif-Zuck, Ruth Halaban, Antonella Bacchiocchi, Iris Kamer, Abhilash Deo, Bar Manobla, Rotem Menachem, Jozafina Haj-Shomaly, Avital Vorontsova, Ziv Raviv, Chen Buxbaum, Petros Christopoulos, Jair Bar, Michal Lotem, Mario Sznol, Amiram Ariel, Shai S. Shen-Orr, and Yuval Shaked

SUPPLEMENTARY DATA

Supplementary Tables

Table S3. List of tumor models used, related to Figure 1.

Cell Line	Clones	Cancer Type	Mouse Strain	Model Category
4T1	$4T1_P$, $4T1_M$	Breast Carcinoma	BALB/c	Mutagenesis
RENCA	$RENCA_P$, $RENCA_M$	Renal Adenocarcinoma	BALB/c	Mutagenesis
LLC	LLC_P, LLC_M	Lung Carcinoma	C57BL/6	Mutagenesis
EMT6	$EMT6_P$	Breast Carcinoma	BALB/c	Spontaneous
LLC	LLC_P	Lung Carcinoma	C57BL/6 x CBA	Backcross

The initial three rows (mutagenesis) are tumor-dependent models. The remaining rows are host-dependent models. *P = parental cells, M = mutagenized cells.*

Table S4. List of primers used for RT-qPCR, related to Figure 4.

Gene	Forward	Reverse
mTnfα	CTGAACTTCGGGGTGATCGG	GGCTTGTCACTCGAATTTTGAGA
mCxcl1	CTGGGATTCACCTCAAGAACATC	CAGGGTCAAGGCAAGCCTC
mIL1β	TCTCAGATTCACAACTGTTCGTG	AGAAAATGAGGTCGGTCTCACTA
mIL23a	CAGCAGCTCTCTCGGAATCTC	TGGATACGGGGCACATTATTTTT
mSaa3	TGCCATCATTCTTTGCATCTTGA	CCGTGAACTTCTGAACAGCCT
mCcl3	TGTACCATGACACTCTGCAAC	CAACGATGAATTGGCGTGGAA
mCcl6	AAGAAGATCGTCGCTATAACCCT	GCTTAGGCACCTCTGAACTCTC

Supplementary Figures
Figure S1.

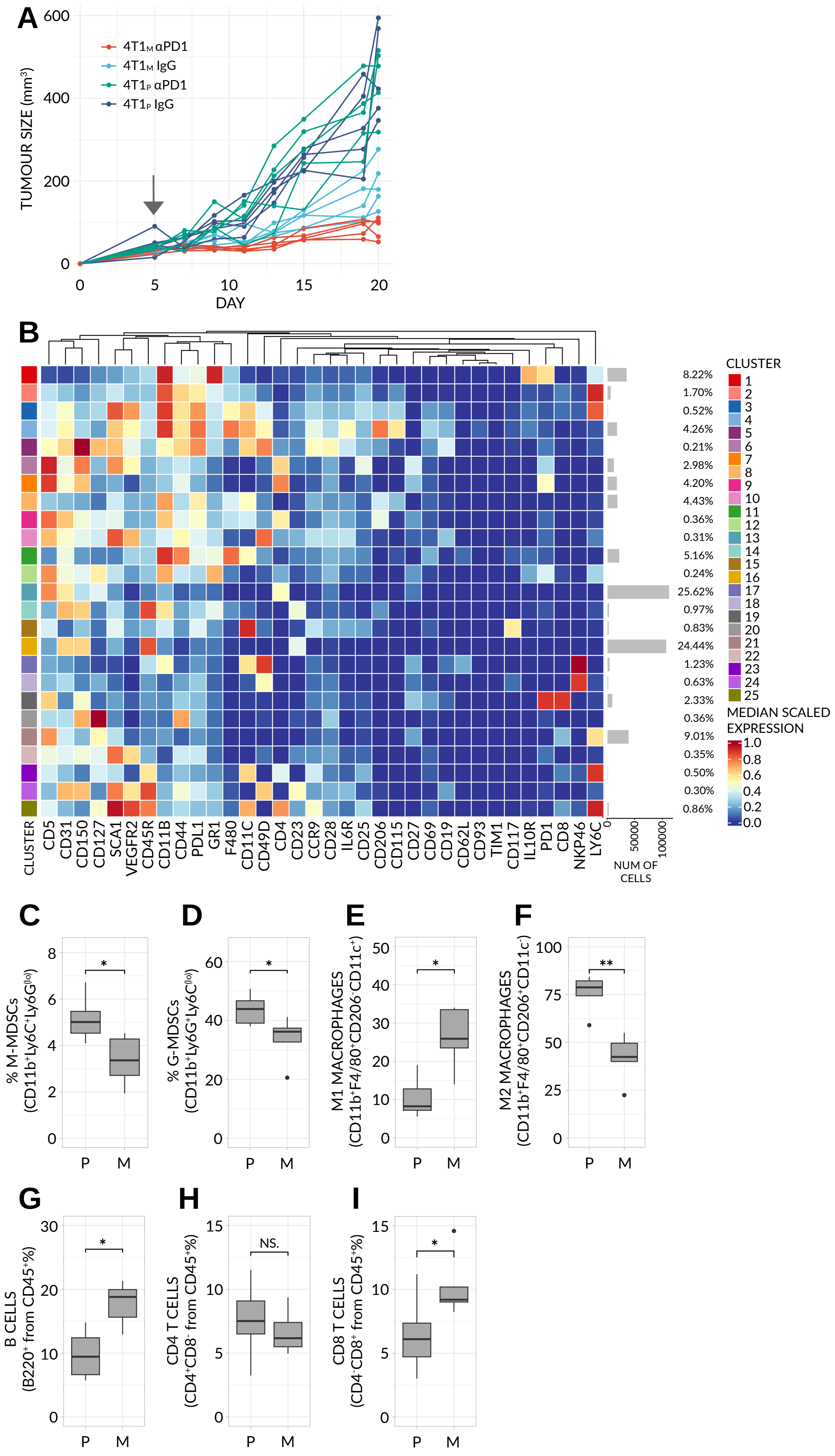

Figure S1. Mutagenized 4T1 tumors support an immunogenic TME, related to Figure 2. **(A)** Raw, individual tumor growth profiles of BALB/c mice implanted with parental $4T1_P$ (P) or mutagenized $4T1_M$ (M) breast cancer and treated with αPD1 or control IgG antibodies (n=5 mice/group). Treatment was initiated at a tumor size of ~50mm^3 (arrow). **(B)** CD45$^+$ cells from the tumor microenvironment (TME) of $4T1_P$ (205,678 cells) and $4T1_M$ (236,251 cells) tumors were segregated into 25 distinct, unsupervised clusters (n=5 mice pooled/group). A heatmap of the scaled, median expression level of each CYTOF marker is shown and was used to annotate each cluster. The frequency of each cluster, calculated as a percentage of total CD45$^+$ cells, is shown as a bar-plot (right). ***(C-I)*** Frequencies of: monocytic MDSCs (M-MDSCs) **(C)**; granulocytic MDSCs (G-MDSCs) **(D)**; M1 macrophages **(E)**; M2 macrophages **(F)**; B cells **(G)**; CD4$^+$ T cells **(H)**; CD8$^+$ T cells **(I)** in the TME of untreated 4T1 tumors, as determined by flow cytometry (n=5 mice/group). *In (C-I), significance was assessed by means of a one-way Mann-Whitney test (NS, p>0.01; *, p<0.01; **, p<0.001).*

Figure S2.

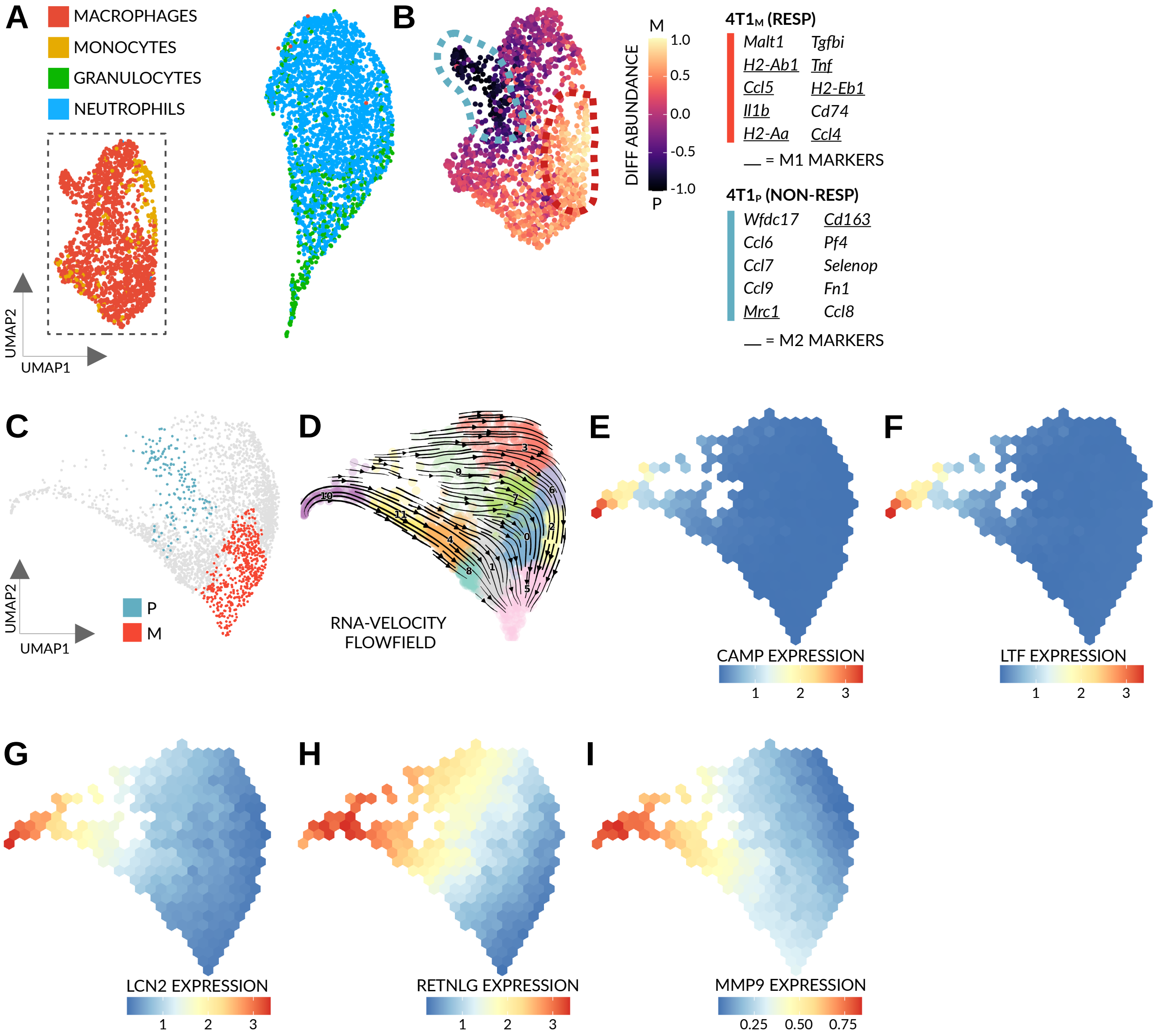
A
MACROPHAGES
MONOCYTES
GRANULOCYTES
NEUTROPHILS
UMAP2
UMAP1
B
M
1.0
0.5
0.0
-0.5
-1.0
P
DIFF ABUNDANCE
4T1M (RESP)
Malt1 Tgfbi
H2-Ab1 Tnf
Ccl5 H2-Eb1
Il1b Cd74
H2-Aa Ccl4
= M1 MARKERS
4T1P (NON-RESP)
Wfdc17 Cd163
Ccl6 Pf4
Ccl7 Selenop
Ccl9 Fn1
Mrc1 Ccl8
= M2 MARKERS
C
UMAP2
UMAP1
P
M
D
RNA-VELOCITY
FLOWFIELD
E
CAMP EXPRESSION
1 2 3
F
LTF EXPRESSION
1 2 3
G
LCN2 EXPRESSION
1 2 3
H
RETNLG EXPRESSION
1 2 3
I
MMP9 EXPRESSION
0.25 0.50 0.75

Figure S2. scRNA-seq of GR1⁺ cells from 4T1 tumors, related to Figure 3. 10X scRNA-seq was performed on $GR1^+$ cells obtained from 4T1 breast cancer parental ($4T1_P$) (non-responsive) and mutagenized ($4T1_M$) (responsive) tumors (n=3 mice pooled/group). Data is identical to that used in Fig 3. **(A)** UMAP plot of 4711 filtered, $GR1^+$ cells from which 1825 cells are monocytes ($4T1_P$ = 815 cells, $4T1_M$ = 1010 cells). Cells are colored by cell type. **(B)** Differential abundance plot of monocytic cells (dotted box in (A)), highlighting two significantly enriched, cellular neighborhoods (dotted lines). The top 10, most significant marker genes of each neighborhood are listed (FDR<0.001, $\log_2$ fold-change >1.5). Genes underlined are classical markers of M1 (responders) and M2 (non-responders) macrophages. **(C-I)** scRNA-seq analysis of the neutrophil subpopulation displaying **(C)** a UMAP plot of 2886 $GR1^+$ neutrophils, highlighting two significant, differentially abundant cellular neighborhoods found in $4T1_P$ and $4T1_M$ tumors. Data is identical to that shown in Fig 3. *Significance of each neighborhood was determined via a randomized, permutations test;* **(D)** Raw, RNA-velocity flow-field vectors projected back onto the UMAP; **(E-I)** Binned, normalized expression of known granulocytic progenitor genes. *Data was imputed for visual clarity.*

FIGURE S3

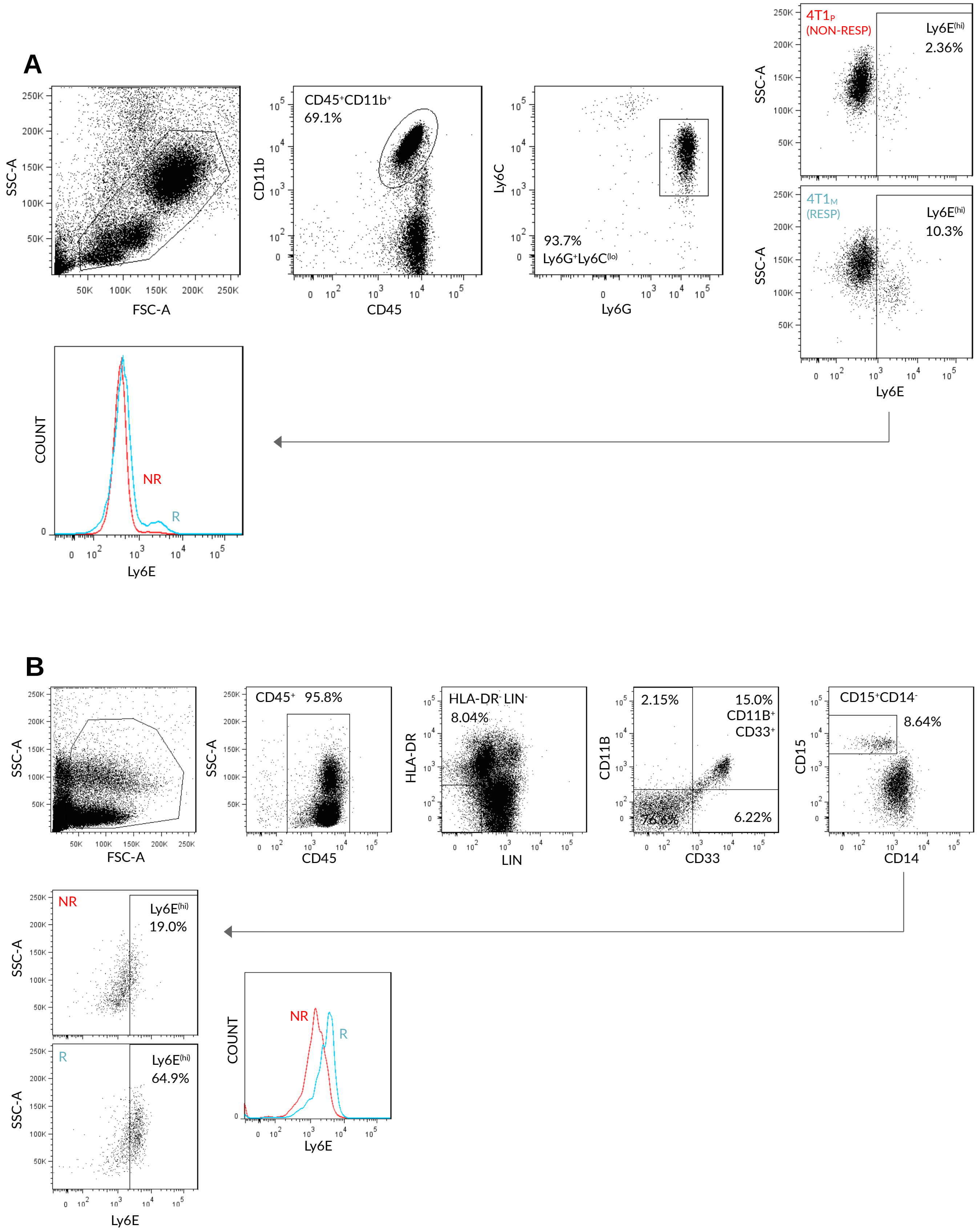
A
SSC-A
FSC-A
CD45+CD11b+
69.1%
CD11b
CD45
Ly6C
Ly6G
93.7%
Ly6G+Ly6C(lo)
4T1P
(NON-RESP)
Ly6E(hi)
2.36%
4T1M
(RESP)
Ly6E(hi)
10.3%
Ly6E
COUNT
NR
R
B
CD45+ 95.8%
CD45
HLA-DR- LIN-
8.04%
HLA-DR
LIN
2.15%
15.0%
CD11B+
CD33+
76.6%
6.22%
CD11B
CD33
CD15+CD14-
8.64%
CD15
CD14
NR
Ly6E(hi)
19.0%
R
Ly6E(hi)
64.9%

Figure S3. Gating strategy of Ly6E$^{(hi)}$ neutrophils in mouse and human, related to Figures 3 and 6. **(A)** Representative flow cytometry plots taken from the blood of BALB/c mice bearing parental 4T1$_P$ (NR) or mutagenized 4T1$_M$ (R) breast carcinoma. The analysis was performed on whole blood cells gated for CD45$^+$/CD11b$^+$, Ly6C$^{(lo)}$/Ly6G$^+$, and Ly6E$^{(hi)}$. Notably, the percentage of Ly6E$^{(hi)}$ gated cells is higher in mice bearing 4T1$_M$ tumors. **(B)** Representative flow cytometry plots taken from peripheral blood mononuclear cells (PBMCs) of patients with NSCLC at baseline. Data is stratified into non-responders (NR) and responders (R) based on RECIST criteria at 3 and/or 6 months. The analysis was performed on PBMCs gated for CD45$^+$, Lin$^-$/HLA-DR$^-$, CD14$^-$/CD15$^+$, and Ly6E$^{(hi)}$. Notably, the percentage of Ly6E$^{(hi)}$ gated cells is higher in responding patients.

FIGURE S4

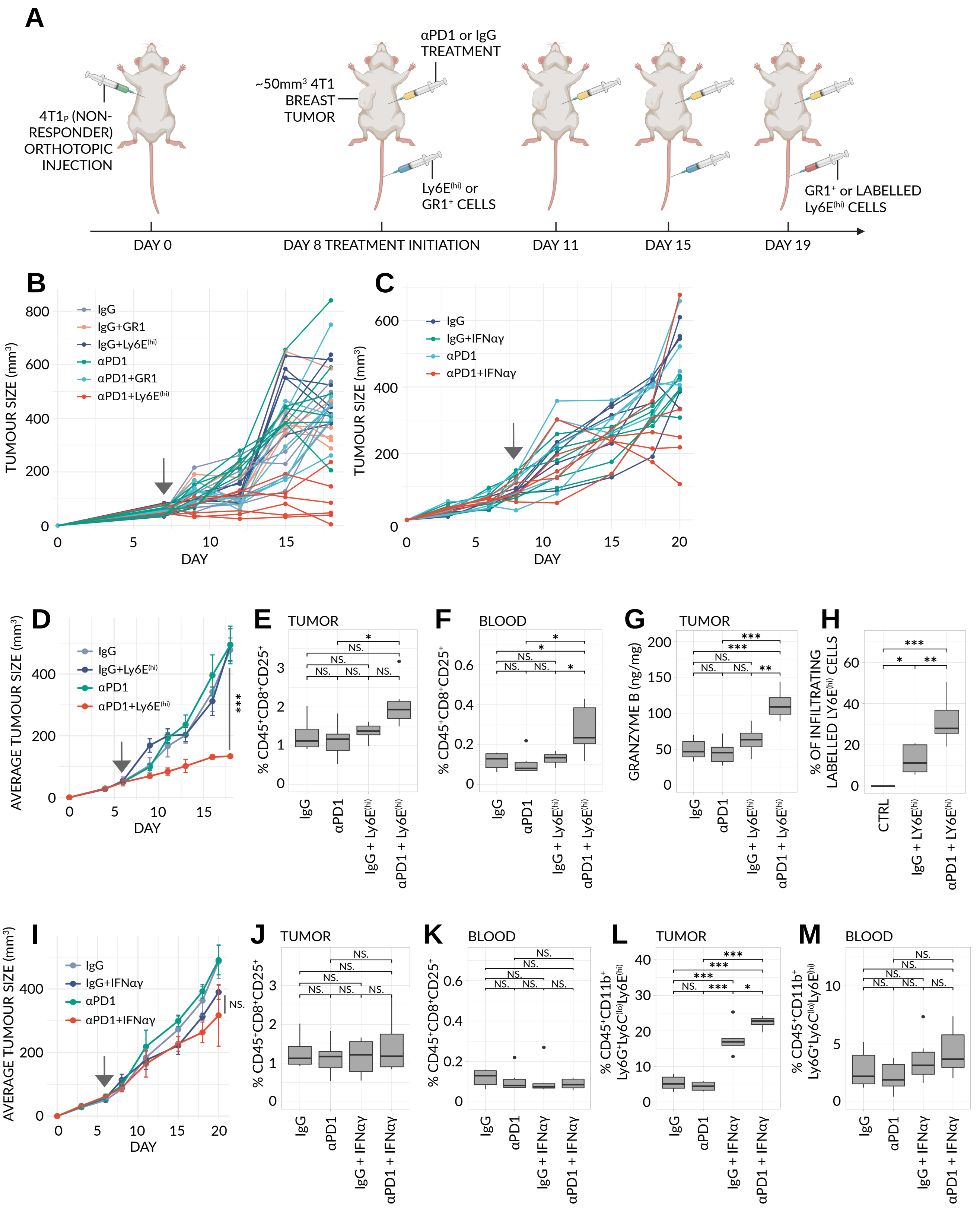
A
4T1P (NON-RESPONDER) ORTHOTOPIC INJECTION
~50mm3 4T1 BREAST TUMOR
αPD1 or IgG TREATMENT
Ly6E(hi) or GR1+ CELLS
GR1+ or LABELLED Ly6E(hi) CELLS
DAY 0
DAY 8 TREATMENT INITIATION
DAY 11
DAY 15
DAY 19
B
TUMOUR SIZE (mm3)
DAY
IgG
IgG+GR1
IgG+Ly6E(hi)
αPD1
αPD1+GR1
αPD1+Ly6E(hi)
C
IgG
IgG+IFNαγ
αPD1
αPD1+IFNαγ
D
AVERAGE TUMOUR SIZE (mm3)
E
TUMOR
% CD45+CD8+CD25+
F
BLOOD
G
TUMOR
GRANZYME B (ng/mg)
H
% OF INFILTRATING LABELLED LY6E(hi) CELLS
CTRL
IgG + LY6E(hi)
αPD1 + LY6E(hi)
IgG
αPD1
IgG + Ly6E(hi)
αPD1 + Ly6E(hi)
I
J
TUMOR
K
BLOOD
L
TUMOR
% CD45+CD11b+ Ly6G+Ly6C(lo)Ly6E(hi)
M
BLOOD
IgG + IFNαγ
αPD1 + IFNαγ
NS.

Figure S4. Ly6E$^{(hi)}$ neutrophil cell therapy, but not systemic IFNα/γ treatment, induces immunotherapy response, related to Figure 4. **(A)** Schematic overview of the adoptive transfer protocol (see Methods for details). In brief, non-responding 4T1 tumors ($4T1_P$) were orthotopically injected into BALB/c mice. Treatment with either a monotherapy (control IgG or αPD1) or a combined therapy, with $GR1^+$ or Ly6E$^{(hi)}$ neutrophils, was initiated on Day 8 at an average tumor size of ~50mm^3. Additional doses were given throughout the time-course as marked. On Day 19, Ly6E$^{(hi)}$ neutrophils were labeled with a Live Cell Labeling allophycocyanin (APC) kit to track migration of these cells into the TME (see: Fig S4H) and as written in Methods. **(B-C)** Raw, individual tumor growth profiles of mice bearing parental, non-responsive $4T1_P$ breast tumors treated with either a monotherapy (control IgG or αPD1) or a combined therapy, with $GR1^+$ or Ly6E$^{(hi)}$ neutrophils (n=5 mice/group) **(B)**; or systemic IFNα/γ (n=6 mice/group) **(C)**; as specified. Treatment was initiated at a tumor size of ~50mm^3 (arrow) in all cases. **(D-H)** Ly6E$^{(hi)}$ neutrophils, generated *in-vitro,* were adoptively transferred into mice bearing parental, non-responsive $4T1_P$ breast tumors, with or without αPD1 or IgG control therapy. Data is a biological repeat of the experiment shown in Fig 4. **(D)** Averaged tumor growth profiles for all conditions (n=5 mice/group). Frequencies of activated, $CD25^+CD8^+$ T cells in the TME **(E)**; and in the blood **(F)** at end point; as determined by flow cytometry. Granzyme B concentrations in tumor lysates, as measured by ELISA **(G)** and frequency of Ly6E$^{(hi)}$ neutrophils that have infiltrated into the tumor, as tracked by APC cell labeling (see: Fig. S4A) **(H)**. **(I-M)** In a separate experiment, mice bearing $4T1_P$ tumors were systemically administered IFNα/γ with or without αPD1 or IgG control therapy (n=6 mice/group). **(I)** Averaged tumor growth profiles for all conditions (see: Fig. S4C for raw data). Frequencies of: activated, $CD25^+CD8^+$ T cells and Ly6E$^{(hi)}$ neutrophils in the TME at end-point **(J, L)** and in the blood **(K, M)** were analyzed by flow cytometry. *Significance was assessed by means of a two-sample KS-test (growth) and a one-way ANOVA and Tukey's post-hoc HSD test (flow) (NS, p>0.01; * p<0.01; **, p<0.001; ***, p<0.0001).*

FIGURE S5

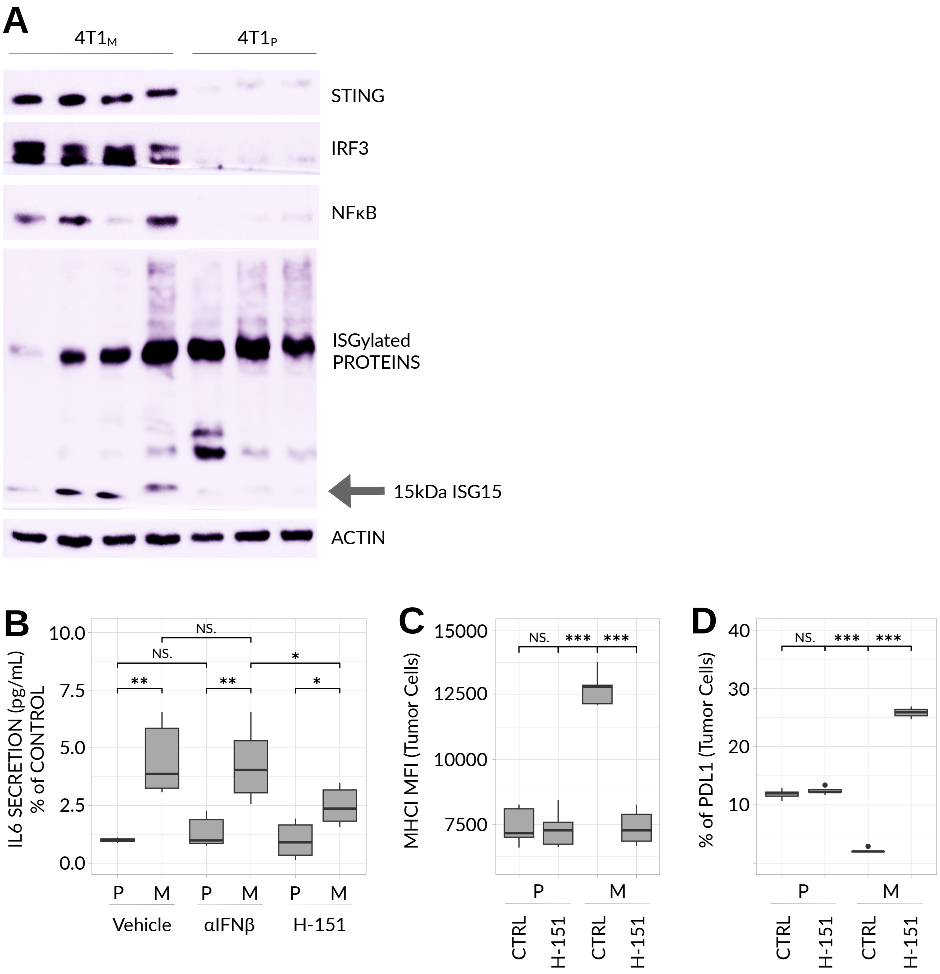
A
4T1M
4T1P
STING
IRF3
NFκB
ISGylated PROTEINS
15kDa ISG15
ACTIN
B
IL6 SECRETION (pg/mL) % of CONTROL
10.0
7.5
5.0
2.5
0.0
NS.
**
*
P M P M P M
Vehicle αIFNβ H-151
C
MHCI MFI (Tumor Cells)
15000
12500
10000
7500
***
P M
CTRL H-151 CTRL H-151
D
% of PDL1 (Tumor Cells)
40
30
20
10

Figure S5. STING activity is a hallmark of 4T1$_M$ tumors, related to Figure 5. **(A)** Western blot of STING-pathway related proteins in 4T1$_P$ and 4T1$_M$ tumor lysates (n=3-4 biological repeats/group). **(B)** Levels of secreted IL6, as assayed by ELISA from the conditioned media of cultured 4T1$_P$ (P) and 4T1$_M$ (M) cell lines, alone or in the presence of αIFNβ or the STING-inhibitor H151 (n=6 biological repeats/group). **(C-D)** Levels of cell-surface MHCI **(C)**; or PDL1 **(D)** on cultured 4T1$_P$ (P) and 4T1$_M$ (M) cell lines as determined by flow cytometry with or without the STING-inhibitor H151 (n=3-5 biological repeats/group). *In (B-D), significance was assessed by means of a one-way ANOVA and Tukey's post-hoc HSD test (NS, p>0.01; *, p<0.01; **, p<0.001; ***, p<0.0001).*

FIGURE S6

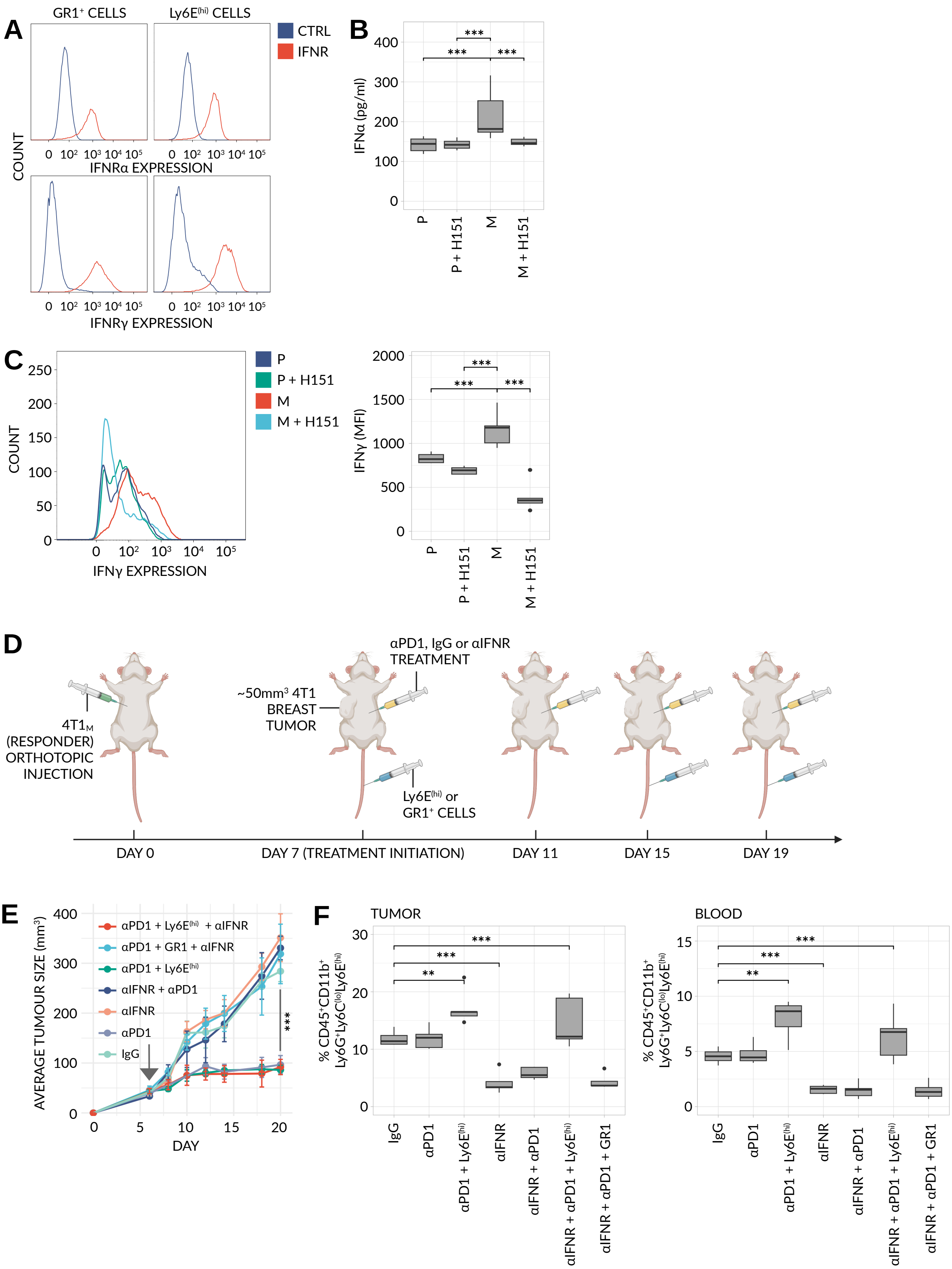
A
GR1+ CELLS
Ly6E(hi) CELLS
CTRL
IFNR
COUNT
IFNRα EXPRESSION
IFNRγ EXPRESSION
B
IFNα (pg/ml)
P
P + H151
M
M + H151
C
COUNT
IFNγ EXPRESSION
IFNγ (MFI)
D
4T1M (RESPONDER) ORTHOTOPIC INJECTION
~50mm³ 4T1 BREAST TUMOR
αPD1, IgG or αIFNR TREATMENT
Ly6E(hi) or GR1+ CELLS
DAY 0
DAY 7 (TREATMENT INITIATION)
DAY 11
DAY 15
DAY 19
E
AVERAGE TUMOUR SIZE (mm³)
DAY
αPD1 + Ly6E(hi) + αIFNR
αPD1 + GR1 + αIFNR
αPD1 + Ly6E(hi)
αIFNR + αPD1
αIFNR
αPD1
IgG
F
TUMOR
BLOOD
% CD45+CD11b+ Ly6G+Ly6C(lo)Ly6E(hi)
IgG
αPD1
αPD1 + Ly6E(hi)
αIFNR
αIFNR + αPD1
αIFNR + αPD1 + Ly6E(hi)
αIFNR + αPD1 + GR1

Figure S6. Ly6E$^{(hi)}$ neutrophils rescue anti-PD1 responsiveness in 4T1 tumors made resistant through IFNR blockade, related to Figure 5. **(A)** Expression of IFNα/γ and their cognate receptors (IFNR-α/γ) within tumor and immune cells was examined using spleens from BALB/c mice, bearing $4T1_P$ tumors, that were harvested and prepared as single-cell suspensions (n=5 biological repeats/group). Expression levels of INFRα and IFNRγ were subsequently analyzed on the surface of $GR1^+$ and Ly6E$^{(hi)}$ neutrophils using flow cytometry. Histogram plots, depicting expression levels, are shown. **(B-C)** Non-responsive $4T1_P$ (P) and responsive $4T1_M$ (M) cancer cells were cultured in the presence or absence of the STING inhibitor, H151 (see: Methods) (n=8 biological repeats/group). The cells were prepared as lysates, and the levels of IFNα were quantified using ELISA **(B)**, while IFNγ levels were assessed by means of flow cytometry (n=5 biological repeats/group) **(C)**. Of note, IFNγ levels remained below the detection level of ELISA – necessitating the use of flow cytometry. A representative histogram and summarizing boxplots displaying the mean fluorescent intensity (MFI) of IFNγ are shown. **(D)** Schematic overview of the rescue experiment, whereby Ly6E$^{(hi)}$ neutrophils are adoptively transferred into mice bearing $4T1_M$ tumors made resistant to anti-PD1. In brief, $4T1_M$ tumors were orthotopically injected into BALB/c mice and treatment with monotherapy (control IgG, αPD1, αIFNRα/γ) or combined therapy (αPD1, αIFNRα/γ) with or without adoptively transferred $GR1^+$ or Ly6E$^{(hi)}$ neutrophils, was initiated on Day 7 at an average tumor size of ~$50mm^3$. Additional doses were given throughout the time-course as marked. On day 20, mice were sacrificed, and tumors and blood were analyzed for Ly6E$^{(hi)}$ neutrophils. **(E)** Averaged tumor growth profiles for the mice described above are shown (n=5 mice/group). **(F)** Tumors were harvested and prepared as single-cell suspensions and blood was drawn for the analysis of Ly6E$^{(hi)}$ neutrophil levels using flow cytometry. *Significance was assessed by means of a two-sample KS-test (growth) and one-way ANOVA and Tukey's post-hoc HSD test (flow and ELISA) (**, p < 0.001; ***, p<0.0001).*

FIGURE S7

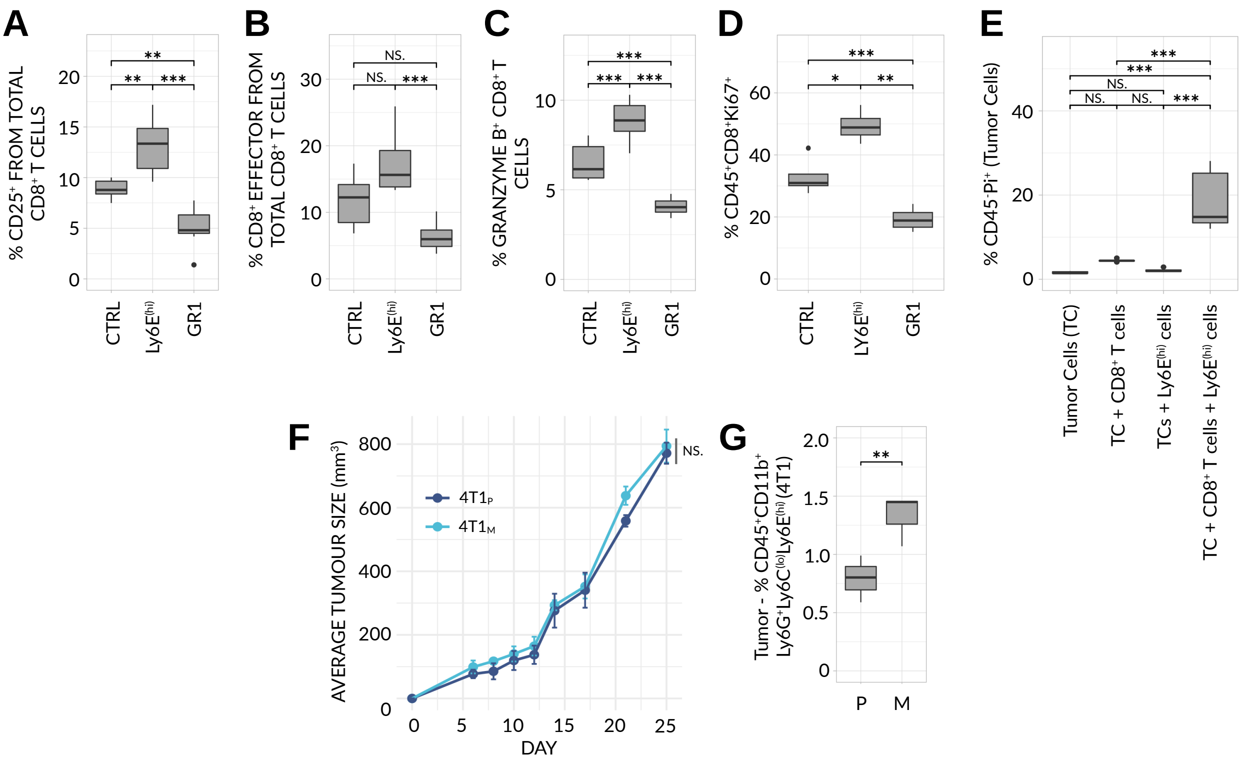
A
% CD25+ FROM TOTAL CD8+ T CELLS
CTRL
Ly6E(hi)
GR1
B
% CD8+ EFFECTOR FROM TOTAL CD8+ T CELLS
C
% GRANZYME B+ CD8+ T CELLS
D
% CD45+CD8+Ki67+
CTRL
LY6E(hi)
GR1
E
% CD45-PI+ (Tumor Cells)
Tumor Cells (TC)
TC + CD8+ T cells
TCs + Ly6E(hi) cells
TC + CD8+ T cells + Ly6E(hi) cells
F
AVERAGE TUMOUR SIZE (mm3)
DAY
4T1P
4T1M
NS.
G
Tumor - % CD45+CD11b+ Ly6G+Ly6C(lo)Ly6E(hi) (4T1)
P
M

Figure S7. Ly6E$^{(hi)}$ neutrophils contribute to and act upstream of anti-tumor T cell activity, related to Figure 5. **(A-D)** $CD8^+$ T cells, obtained from the spleens of $4T1_P$ tumor bearing mice, were cultured *in-vitro* either alone (CTRL) or with conditioned media derived from $GR1^+$ cells or IFNαγ-induced, Ly6E$^{(hi)}$ (Ly6E) neutrophils (n=8 biological repeats/group). Frequencies or levels of: activated, $CD8^+CD25^+$ T cells **(A)**; effector $CD8^+$ T cells **(B)**; Granzyme B^+ $CD8^+$ T cells **(C)**; and proliferating $CD8^+$ T cells **(D)**, were determined by flow cytometry. **(E)** In a separate experiment, $4T1_P$ cells were cultured in the presence of $CD8^+$ T cells or Ly6E$^{(hi)}$ neutrophils for 24 hours in a ratio of 1:10:10. T cell killing efficacy was analyzed by flow cytometry using PI to detect dead tumor cells (n=5 biological repeats/group). **(F-G)** $4T1_P$ and $4T1_M$ cells were implanted in 8 week old SCID mice (n=3 mice/group). **(F)** Averaged tumor growth curves for both tumor types. **(G)** Frequencies of Ly6E$^{(hi)}$ neutrophils as assessed by flow cytometry in blood profiles as tumors reached a size of ~$50mm^3$. *Significance was assessed by means of a two-sample KS-test (growth) and a one-way ANOVA and Tukey's post-hoc HSD test (flow) (NS, p>0.01; * p<0.01; **, p<0.001; ***, p<0.0001).*

FIGURE S8

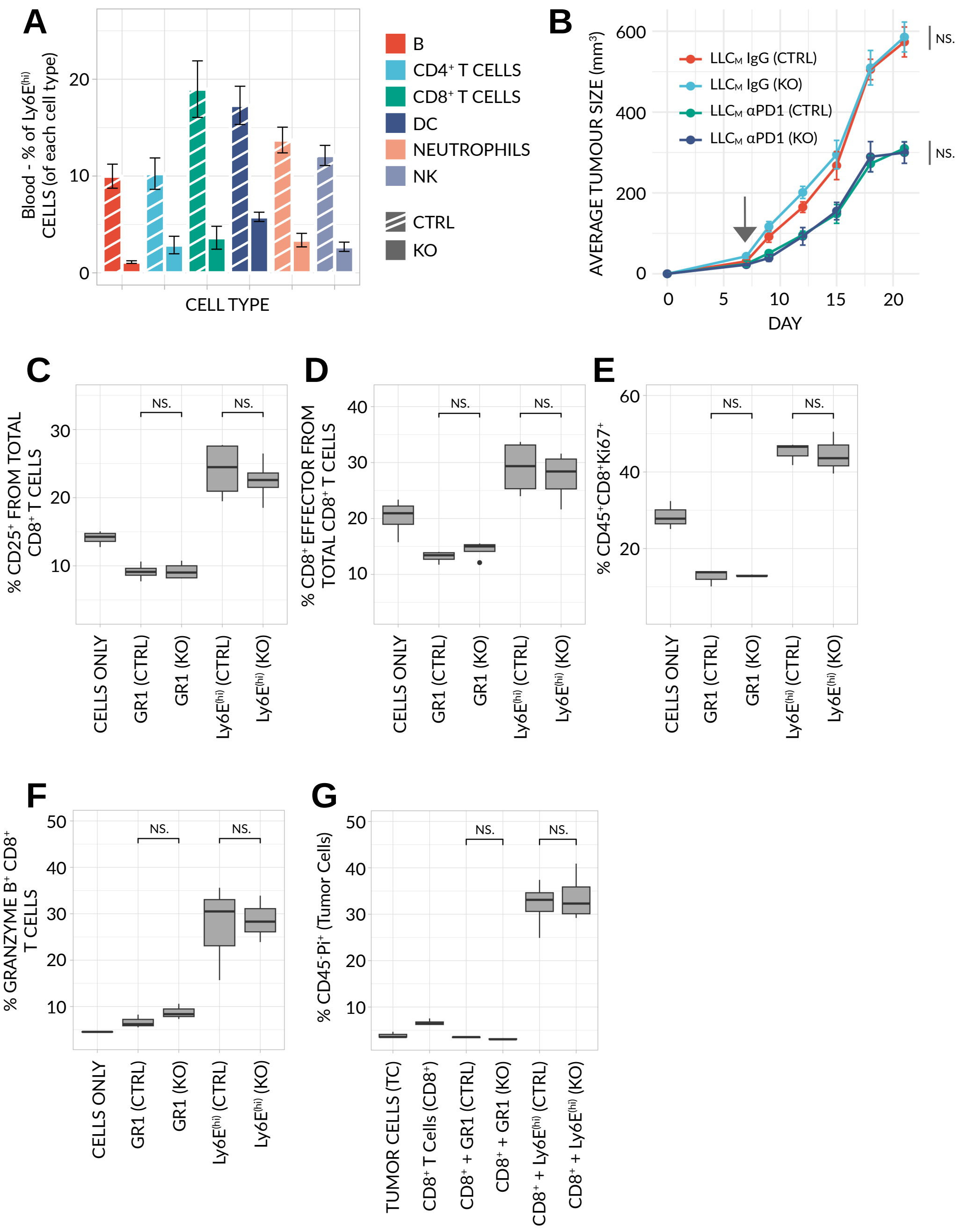

A
Blood - % of Ly6E(hi) CELLS (of each cell type)
CELL TYPE
B
CD4+ T CELLS
CD8+ T CELLS
DC
NEUTROPHILS
NK
CTRL
KO
B
AVERAGE TUMOUR SIZE (mm3)
DAY
LLCM IgG (CTRL)
LLCM IgG (KO)
LLCM αPD1 (CTRL)
LLCM αPD1 (KO)
NS.
C
% CD25+ FROM TOTAL CD8+ T CELLS
D
% CD8+ EFFECTOR FROM TOTAL CD8+ T CELLS
E
% CD45+CD8+Ki67+
CELLS ONLY
GR1 (CTRL)
GR1 (KO)
Ly6E(hi) (CTRL)
Ly6E(hi) (KO)
F
% GRANZYME B+ CD8+ T CELLS
G
% CD45-PI+ (Tumor Cells)
TUMOR CELLS (TC)
CD8+ T Cells (CD8+)
CD8+ + GR1 (CTRL)
CD8+ + GR1 (KO)
CD8+ + Ly6E(hi) (CTRL)
CD8+ + Ly6E(hi) (KO)

Figure S8. Ly6E itself has no functional role in immunotherapy response, related to Figure 5. **(A-G)** Lin^- bone-marrow progenitor cells, isolated from C57BL/6 strains over-expressing Cas9, were transfected with lentivirus carrying a single guide RNA GFP-CRISPR-Cas9 construct targeting the Ly6E locus. A transfection rate of ~80%, as measured by GFP expression, was observed. Transfected cells were subsequently implanted into irradiated, otherwise WT C57BL/6 mice (C57BL/6Ly6EKO). **(A)** Frequencies of $Ly6E^{(hi)}$ cells across all major immune subtypes were determined by flow cytometry (n=5 mice/group). CTRL = single guide RNA without the Ly6E-targeting. **(B)** Averaged tumor growth profiles for C57BL/6CTRL or C57BL/6Ly6EKO mice implanted with mutagenized Lewis-lung carcinoma (LLC_M), and treated with αPD1 or control IgG antibodies (n=6-7 mice/group). **(C-G)** $CD8^+$ T cells, obtained from the spleens of LLC tumor bearing mice, were cultured *in-vitro* either alone or with conditioned media derived from $GR1^+$ cells or IFNαγ-induced, $Ly6E^{(hi)}$ neutrophils (n=4-5 biological repeats/group). Frequencies or levels of: activated, $CD8^+CD25^+$ T cells **(C)**; effector $CD8^+$ T cells **(D)**; proliferating $CD8^+$ T cells **(E)**; and Granzyme B^+ $CD8^+$ T cells **(F)**, were determined by flow cytometry. **(G)** In a separate experiment, $4T1_P$ cells were cultured in the presence of $CD8^+$ T cells or $Ly6E^{(hi)}$ neutrophils for 24 hours in a ratio of 1:10:10. T cell killing efficacy was analyzed by flow cytometry using PI to detect dead tumor cells (n=4 biological repeats/group). *Significance was assessed by means of a two-sample KS-test (growth) and one-way ANOVA and Tukey's post-hoc HSD test (flow and ELISA) (NS, p>0.01).*

FIGURE S9

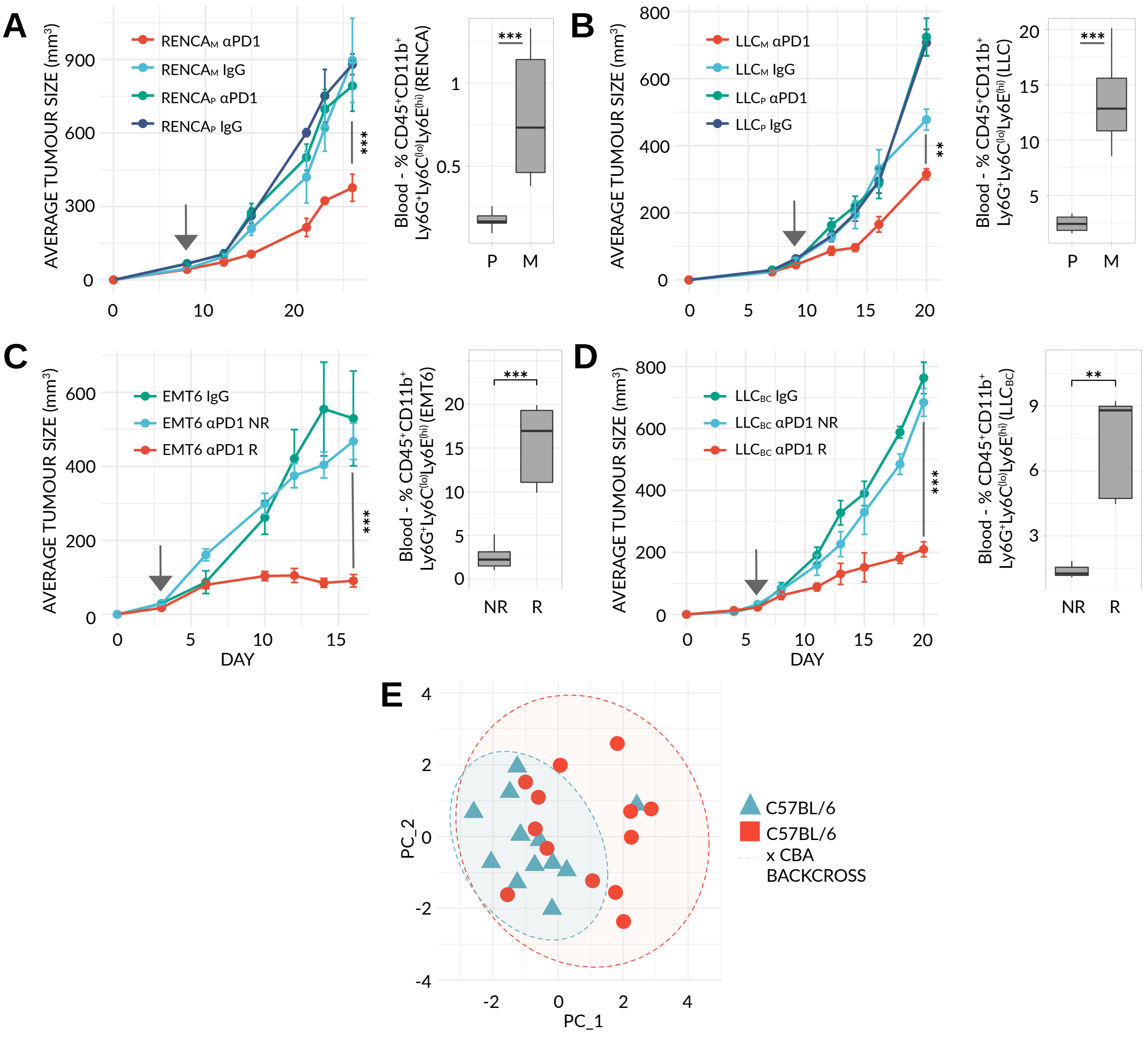
A
RENCA$_M$ αPD1
RENCA$_M$ IgG
RENCA$_P$ αPD1
RENCA$_P$ IgG
AVERAGE TUMOUR SIZE (mm^3)
Blood - % CD45$^+$CD11b$^+$ Ly6G$^+$Ly6C$^{(lo)}$Ly6E$^{(hi)}$ (RENCA)
P
M
B
LLC$_M$ αPD1
LLC$_M$ IgG
LLC$_P$ αPD1
LLC$_P$ IgG
Blood - % CD45$^+$CD11b$^+$ Ly6G$^+$Ly6C$^{(lo)}$Ly6E$^{(hi)}$ (LLC)
C
EMT6 IgG
EMT6 αPD1 NR
EMT6 αPD1 R
DAY
Blood - % CD45$^+$CD11b$^+$ Ly6G$^+$Ly6C$^{(lo)}$Ly6E$^{(hi)}$ (EMT6)
NR
R
D
LLC$_{BC}$ IgG
LLC$_{BC}$ αPD1 NR
LLC$_{BC}$ αPD1 R
Blood - % CD45$^+$CD11b$^+$ Ly6G$^+$Ly6C$^{(lo)}$Ly6E$^{(hi)}$ (LLC$_{BC}$)
E
PC_1
PC_2
C57BL/6
C57BL/6
x CBA
BACKCROSS

Figure S9. Ly6E$^{(hi)}$ neutrophils mark immunotherapy response in diverse mouse tumor models, related to Figure 5. **(A-D)** Averaged tumor growth profiles for: BALB/c mice implanted with parental (RENCA$_P$) or mutagenized (RENCA$_M$) renal cell carcinoma (n=6 mice/group) **(A)**; C57BL/6 mice implanted with parental (LLC$_P$) or mutagenized (LLC$_M$) Lewis lung carcinoma (n=6 mice/group) **(B)**; BALB/C mice implanted with spontaneously responding EMT6 breast cancer (IgG n=5, αPD1 n=45) **(C)** and C57BL/6 x CBA backcrossed mice implanted with parental Lewis lung cancer (LLC) (IgG n=5, αPD1 n=15) **(D)**. In all cases, the frequencies of Ly6E$^{(hi)}$ neutrophils in the blood of each model are shown (right) as determined by flow cytometry. In the case of (C-D) unsupervised, hierarchical clustering and pairwise comparison to respective control, IgG-treated mice was utilized to segregate mice into non-responding (NR) and responding (R) groups. In all cases, treatment was initiated at a tumor size of ~50mm^3 (arrow). *Significance was assessed by means of two-sample KS-test (growth) and a Mann-Whitney test (flow) (**, p<0.001; ***, p<0.0001).* **(E)** Principal component analysis, based on the frequencies of all major immune cell types (NK, B, CD8/4$^+$ T cells, monocytes, granulocytes) in the blood of non-tumor bearing C57BL/6 (n=13) and C57BL/6 x CBA backcross (n=13) mice, as determined by flow cytometry and expressed as a % of CD45$^+$ cells. *95% confidence intervals are shown (ellipses).*

FIGURE S10

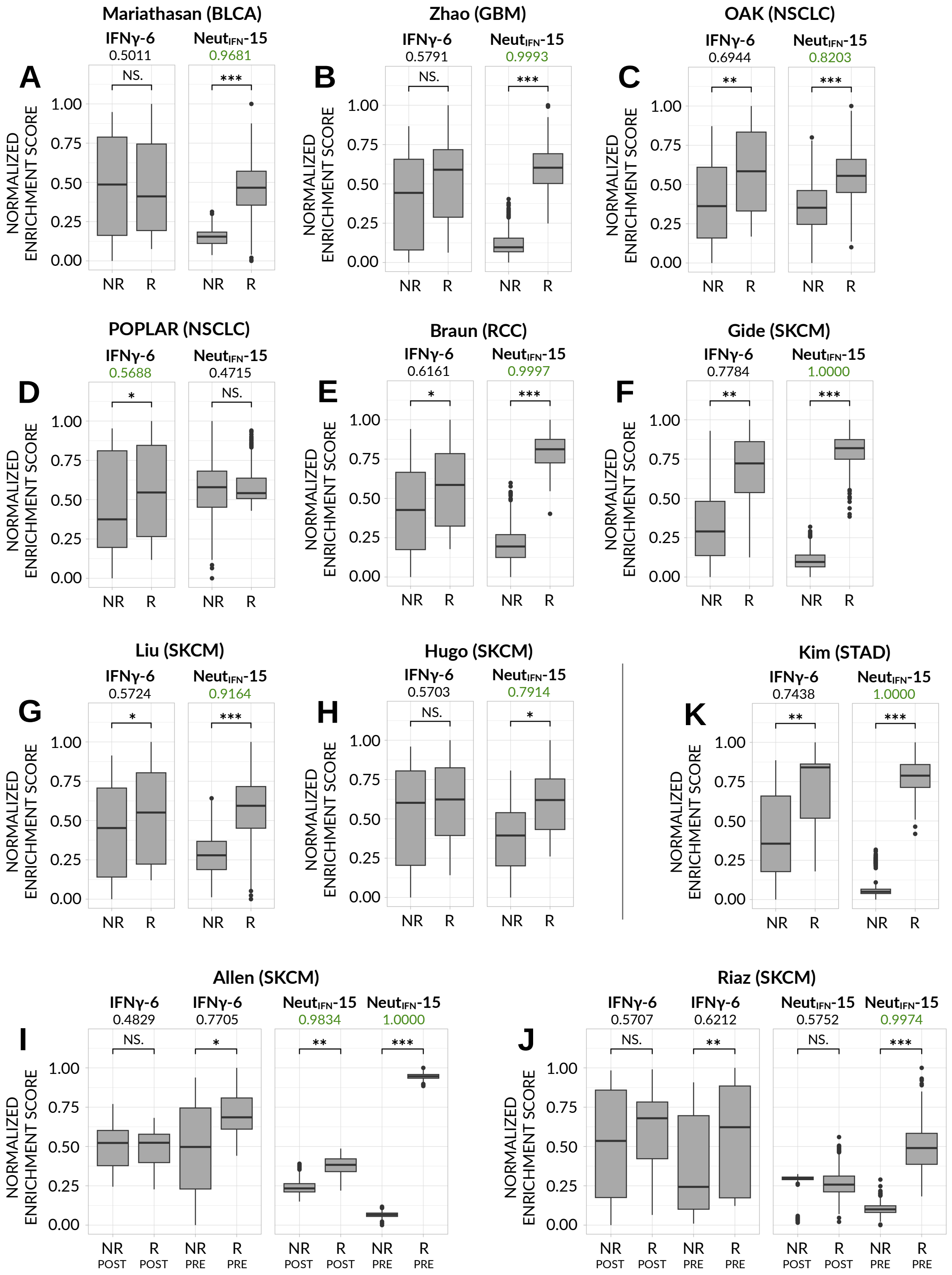

Mariathasan (BLCA)
Zhao (GBM)
OAK (NSCLC)
POPLAR (NSCLC)
Braun (RCC)
Gide (SKCM)
Liu (SKCM)
Hugo (SKCM)
Kim (STAD)
Allen (SKCM)
Riaz (SKCM)
IFNγ-6
Neut$_{IFN}$-15
NORMALIZED ENRICHMENT SCORE
NR
R
POST
PRE

Figure S10. A Ly6E$^{(hi)}$ neutrophil-derived gene signature significantly outperforms the previously published IFNγ-6 signature, related to Figure 7. Boxplots depicting raw enrichment scores obtained from the IFNγ-6 signature (Left) and the Ly6E$^{(hi)}$ Neut$_{IFN}$-15 signature (Right). AUC values are shown for each dataset and signature, denoting the ability of the scores to stratify responders and non-responders. The highest performing signature within each dataset is denoted in green. *Significance was assessed by means of a one-way Mann-Whitney test (NS, p>0.01; *, p<0.01; **, p<0.001, ***, p<0.0001).*
